# Transcriptome- and genome-wide systematic identification of expansin gene family and their expression in tuberous root development and stress responses in sweetpotato (*Ipomoea batatas*)

**DOI:** 10.3389/fpls.2024.1412540

**Published:** 2024-06-20

**Authors:** Jianling Zhang, Tingting Dong, Mingku Zhu, Dan Du, Ranran Liu, Qianqian Yu, Yueying Sun, Zhihuan Zhang

**Affiliations:** ^1^ Laboratory of Plant Germplasm Resources Innovation and Utilization, School of Life Sciences, Liaocheng University, Liaocheng, Shandong, China; ^2^ Institute of Biotechnology, Qingdao Academy of Agricultural Sciences, Qingdao, Shandong, China; ^3^ Institute of Integrative Plant Biology, School of Life Science, Jiangsu Normal University, Xuzhou, Jiangsu, China; ^4^ College of Horticulture and Landscape Architecture, Southwest University, Chongqing, China

**Keywords:** sweetpotato, expansin gene family, systematic identification, RNA-seq, tuberous root development, plant hormones, stress response

## Abstract

**Introduction:**

Expansins (EXPs) are essential components of the plant cell wall that function as relaxation factors to directly promote turgor-driven expansion of the cell wall, thereby controlling plant growth and development and diverse environmental stress responses. EXPs genes have been identified and characterized in numerous plant species, but not in sweetpotato.

**Results and methods:**

In the present study, a total of 59 EXP genes unevenly distributed across 14 of 15 chromosomes were identified in the sweetpotato genome, and segmental and tandem duplications were found to make a dominant contribution to the diversity of functions of the IbEXP family. Phylogenetic analysis showed that IbEXP members could be clustered into four subfamilies based on the EXPs from *Arabidopsis* and rice, and the regularity of protein motif, domain, and gene structures was consistent with this subfamily classification. Collinearity analysis between *IbEXP* genes and related homologous sequences in nine plants provided further phylogenetic insights into the EXP gene family. Cis-element analysis further revealed the potential roles of *IbEXP* genes in sweetpotato development and stress responses. RNA-seq and qRT-PCR analysis of eight selected *IbEXPs* genes provided evidence of their specificity in different tissues and showed that their transcripts were variously induced or suppressed under different hormone treatments (abscisic acid, salicylic acid, jasmonic acid, and 1-aminocyclopropane-1-carboxylic acid) and abiotic stresses (low and high temperature).

**Discussion:**

These results provide a foundation for further comprehensive investigation of the functions of *IbEXP* genes and indicate that several members of this family have potential applications as regulators to control plant development and enhance stress resistance in plants.

## 1 Introduction

The cell wall is a crucial component of plant cells. Owing to its dynamic structure and extensibility, it can determine and maintain cell size and shape and serves as a protective barrier (Cosgrove, 2005; 2015a; Höfte and Voxeur, 2017). The plant cell wall is a highly complex structure constituted by various polysaccharides that vary in abundance, function, and structure (Santiago et al., 2018), and it has crucial roles in providing supplies of stiffness and mechanical support to the plant body, resistance to abiotic and biotic stresses, conduction of nutrients and water, and determination of plant architecture and morphogenesis (Zhang et al., 2021b). The study of cell wall extension mechanisms has become a research priority owing to the significance of cell wall enlargement during plant morphogenesis (Lin et al., 2011). Increases in cell volume and quantity that depend on cell wall enlargement and loosening are crucial for plant growth (Cosgrove, 2015b). During cell wall loosening, an important precondition of cell wall remodeling, the physical structure of the cell wall is altered or new components are added, inducing alterations in shape and anisotropic growth of the cell (Cosgrove, 2015b). Modified proteins that attach to the cell wall have vital roles in cell wall enlargement and loosening, and the most widely recognized of these proteins are the expansins (EXPs) (Yang et al., 2021).

EXPs proteins are commonly found in plants and are crucial for cell expansion. They participate in cell wall enlargement and loosening (Cosgrove, 2000). As the primary factor in enlargement and loosening, EXP genes can control cell relaxation without any chemical energy through non-enzymatic activity (Cosgrove, 2015b). In addition, they can act directly on the plant cell wall to loosen it via binding to cellulose, thereby disrupting hydrogen bonds in wall matrix polysaccharides and cellulose microfibrils in a pH-dependent manner, and they also take part in the decomposition, remodeling, extension, and assembly of the cell wall (Mcqueen-Mason and Cosgrove, 1995; Cosgrove, 2000; 2015b; Marowa et al., 2016). Plant EXPs are usually composed of a signal peptide at the N-terminus (about 20–30 amino acid residues) and two domains. Domain I is a six-stranded double-psi beta-barrel (DPBB) located at the N-terminus. It shows homology with the catalytic domain of GH45 proteins (glycoside hydrolase family 45) and harbors a conserved His-Phe-Asp (HFD) motif, but does not have the β-1, 4-glucanase activity (Yennawar et al., 2006). This region is rich in Cys residues with a characteristic catalytic domain that may be related to disulfide bond formation (Jin et al., 2020). Domain II, which harbors a β-sandwich fold and shares about 50% similarity with the group-II pollen allergen protein (pollen_allerg_1, G2A family), is considered to be a polysaccharide-binding domain as it contains conserved aromatic amino acids and polar tryptophan residues on its surface. It comprises 90–120 amino acid residues and has been classified as a family-63 carbohydrate binding module (CBM63) (Georgelis et al., 2012; Cosgrove, 2015b).

According to standardized nomenclature and phylogenetic analysis, plant EXP proteins can be divided into four subfamilies: EXPA (α-expansin), EXPB (β-expansin), EXLA (expansin-like A), and EXLB (expansin-like B) (Kende et al., 2004; Sampedro and Cosgrove, 2005). Numerous members of these four subfamilies have been identified in plants. Among them, EXPA and EXPB have been widely studied and found to participate in cell expansion and plant developmental processes via their wall-loosening activities (Kende et al., 2004; Sampedro and Cosgrove, 2005). By contrast, members of the EXLA and EXLB subfamilies mainly have functions in stress response, hypocotyl length, and root architecture (Boron et al., 2014; Kong et al., 2019; Zhang et al., 2021a). Based on previous investigations, EXP proteins are regarded as the main determinant of cell shape in many cell developmental processes and have particular importance in the regulation of cell-wall extensibility (Li et al., 2003; Choi et al., 2006, 2008), including elongation and expansion (Ashwin Narayan et al., 2021). Since their first identification in cucumber hypocotyl (Mcqueen-Mason et al., 1992), EXP proteins have been found in numerous plant species.

The ability of EXPs to regulate cell wall modification and elongation means they have crucial functions in multiple biological processes, including response to biotic and abiotic stress, root and fiber development, root nodule formation, fruit development and ripening, and other developmental processes (Choi et al., 2006, 2008; Cosgrove, 2015b; Marowa et al., 2016). For instance, overexpression of the *Osmanthus fragrans OfEXLA1* gene has been shown to increase resistance to salt and drought stress in *Arabidopsis* (Dong et al., 2023). Ectopic overexpression of wild *Arachis AdEXLB8* in tobacco increased tolerances to biotic (*Meloidogyne incognita* and *Sclerotinia sclerotiorum*) and abiotic (drought) stresses (Brasileiro et al., 2021). Wheat *TaEXPA2* can significantly elevate the resistance of transgenic plants to Cd toxicity and multiple abiotic stresses (drought, oxidative, and salt) (Chen et al., 2017; Ren et al., 2018; Chen et al., 2018b; Yang et al., 2020). Ectopic expression of poplar *PttEXPA8* in tobacco enhances heat resistance in transgenic plants (Liu et al., 2016). Three EXP genes, tomato *SlExp1*, apple *MdEXLB1*, and mango *MiExpA1*, have been identified as crucial determinants of fruit softening and ripening (Sane et al., 2005; Kaur et al., 2010; Minoia et al., 2016; Chen et al., 2022). Two β-expansin genes, *GmINS1* and *GmEXPB2*, have significant roles in nodule formation and development (Li et al., 2015; Yang et al., 2021). Rice *OsEXPB2* and *OsEXPA8* and soybean *GmEXLB1* and *GmEXPB2* function as important regulators in root system architecture (Guo et al., 2011; Wang et al., 2014; Li et al., 2015; Zou et al., 2015; Kong et al., 2019). *Stylosanthes SgEXPB1*, rice *OsEXPA10*, and *Arabidopsis AtEXPA7* are required for root development (Lin et al., 2011; Che et al., 2016; Wang et al., 2023). Upregulation of *GhEXPA8* or *GbEXPATR* can increase the fiber length in cotton, while reduced EXPA expression results in shorter fibers (Li et al., 2016). In addition, plant EXP genes also play vital roles in height and leaf growth (Pien et al., 2001; Ma et al., 2013), pollen tube and stem elongation (Choi et al., 2003; Gray-Mitsumune et al., 2008; Liu et al., 2021b), seed development, germination, and yield (Chen and Bradford, 2000; Chen et al., 2001; Calderini et al., 2021), flower development (Zenoni et al., 2004), and so on.

Sweetpotato (*Ipomoea batatas*) is the only Convolvulaceae crop that generates starch storage roots (Liu, 2017; Arisha et al., 2020). It is considered the seventh most important food crop and is widely cultivated worldwide owing to its numerous advantages, which include low input requirements, strong stress resistance, wide adaptability, and high yield and starch content (Ahn et al., 2010). Sweetpotato has broad applications in alcohol and starch production, animal feed, starch processing, and human food. It also ensures food security in many developing countries on account of its ability to adapt to various environmental conditions (Liu, 2017). In a previous study, 37 EXP genes were identified in *Ipomoea trifida*, which is the most likely diploid wild relative of sweetpotato (Li et al., 2022). However, the *I. trifida* genome does not adequately represent the whole sweetpotato genome. Recently, the completion of hexaploid sweetpotato genome sequencing has provided sufficient and valuable information for the identification and characterization of gene families (Yang et al., 2017). However, there has been a lack of genome-wide identification of the sweetpotato EXP gene family. Therefore, in this study, we comprehensively identified and characterized the EXP genes in sweetpotato.

A major focus in crop molecular breeding at present involves improving environmental stress resistance to promote plant growth. Investigations have been performed systematically in a variety of plant species owing to the relevance of the crucial functions of EXP genes to the demands of breeding. A large number of EXP genes have been identified in a diverse range of plants. For instance, in monocots, 92, 58, 241, 88, 46, and 38 genes have been identified in sugarcane (Santiago et al., 2018), rice (Sampedro and Cosgrove, 2005), common wheat (Han et al., 2019), maize (Zhang et al., 2014), barley (Liu et al., 2021a), and *Brachypodium distachyon* (Chen et al., 2020b), respectively; and in dicotyledons, 36, 75, 46, 93, 52 genes were found in *Arabidopsis* (Sampedro and Cosgrove, 2005), soybean (Zhu et al., 2014), gingkgo (Guo et al., 2023), cotton (Lv et al., 2020), and tobacco (Ding et al., 2016), respectively. The genome sequencing of hexaploid sweetpotato (Taizhong6) has been completed (Yang et al., 2017). However, no systematic identification and characterization of EXP genes in sweetpotato (*I. batatas* L.) is yet available. The identification of molecular features of significant members of the EXP gene family will contribute to further understanding of the regulatory mechanisms of plant development and adaptation to environmental stresses. In this study, 59 *IbEXP* genes, which were divided into four subfamilies (36 *IbEXPA*, ten *IbEXPB*, two *IbEXLA*, and 11 *IbEXLB* genes), were identified from the sweetpotato genome. To standardize the nomenclature of EXP proteins in sweetpotato and evaluate their possible functions and relationships in development and stress responses, we performed a comprehensive and systematic characterization of these 59 identified sweetpotato EXP genes. Phylogenetic relationships, chromosomal location, conserved motif and domain, gene structure, molecular characteristics, cis-elements, gene duplications, and expression patterns of *IbEXPs* in different tissues and under various hormone treatments and abiotic stresses were investigated. The data presented here represent a foundation for further screening and functional investigation of valuable IbEXP genes with crucial roles in tuberous root development and stress tolerances in sweetpotato.

## 2 Materials and methods

### 2.1 Identification of *IbEXP* genes in sweetpotato

Genome data and GFF annotation files for sweetpotato were obtained from the online *Ipomoea* Genome Hub database (http://sweetpotao.com) (Yang et al., 2017). Protein sequences of EXPs from rice and *Arabidopsis* were downloaded from the Rice Genome Annotation Project database (http://rice.plantbiology.msu.edu/) and the *Arabidopsis* Information Resource (TAIR) (https://www.arabidopsis.org/), as described in a previous study (Sampedro and Cosgrove, 2005). Then, the *Arabidopsis* and rice EXP protein sequences were used as query sequences to carry out BlastP against all of the *I. batatas* protein sequences to identify all possible EXP members in sweetpotato using the TBtools software with an E-value ≤ 1e^-5^, NumofHits 500, and NumofAligns 250 (Chen et al., 2020a). Subsequently, the PROSITE database (https://prosite.expasy.org/), Pfam database (http://pfam.xfam.org/), and NCBI batch CD-search (https://www.ncbi.nlm.nih.gov/Structure/bwrpsb/bwrpsb.cgi) were used to confirm all the candidate IbEXP proteins obtained in this way and to exclude protein sequences that lacked the EXP domain.

### 2.2 Sequence alignment, phylogenetic analysis, and nomenclature of IbEXP proteins

The previously reported 36 *Arabidopsis* AtEXP protein sequences and 58 rice OsEXP protein sequences (Sampedro and Cosgrove, 2005), together with our 59 sweetpotato IbEXP protein sequences, were used to perform phylogenetic tree analysis. First, 153 EXP protein sequences were subjected to multiple sequence alignment using ClustalW with default parameters. Then, MEGA software (version 11.0) was used to construct an unrooted phylogenetic tree using the neighbor-joining bootstrap method. The detailed parameters were as follows: Poisson model, pairwise deletion, and 1,000 replicates. The *Arabidopsis* and rice EXP protein sequences are provided in **Supplementary File 1**. For the naming method of *IbEXP* genes, these 59 *IbEXP* genes were divided into four subfamilies (i.e., EXPA, EXPB, EXLA, and EXLB) according to the homology of IbEXP protein sequences with *Arabidopsis* AtEXP and rice OsEXP protein sequences. Then, the *IbEXP* members in each subfamily were named based on their position on the chromosome.

### 2.3 Analysis of motif patterns, conserved domains, protein properties, and interactions of IbEXPs

The online MEME Suite (version 5.5.4, https://meme-suite.org/meme/tools/meme) and the Batch CD-Search database (https://www.ncbi.nlm.nih.gov/Structure/bwrpsb/bwrpsb.cgi) were employed to explore the motifs and conserved domains (standard results) of each IbEXP protein, respectively. The ExPASy database (http://expasy.org/) was used to evaluate the properties of each IbEXP protein, including the Mw (molecular weight) and pI (theoretical isoelectric point). Subcellular locations and phosphorylation sites of each IbEXP protein were also predicted using the online databases Plant-mPLoc (http://www.csbio.sjtu.edu.cn/bioinf/plant-multi/) and NetPhos 3.1 (https://services.healthtech.dtu.dk/service.php?NetPhos-3.1), respectively. Subsequently, the STRING 12.0 website (https://cn.string-db.org/) was used to construct the potential protein-interacting network.

### 2.4 Chromosomal location and collinearity analysis of *IbEXP* genes

The locations of *IbEXP* genes on the chromosomes of sweetpotato were investigated using GFF annotation information obtained from the *Ipomoea* genome website (https://sweetpotao.com/). For analysis of the synteny between *IbEXP* genes and EXP genes in other plants, the genome and GFF annotation files for *I. batatas* and another nine representative plant species (*Ipomoea triloba*, rice, *Arabidopsis*, maize, wheat, *Brassica rapa*, pepper, *Brassica oleracea*, and tomato) were downloaded from TAIR, the *Ipomoea Genome* Hub, Sol Genomics Network, EnsemblPlants (http://plants.ensembl.org/index.html), and Phytozome (https://phytozome-next.jgi.doe.gov/). The collinearity relationships and gene duplications were investigated using the Multiple Collinearity Scan toolkit (MCScanX) with default parameters (Wang et al., 2012). Then, circos and TBtools software with 30 as the minimum block size were employed to visualize these results (Krzywinski et al., 2009; Chen et al., 2020a).

### 2.5 Transcriptome-wide analysis of genes related to tuberous root development

The sweetpotato (*I. batatas* L.) Taizhong 6 plants were cultivated in the greenhouse of Jiangsu Normal University. The fibrous roots (FR), developing tuberous roots (DR), and mature tuberous roots (MR) of the plants were collected, and RNA was extracted. Each sample involved roots from at least six different sweetpotato plants at the same developmental stage. Then, the four types of samples were used to perform the RNA sequencing (RNA-seq) analysis and transcriptome analysis on the Illumina Novaseq™ 6000 platform (LC Bio Technology Co., Ltd., Hangzhou, China). The clean reads were aligned with the sweetpotato genome database (https://sweetpotao.com/). StringTie was used to calculate FPKM values for mRNAs (FPKM = [total_exon_fragments/mapped_reads(millions) × exon_length(kB)]); these were then used to estimate gene expression levels. Genes with |log2 (fold change)| ≥1 and p-value <0.05, as determined by DESeq2, were considered to be differentially expressed genes (DEGs) between different groups. Finally, DAVID software (https://david.ncifcrf.gov/) was used for gene ontology (GO) and Kyoto Encyclopedia of Genes and Genomes (KEGG) enrichment analysis of the DEGs. Gene annotation and DEG analysis were carried out as described in our previous study (Li et al., 2024). The sequencing data have been deposited at the NCBI Sequence Read Archive (http://www.ncbi.nlm.nih.gov/Traces/sra) with accession numbers GSM7838330, GSM7838331, and GSM7838332.

### 2.6 Plant materials, abiotic stress, and hormone treatments

The Taizhong 6 sweetpotato (*I. batatas*) plants were cultivated in a greenhouse at Jiangsu Normal University. Then, the developing tuberous roots (DR) at 30 days after planting (dap; DR1), 60 dap (DR2), and 100 dap (DR3), mature tuberous roots (MR) at 120 dap, mature leaves (L) at 60 dap, and stems (S) at 60 dap were collected, and the expression patterns of selected *IbEXP* genes were analyzed. For treatment with different hormones and abiotic stresses, *I. batatas* seedlings (XuShu 22) harboring 3–4 mature leaves were cultured in a greenhouse. After a week of cultivation in water (1/4 Hoagland solution), consistent seedlings were selected for treatment. For the hormone treatments, the seedlings were cultivated and sprayed with abscisic acid (ABA, 100 μM), 1-aminocyclopropane-1-carboxylic acid (ACC, 100 μM), jasmonic acid (JA, 100 μM), and salicylic acid (SA, 2 mM), respectively. For abiotic stresses, seedlings were cultivated at 4°C (LT) and 42°C (HT) to simulate cold and heat stresses, respectively. All leaf samples taken from plants in the control and treatment groups were harvested at 0, 1, 12, 24, and 48 h after treatments. At least three independent biological replicates were collected for each treatment.

### 2.7 RNA extraction and quantitative real-time PCR analysis

Total RNA was extracted from each collected sample using the RNA Extraction Kits (OMEGA, USA) following the method provided by the manufacturer. First-strand cDNA was synthesized via reverse transcription using 800 ng of total RNA following the methods used in our study (Zhang et al., 2018). Then, the synthesized cDNAs were diluted with RNase/DNase-free water, and qRT-PCR was performed using the CFX96™ Real-Time System (Bio-Rad, USA) as previously described (Zhang et al., 2018). Sweetpotato *IbARF* (accession number: JX177359) was used as the reference gene (Chen et al., 2018a). All primers in qRT-PCR were listed in **Supplementary Table S6**. Three independent technical and biological repetitions were performed for each sample.

### 2.8 Statistical analyses

Data were presented as mean ± SE standard deviation. A cut-off two-fold value for differential gene expression was considered to indicate biological significance (Liu et al., 2022). OriginPro software (v8.0, SAS Institute) was used to visualize the results of qRT-PCR experiments.

## 3 Results

### 3.1 *IbEXP* gene identification and characterization in sweetpotato

In this study, a total of 59 EXP genes were identified from the sweetpotato genome and named following the classification scheme used for *AtEXP* and *OsEXP* genes in *Arabidopsis* and rice, respectively, based on their position on the chromosome. The nucleotide and protein sequences for each of these IbEXPs can be found in **Supplementary File 1**. Protein length (number of amino acids [aa]), pI, Mw, and phosphorylation sites were determined for each protein. Detailed data are shown in **Table 1**. The protein length and Mw of IbEXPs varied widely, with the length ranging from 183 aa (IbEXPA31) to 670 aa (IbEXPA19) and the Mw ranging from 20.1739 kD (IbEXPB31) to 74.052 kD (IbEXPA19). The pI ranged from 4.63 (IbEXLB11) to 9.82 (IbEXPA34). Subcellular location prediction showed that most IbEXPs were located on the cell wall, and very few were located in the nucleus (IbEXPA19) or in both the chloroplast and the cell wall (IbEXPA22 and IbEXPA33). Phosphorylation site prediction of IbEXPs indicated significant variation from 24 sites (IbEXPA17 and IbEXPA31) to 109 (IbEXPA12), and the vast majority of IbEXPs harbored more Ser sites than Tyr or Thr sites. Moreover, over 83.05% of IbEXPs had at least 30 phosphorylation sites.

**Table 1:** Characteristics of IbEXP proteins in *Ipomoea batatas*.

Gene name	Gene ID	Amino acids	MW (Da)	PI	Subcellular location	phosphorylation cite			
						Ser site (S)	Tyr cite (Y)	Thr cite (T)	Total
IbEXPA1	g1306.t1	238	25431.71	9.53	Cell wall.	20	3	8	31
IbEXPA2	g3925.t1	251	26960.41	8.07	Cell wall.	14	4	8	26
IbEXPA3	g3926.t1	251	26740.92	8.36	Cell wall.	21	4	6	31
IbEXPA4	g4923.t1	260	28213.08	9.27	Cell wall.	26	3	7	36
IbEXPA5	g9889.t1	310	33660.23	9.25	Cell wall.	26	5	20	51
IbEXPA6	g10004.t1	258	27595.27	9.3	Cell wall.	19	3	4	26
IbEXPA7	g15786.t1	327	35627.31	9.29	Cell wall.	45	5	12	62
IbEXPA8	g15816.t1	272	29860.26	9.36	Cell wall.	25	4	12	41
IbEXPA9	g15820.t1	264	28775.77	9.37	Cell wall.	29	4	9	42
IbEXPA10	g16869.t1	263	28645.8	8.56	Cell wall.	17	4	13	34
IbEXPA11	g17886.t1	258	27852.84	9.5	Cell wall.	13	4	10	27
IbEXPA12	g18537.t1	591	63172.9	7.19	Cell wall.	77	12	20	109
IbEXPA13	g18539.t1	341	35684.91	7.05	Cell wall.	57	6	10	73
IbEXPA14	g20283.t1	260	28086.01	9.4	Cell wall.	20	2	12	34
IbEXPA15	g24580.t1	252	27413.57	6.42	Cell wall.	12	4	11	27
IbEXPA16	g25447.t1	260	27406.89	9.16	Cell wall.	45	3	8	56
IbEXPA17	g25606.t1	209	22967.29	9.73	Cell wall.	13	3	8	24
IbEXPA18	g29706.t1	355	39001.06	9.64	Cell wall.	47	2	9	58
IbEXPA19	g29707.t1	670	74052.16	8.27	Nucleus.	72	5	23	100
IbEXPA20	g30029.t1	202	22488.71	9.44	Cell wall.	12	2	12	26
IbEXPA21	g33328.t1	273	28718.04	6.2	Cell wall.	30	5	7	42
IbEXPA22	g38554.t1	493	53544.79	8.24	Cell wall, Chloroplast	53	9	7	69
IbEXPA23	g39420.t1	309	33789.29	9.29	Cell wall.	29	4	20	53
IbEXPA24	g39467.t1	270	29209.17	9.1	Cell wall.	26	5	11	42
IbEXPA25	g42794.t1	248	26037.26	9.34	Cell wall.	16	2	7	25
IbEXPA26	g47278.t1	246	25903.09	8.64	Cell wall.	18	4	3	25
IbEXPA27	g53361.t1	253	27102.64	8.87	Cell wall.	22	3	6	31
IbEXPA28	g53365.t1	563	61262.75	9.32	Cell wall.	56	5	25	86
IbEXPA29	g58047.t1	241	26303.82	6	Cell wall.	23	3	13	39
IbEXPA30	g58048.t1	240	26373.45	9.21	Cell wall.	22	5	14	41
IbEXPA31	g58049.t1	183	20173.91	6.81	Cell wall.	10	2	12	24
IbEXPA32	g58052.t1	388	43264.45	9.2	Cell wall.	38	2	15	55
IbEXPA33	g58053.t1	521	58831.3	6.61	Cell wall, Chloroplast	26	9	27	62
IbEXPA34	g58951.t1	257	27894.88	9.82	Cell wall.	17	2	7	26
IbEXPA35	g60585.t1	237	25641.21	9.35	Cell wall.	20	2	9	31
IbEXPA36	g61698.t1	264	28887.9	9.39	Cell wall.	28	4	10	42
IbEXPB1	g9145.t1	207	21810.79	9.49	Cell wall.	22	1	10	33
IbEXPB2	g24143.t1	243	25960.18	5.75	Cell wall.	31	4	4	39
IbEXPB3	g24144.t1	400	44133.02	7.09	Cell wall.	56	7	21	84
IbEXPB4	g27129.t1	308	33145.35	7.52	Cell wall.	47	5	20	72
IbEXPB5	g27144.t1	265	28079.59	6.19	Cell wall.	37	2	5	44
IbEXPB6	g27334.t1	342	36757.24	5.16	Cell wall.	50	5	7	62
IbEXPB7	g27336.t1	256	27320.01	8.37	Cell wall.	31	5	3	39
IbEXPB8	g48839.t1	266	27877.16	4.89	Cell wall.	35	3	8	46
IbEXPB9	g48841.t1	336	36036.73	6.69	Cell wall.	32	5	6	43
IbEXPB10	g54432.t1	262	28422.34	8.74	Cell wall.	19	1	14	34
IbEXLA1	g904.t1	266	28622.45	5.29	Cell wall.	22	4	9	35
IbEXLA2	g59839.t1	268	29409.37	6.96	Cell wall.	17	4	12	33
IbEXLB1	g14386.t1	252	28275.14	5.45	Cell wall.	12	14	10	36
IbEXLB2	g14427.t1	443	49325.01	8.93	Cell wall.	37	15	21	73
IbEXLB3	g14812.t1	351	38060.5	7.91	Cell wall.	40	10	21	71
IbEXLB4	g15079.t1	284	30949.46	8.57	Cell wall.	29	4	8	41
IbEXLB5	g15300.t1	247	26876.61	8.32	Cell wall.	20	7	6	33
IbEXLB6	g33144.t1	251	28135.78	5.41	Cell wall.	17	15	10	42
IbEXLB7	g33212.t1	278	31034.24	5.52	Cell wall.	17	13	10	40
IbEXLB8	g55672.t1	272	28559.26	6.58	Cell wall.	24	5	17	46
IbEXLB9	g55673.t1	247	27029.36	9.33	Cell wall.	30	8	18	56
IbEXLB10	g55674.t1	246	26984.94	8.46	Cell wall.	16	14	13	43
IbEXLB11	g55709.t1	308	32778.84	4.63	Cell wall.	24	7	12	43

### 3.2 Phylogenetic relationships of IbEXPs in sweetpotato

**Figure 1:**
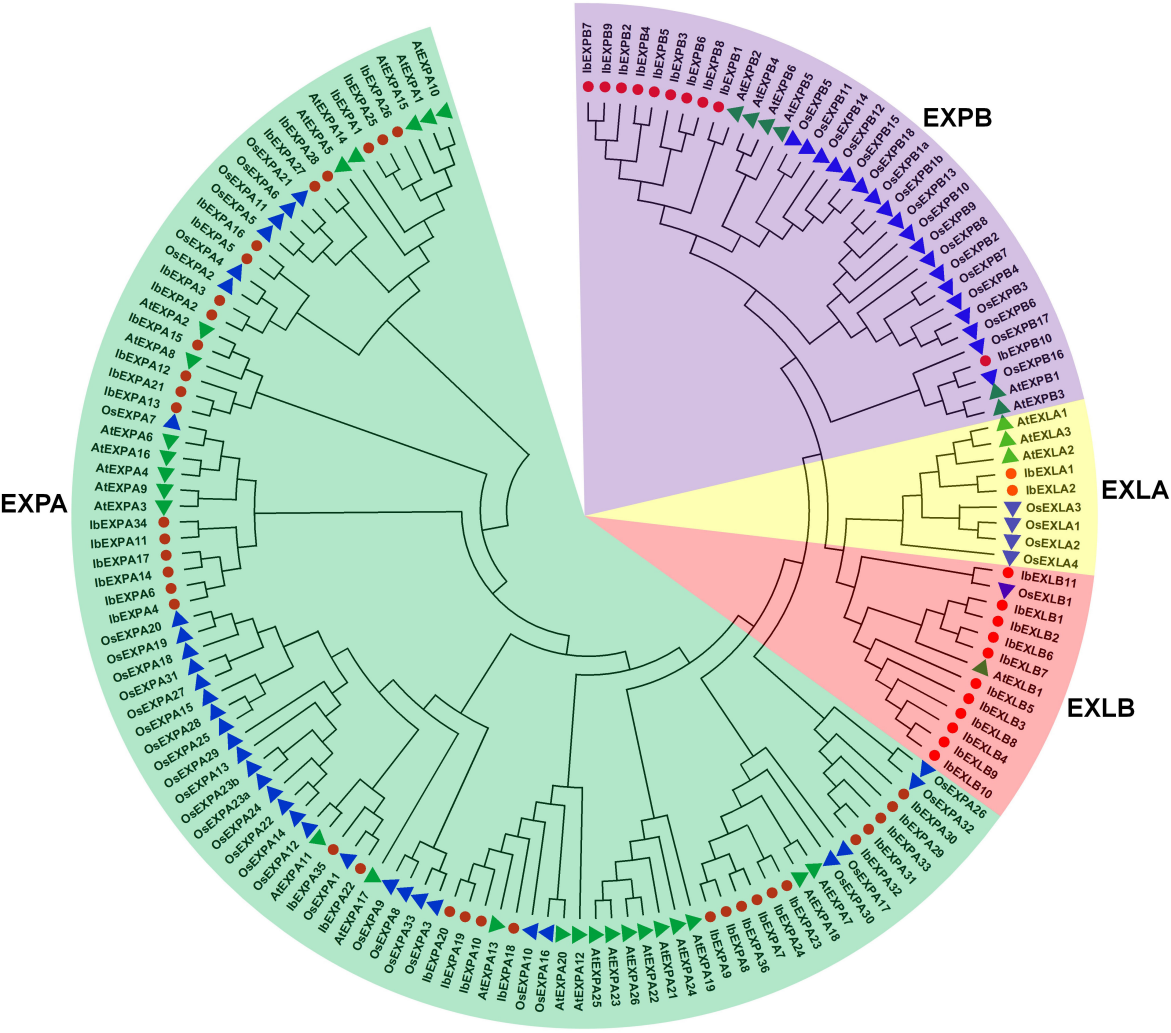
Phylogenetic tree of 59 sweetpotato IbEXP proteins with *Arabidopsis* and rice EXP proteins. The phylogenetic relationships were constructed by the MEGA 11.0 software using the neighbor-joining bootstrap method according to the following parameters: poisson model, pairwise deletion, and 1,000 replicates. Different subfamilies are named following the studies in Arabidopsis and rice, and different colors were used to distinguish each subfamily. Red circles, green triangles, and blue triangles represent sweetpotato IbEXPs, Arabidopsis AtEXPs, and rice OsEXPs, respectively.

To explore the evolutionary relationships of IbEXPs in sweetpotato, phylogenetic analysis was carried out using the protein sequences of the 59 IbEXPs, 36 Arabidopsis AtEXPs, and 58 rice OsEXPs (**Supplementary File 1**; **Supplementary Table S1**). Then, an unrooted phylogenetic tree was constructed using the neighbor-joining bootstrap method with MEGA software (version 11.0). Phylogenetic tree analysis showed that these 59 IbEXPs could be divided into four subfamilies (EXPA, EXPB, EXLA, and EXLB) according to the topology of the tree, clade support values, and reported studies on EXP classification in *Arabidopsis* and rice (Sampedro and Cosgrove, 2005) (**Figure 1**). These four subfamilies are named IbEXPA, IbEXPB, IbEXLA, and IbEXLB, with 36, 10, 2, and 11 members, respectively. The sizes of these subfamilies varied greatly, with the numbers of IbEXPs ranging from 2 to 36. The EXPA subfamily was the largest (36 members), whereas the EXLA subfamily had only two members, consistent with their counterparts in *Arabidopsis* and rice. The differences among IbEXPs, AtEXPs, and OsEXPs in the same subfamilies indicate apparent interspecific divergence of the EXP gene family among sweetpotato, *Arabidopsis*, and rice.

### 3.3 Chromosome localization of sweetpotato *IbEXP* genes

**Figure 2:**
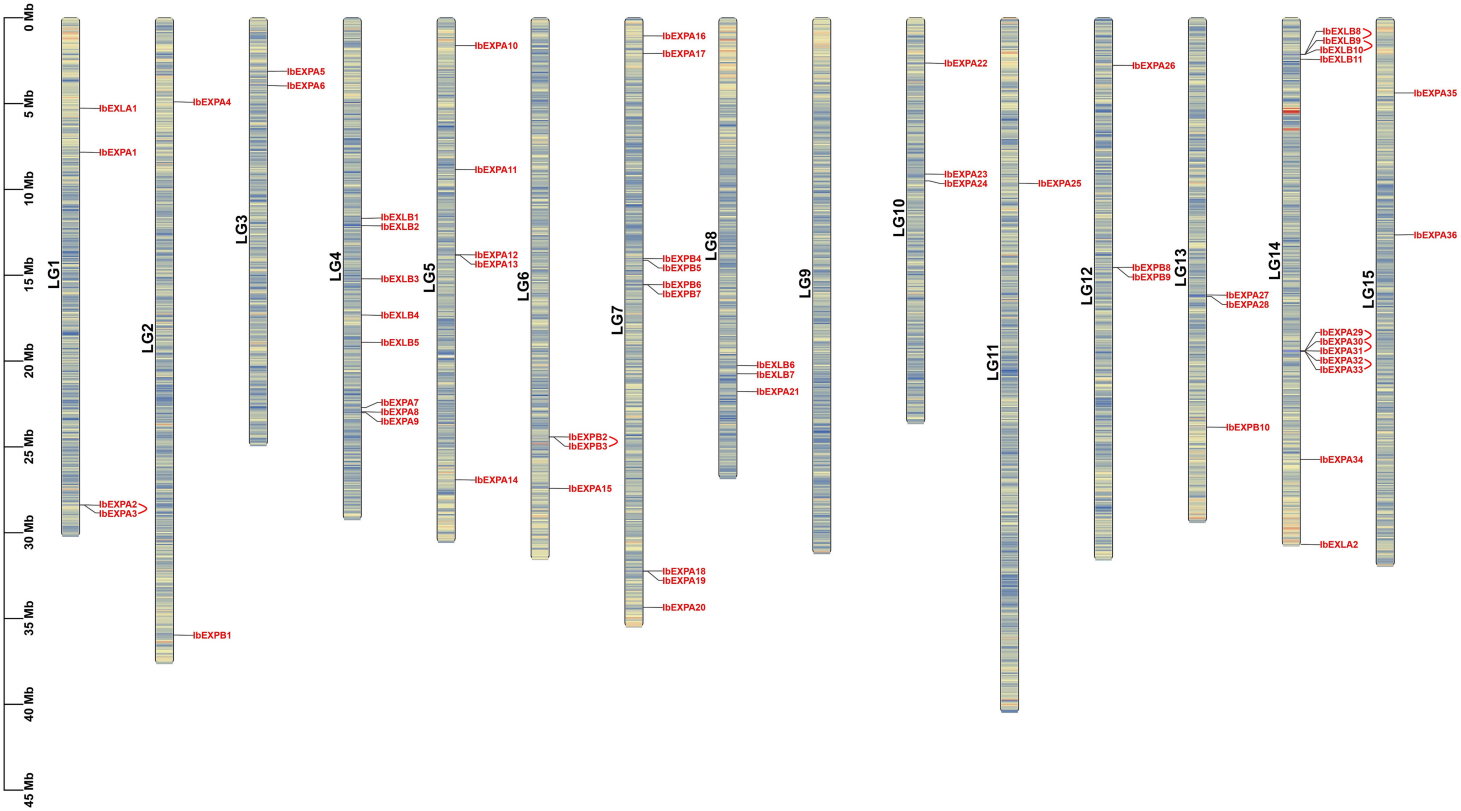
Localizations of 59 *IbEXPs* on sweetpotato chromosomes (LG1-LG15). The red arc behind some *IbEXPs* represents the gene duplication of some EXP genes in sweetpotato.

Chromosome distribution analysis based on sweetpotato GFF3 genome annotations exhibited that 59 *IbEXP* genes were located on 14 of 15 chromosomes of sweetpotato; no *IbEXP* genes were found on LG 9. In general, *IbEXP* genes were unevenly distributed on the 14 chromosomes, possibly owing to uneven gene replications of chromosome fragments. LG 14 contained the most *IbEXP* genes (11), followed by LG 7 with nine, whereas LG 11 had only one. Furthermore, there were eight *IbEXP* genes on LG 4, five on LG 5, and two to four on other chromosomes (**Figure 2**). These results suggest that the *IbEXP* distribution has a highly variable density and is disproportionate to the length of the chromosome. For instance, the largest chromosome (LG 11) contained only one *IbEXP* gene, whereas the smallest chromosome (LG 10) contained three.

### 3.4 Collinearity analysis of sweetpotato *IbEXP* genes

**Figure 3:**
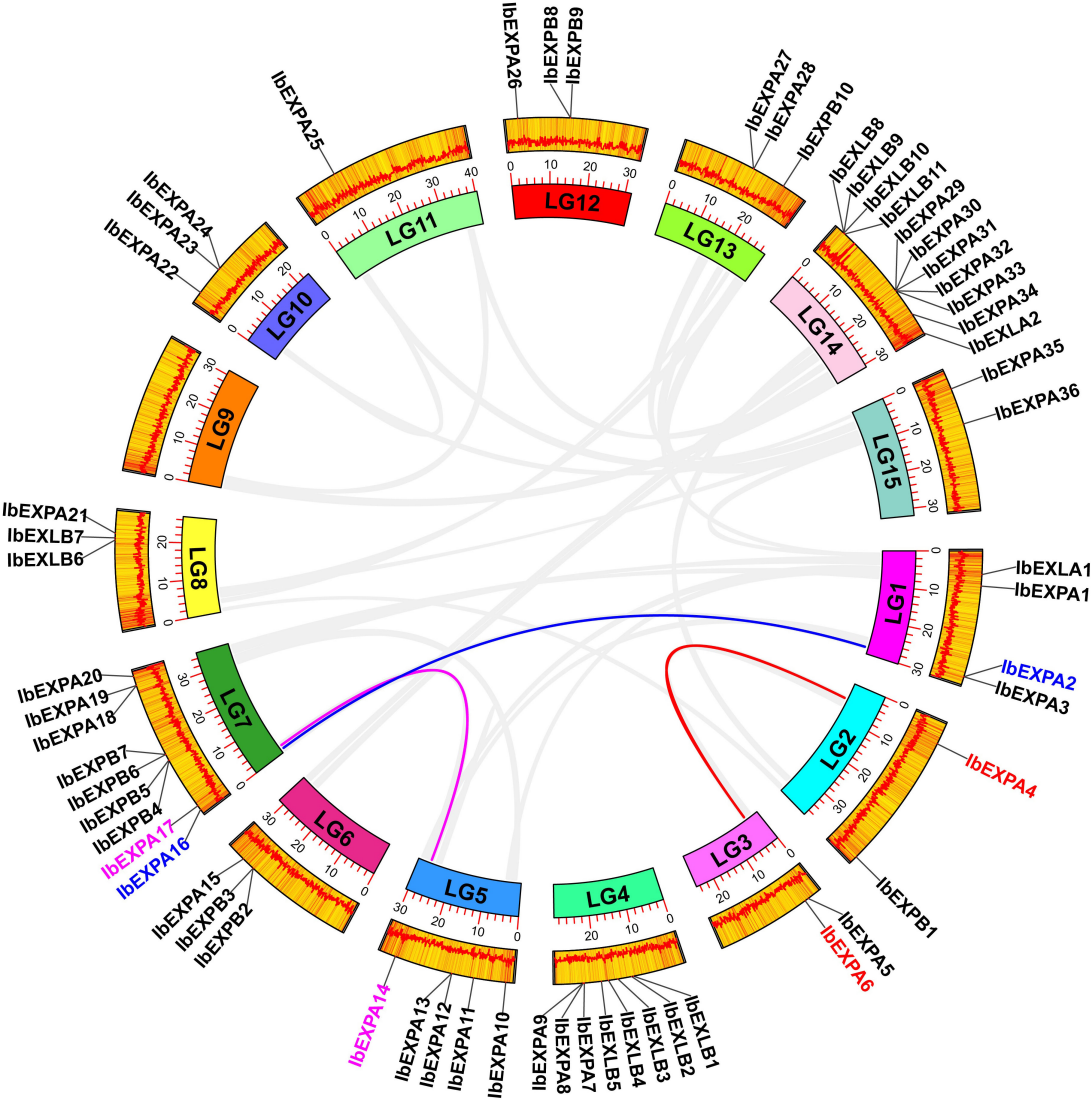
Segmental duplications and collinearity analysis of *IbEXPs* in sweetpotato. LG1–LG15 are represented by different colored rectangles. The heatmap and polyline along each rectangle depict the gene density of each chromosome. Duplicated IbEXP gene pairs on sweetpotato chromosomes are indicated by colored lines, and these corresponding genes are also marked with colors. Other IbEXP genes that exhibit no collinear relationships were marked with a black color.

In plants, genome duplication facilitates the expansion and evolution of gene families (Cannon et al., 2004). To explore potential gene duplications among all 59 *IbEXP* genes, collinearity analysis was performed using the MCScanX and BlastP programs. Seven gene pairs with tandem duplication were identified, comprising *IbEXPA2*-*IbEXPA3*, *IbEXPB2*-*IbEXPB3*, *IbEXLB8*-*IbEXLB9*, *IbEXLB9*-*IbEXLB10*, *IbEXPA29*-*IbEXPA30*, I*bEXPA30*-*IbEXPA31*, and *IbEXPA32*-*IbEXPA33* (**Figure 2**; **Supplementary Table S2–1**). These *IbEXP* genes exhibiting tandem duplications all belonged to the same subfamily. In addition, MCScanX and BlastP were used to identify fragment duplications; the following three gene pairs in the EXPA subfamily were identified on five (LG1–3, LG5, LG7) of the 15 chromosomes: *IbEXPA2*-*IbEXPA16*, *IbEXPA4*-*IbEXPA6*, *IbEXPA14*-*IbEXPA17* (**Figure 3**; **Supplementary Table S2–2**). No fragment duplications of *IbEXPA* genes were found on any other chromosomes (LG4, LG6, and LG8–15), nor were any fragment duplications detected in the other three subfamilies. These segmental duplications occurring only between genes of the EXPA subfamily may partly explain why the EXPA subfamily was larger than the others; it also indicates that the functions of EXPA genes in regulating plant development and response to stress may be more significant than those of genes in the other three subfamilies. In brief, these results suggest that gene duplication is conducive to the expansion of the sweetpotato *IbEXP* gene family.

### 3.5 Collinearity analysis of EXP genes between sweetpotato and other plants

**Figure 4:**
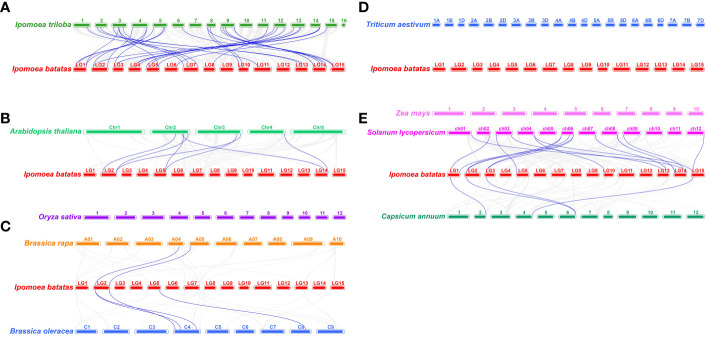
Synteny analyses of sweetpotato IbEXP genes with those of the other nine representative plants. These plants are Ipomoea triloba **(A)**, Oryza sativa and Arabidopsis thaliana **(B)**, Brassica rapa and Brassica oleracea **(C)**, Triticum aestivum and Zea mays **(D)**, Solanum lycopersicum, and Capsicum annuum **(E)**. The chromosomes of various plants are distinguished by their differential colors.

To further investigate the origins and evolutionary relationships of sweetpotato *IbEXP* genes, we explored the collinearity relationships among *IbEXPs* and orthologous genes in nine representative plants, comprising *I. triloba* (the probable diploid wild relative of sweetpotato), two cereal plants, two representative model plants, two Solanaceae plants, and two Brassica plants. A total of 30 (50.8%) *IbEXP* genes showed collinear relationships with orthologous genes of *I. triloba*, followed by 13 for *Solanum lycopersicum*, five for *Arabidopsis*, four for *Capsicum annuum*, three for *B. oleracea* (3), and two for *B. rapa*; there were no collinear relationships of *IbEXP* genes with *Triticum aestivum*, *Oryza sativa*, or *Zea mays* (**Figure 4**; **Supplementary Tables S3–1/-2/-3**). The results thus suggest a closer relationship between sweet potato and *I. triloba*, as the largest number of collinearity relationships existed between the orthologous genes of these two plants. Moreover, we found that multiple *IbEXP* genes had collinear relationships with two genes in other plant species, particularly *I. triloba*. Analogously, two *IbEXP* genes showed collinearity with a single gene of four different plants (*I. triloba*, *Arabidopsis thaliana*, *B. oleracea*, and *S. lycopersicum*) (**Supplementary Tables S3–1/-2/-3**). These data indicate that a number of orthologous genes might originate from a common ancestor in these plants.

### 3.6 Motifs, conserved domains, and gene structure analysis of *IbEXPs*

**Figure 5:**
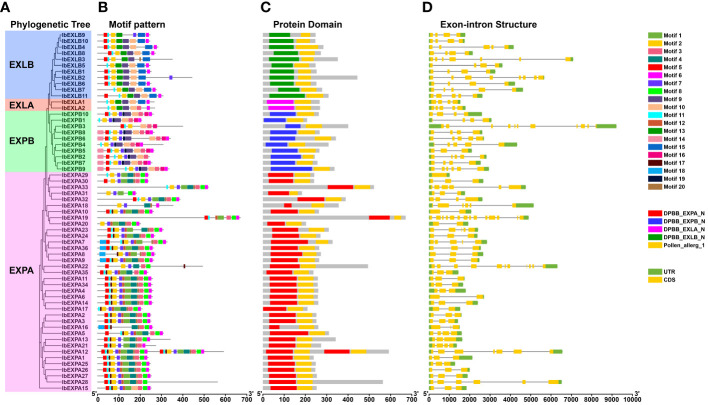
Phylogenetic tree, motif pattern, protein domain, and gene structures of 59 sweetpotato *IbEXPs*. **(A)**. The phylogenetic tree of 59 IbEXP proteins was constructed by MEGA 11.0 based on the consistent parameters used in **Figure 1**. **(B)**. Distributions of motifs in each IbEXP protein. 20 motifs were identified by MEME data. **(C)**. Conserved domain distributions of IbEXP proteins. The CD-search of the NCBI database was used to detect the distributions of conserved domains of IbEXP proteins. The different colorful boxes present diverse conserved domains of each subfamily. **(D)**. Gene structures of 59 sweetpotato IbEXP genes. Green and yellow bars were used to represent the UTR and exons, respectively. The black lines were employed to indicate the introns. The length of IbEXP proteins or genes was estimated using the scale at the bottom.

To further evaluate the sequence characteristics of sweetpotato IbEXPs, we investigated their conserved motif composition. The results showed that a total of 20 distinct motifs were detected based on the sequences of *Arabidopsis* and rice (Sampedro and Cosgrove, 2005). IbEXPs belonging to the same subfamilies generally harbored similar motif compositions, further supporting our subfamily classification (**Figure 5B**). Motifs 2, 5, and 11 were found in most IbEXPs, with the other motifs distributed only in certain IbEXP proteins. Multiple motifs were found in almost all IbEXP members of the same subfamily, with some compositional differences among different subfamilies. For example, almost all members of the EXPA, EXPB, EXLA, and EXLB subfamilies harbored motifs 1, 2, 3, 4, 5, 6, 7, 8, 11, and 20, motifs 2, 3, 5, 7, 10, 11, and 16; motifs 2, 6, 9, 10, 11, and 13, motifs 2, 5, 6, 11, 13, and 15, respectively. Some IbEXPs in the same subfamily contained specific motifs in addition to their common motifs, such as motifs 4, 8, 17, and 18 in EXPA and motifs 14, 16, and 19 in EXPB. These results suggest that the composition and number of motifs vary observably among these four subfamilies, and the existence of specific motifs suggests that sweetpotato IbEXPs may have distinct and diverse functions.

To explore the sequence diversity of *IbEXP* genes, exon-intron composition and conserved domains were examined. Conserved domain examination using Batch CD-Search showed the presence of five domains, comprising one pollen_allerg_1 domain and four typical EXP domains, among all IbEXPs (**Figure 5**). In the same subfamily, most IbEXPs contained the same EXP domain and the pollen_allerg_1 domain, and the EXP domain was located in a similar position, with few exceptions. These data indicate that the EXP domain is the most valuable information to distinctly construct the phylogenetic relationships among IbEXPs. Gene structure detection displayed that the exon numbers of *IbEXP* genes varied from 1 to 14, with one *IbEXP* gene containing no introns and four having only one intron (**Figure 5**). *IbEXPA19* harbored the most exons (14), followed by *IbEXPA22* and *IbEXPB3* with 11 exons each. Moreover, most of the *IbEXP* genes harbored similar gene structures and exhibited similar exon lengths. Some differences in intron numbers among *IbEXP* genes in the same subfamily were found; these may be associated with the functional diversity of *IbEXP* genes. All these results demonstrate that the phylogenetic relationships of IbEXPs are mainly related to their conserved EXP domains and gene structures.

### 3.7 Cis-element prediction in *IbEXP* promoter regions

**Figure 6:**
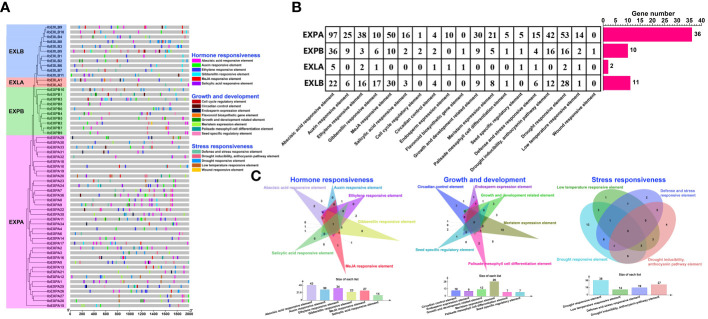
Predicted cis-elements in the promoters of 59 sweetpotato *IbEXPs*. **(A)**. The phylogenetic tree and predicted cis-elements detected from the 2000 bp promoter regions of each IbEXP gene by the PlantCARE database. The same phylogenetic tree as **Figure 5** was used. All cis-elements are classified into three categories: hormone responsiveness, growth and development, and stress responsiveness. **(B)**. The number of cis-elements in the promoter of IbEXP genes. The left table represents the number of each kind of cis-element found in each subfamily. The red rectangles indicate the gene number in each subfamily. **(C)**. Venn diagram of three categories of cis-elements.

Cis-elements, which are located in gene promoter regions, are non-coding sequences. They are vital for gene expression and regulate numerous biological processes (Zhao et al., 2018). To explore the possible regulatory mechanisms by which *IbEXP* genes control plant growth and response to stresses and hormones, we detected cis-elements in the 2000bp sequences upstream of the start codon ATG of each *IbEXP* gene via the PlantCARE database. A total of 742 cis-elements were found in the promoter regions of *IbEXP* genes (**Supplementary Table S4–1**), and these were associated with 19 types of biological processes (**Figure 6**; **Supplementary Table S4–2**). *IbEXLB5* (g15300.t1) contained the largest number (31) of cis-elements, and the numbers of cis-elements in each subfamily varied greatly (**Figure 6**). All detected cis-elements detected in this study could be classified into three categories (**Figure 6**), as follows:

The first category relates to hormone responses (404), including the SA-responsive element (21, 2.83%), MeJA-responsive element (90, 12.13%), gibberellin-responsive element (34, 4.58%), ethylene-responsive element (59, 7.95%), auxin-responsive element (40, 5.39%), and ABA-responsive element (160, 21.56%). All subfamilies of *IbEXP* genes contained abundant ABA-responsive elements (G-box and ABRE), gibberellin-responsive elements (GARE, P-box, TATC-box, and CARE), and ethylene-responsive elements (ERE) in their promoter regions. Members of the EXPA subfamily contained a higher number of ABA-responsive elements, auxin-responsive elements, MeJA-responsive elements, ethylene-responsive elements, and SA-responsive elements compared with the other three subfamilies, whereas members of the EXLA subfamily harbored the fewest of these five hormone-related elements. Notably, only one gibberellin-responsive element was found in the promoter region of EXLA subfamily members. Among these five hormone-responsive elements, the ABA-responsive element was the most frequent, occurring in 43 *IbEXP* genes, followed by the ethylene-responsive element (34 *IbEXP* genes). Some *IbEXP* genes, including *IbEXPA2/8/9/17/23/24/26/34/36*, *IbEXPB3/4*, *IbEXLA1*, and *IbEXLB6/10/11*, had multiple common hormone-responsive elements, indicating the possibility that these members have more rapid and intense responses to certain hormones. Simultaneously, *IbEXPA1/2/8/911/17/24/25/27/31/37/39*, *IbEXPB3/7/8/12*, and *IbEXLB1/2/3/9/10* harbored diverse hormone-responsive elements, suggesting their potential roles in networks of hormone regulation.

The second category relates to growth and development (123), including the cell cycle regulatory element (3, 0.4%), circadian control element (11, 1.48%), endosperm expression element (11, 1.48%), flavonoid biosynthetic gene element (2, 0.26%), growth and development related element (48, 6.47%), meristem expression element (34, 4.58%), seed-specific regulatory element (7, 0.94%), and palisade mesophyll cell differentiation element (7, 0.94%). As shown in **Figures 6B, C**, some cis-elements were absent from certain subfamilies, such as the endosperm expression element, cell cycle regulatory element, flavonoid biosynthetic gene element, growth and development related element, meristem expression element, and seed-specific regulatory element. Cis-elements related to meristem expression were found in the largest number of *IbEXP* genes (26), followed by growth and development-related elements (12 *IbEXP* genes). The EXPA subfamily contained all eight of these elements and had a higher number of cis-elements related to endosperm expression, growth and development, and meristem expression compared with the other three subfamilies. These results indicate that members of the EXPA subfamily may be major regulators during plant growth and development.

The third category relates to stress responsiveness (214), including a defense and stress-responsive element (26, 3.5%), drought inducibility, anthocyanin pathway element (70, 9.43%), drought-responsive element (99, 13.34%), low-temperature responsive element (18, 2.43%), and wound-responsive element (1, 0.13%). Similar to the two categories described above, the EXPA subfamily also contained the largest number of cis-elements related to drought inducibility, defense and stress-responsiveness, anthocyanin pathway, drought response, and low temperature response. The wound-responsive element was absent from the EXPA, EXLA, and EXLB subfamilies, and only one of these elements existed in EXPB. Members in the EXPA subfamilies contained the highest numbers of the drought-responsive element, indicating that EXPA members may be the major regulators of drought response. Moreover, the drought-responsive element was found in the largest number of *IbEXP* genes, suggesting major roles of *IbEXP* genes in drought-responsiveness.

In short, the number and composition of cis-elements in the promoter sequences of different *IbEXP* genes exhibited great diversity within and among subfamilies. These results suggest that *IbEXP* gene expression levels in sweetpotato are controlled by diverse cis-elements in connection with growth and development, hormones, and stress responses.

### 3.8 Protein interaction network analysis for IbEXP proteins in sweetpotato

**Figure 7:**
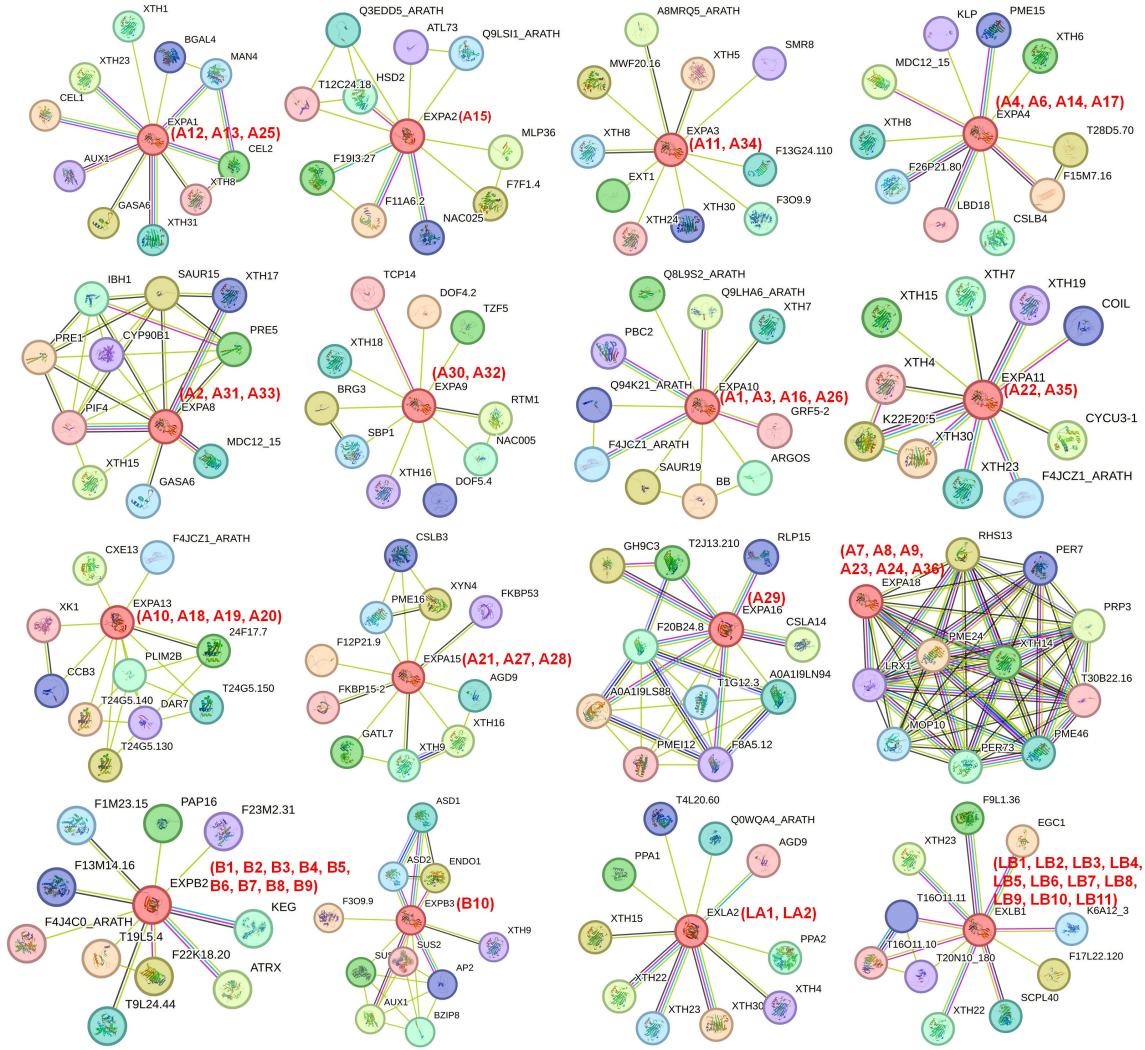
Interaction networks of IbEXP proteins with other functional proteins from different families according to the orthologues in *Arabidopsis*. The amino acid sequences of each IbEXP protein in sweetpotato were employed to search the STRING database according to the orthologues in Arabidopsis. The network node represents proteins, and the edge represents protein-protein associations. The different colored lines between the nodes indicate the different kinds of interactions. The red numbers (IbEXP protein name) in brackets represent the corresponding orthologues in sweetpotato. The filled and empty nodes delineate the proteins with known or predicted 3D structures and unknown 3D structures, respectively.

Exploring the functional relationships of IbEXP proteins could be conducive to uncovering their regulatory networks. Therefore, a protein interaction network for sweetpotato IbEXP proteins was constructed using STRING software based on the orthology analysis of *Arabidopsis* IbEXPs. In general, a few members such as AtEXPA4 (IbEXPA4/6/14/17), AtEXPB3 (IbEXPB10), AtEXPA11 (IbEXPA22/35), and AtEXPA8 (IbEXPA2/31/33) had interaction relationships with other EXP proteins, whereas the others did not (**Supplementary Figure S1**). Furthermore, each AtEXP had interaction relationships with multiple other proteins involved in development and/or stress responses (**Figure 7**). Among these proteins, AtEXPA4 (IbEXPA4/6/14/17) has been reported to be involved in primary root elongation in *Arabidopsis thaliana* (Liu et al., 2021b), AtEXPA8 (IbEXPA2/31/33) and AtEXPB3 (IbEXPB10) are involved in the formation of nematode-induced syncytia in the roots of *Arabidopsis thaliana* (Wieczorek et al., 2006), and AtEXPA11 (IbEXPA22/35) is likely to be involved in ethylene physiologies (Son et al., 2012). AtEXPA1 (IbEXPA12/13/25) is involved in the regulation of stomatal opening and salt and ABA stress (Gao et al., 2010; Wei et al., 2011; Zhang et al., 2011). Over-expression of *AtEXPB2* (*IbEXPB1/2/3/4/5/6/7/8/9*) could alleviate salt stress damage in transgenic tobacco plants (Chalekaei et al., 2021); AtEXLA2 (IbEXLA1/2) positively regulates the hypocotyl growth in *Arabidopsis thaliana* (Boron et al., 2014); and AtEXP2 (IbEXPA15) is involved in regulating gibberellin-mediated seed germination and increasing tolerance to salt and osmotic stress in *Arabidopsis* (Yan et al., 2014). The examination of interactions between IbEXP proteins indicates that IbEXP proteins tend to form complexes through protein interactions to perform crucial functions in the regulation of development and/or stress responses. This information will be conducive to further study of the significant roles of IbEXP proteins in stress response and plant development.

### 3.9 RNA-seq of sweetpotato during tuberous root development

**Figure 8:**
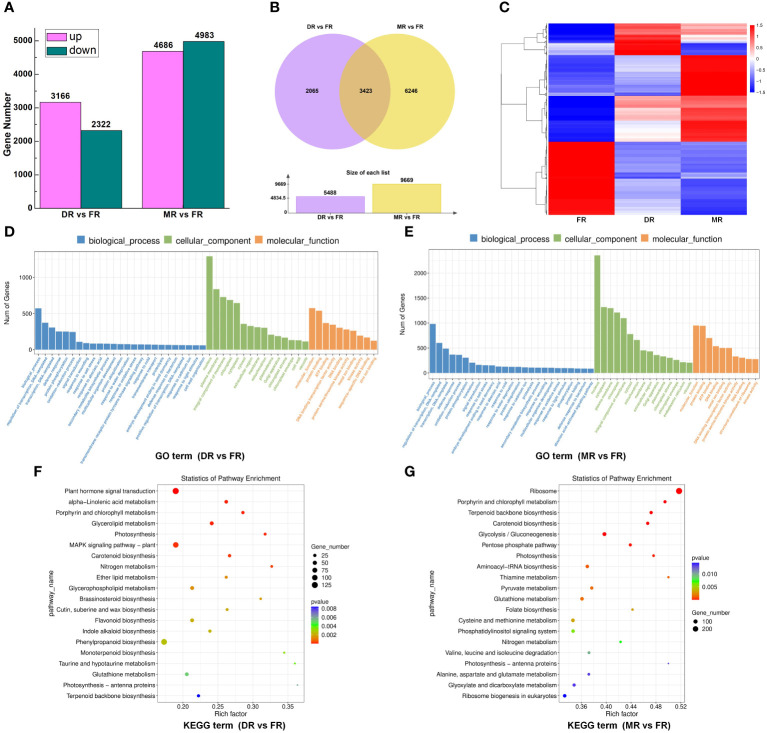
RNA-seq analysis of candidate genes that were involved in sweetpotato tuberous root development. **(A)**. Histogram visualizing the differentially expressed genes (DEGs) in groups DR vs. FR and MR vs. FR. The up-regulated DEGs are shown in red, and the down-regulated DEGs are shown in green. The y-axis represents the gene number. **(B)**. The Venn diagram represents the 3423 DEGs detected simultaneously in groups DR vs. FR and MR vs. FR. **(C)**. Hierarchical clustering analysis of 3423 DEGs detected simultaneously in the three developmental stages (FR, DR, and MR). The red and blue colors indicate the up-regulated and down-regulated DEGs in the heat maps, respectively. The scale bar denotes the value of log10 (FPKM+1); FPKM, fragments per kilobase of transcript sequence per million base pairs sequenced. **(D)**. Gene Ontology (GO) classification of DEGs that are enriched in cellular component (CC), biological process (BP), and molecular function (MF) in group DR vs. FR. **(E)**. Gene Ontology (GO) classification of DEGs that are enriched in cellular component (CC), biological process (BP), and molecular function (MF) in group MR vs. FR. **(F)**. KEGG pathway enrichment analysis of up- and downregulated DEGs that are primarily enriched in the regulatory pathway in groups DR vs. FR. **(G)**. KEGG pathway enrichment analysis of up- and downregulated DEGs that are primarily enriched in the regulatory pathway in groups MR vs. FR. “GeneRatio” indicates the ratio of the number of differential genes associated with one KEGG pathway to the total number of all DEGs.

Numerous members of various gene families have been reported to be involved in the regulation of sweetpotato tuberous root development. To explore the roles of members of different gene families during sweetpotato tuberous root development, we carried out RNA-seq of sweetpotato roots in different stages (FR, DR, and MR). More than 34 million sequence reads were obtained for each cDNA library, representing >5 Gb of sequence data for each sample. The RNA-seq data exhibited good correlations and were suitable to be used for further investigations (**Supplementary Figure S2**). A summary of the RNA-seq, assembly, annotation, and mapping is provided in **Supplementary Table S5–1**. Finally, a total of 45142 genes were identified from the three cDNA libraries using FPKM to estimate gene expression. Using p-value <0.05 and |log2 (fold change)| ≥1 as the significance threshold in DESeq2, 5488 genes, of which 3166 were up-regulated and 2322 were down-regulated, were identified as the differentially expressed genes (DEGs) between DR and FR, and 9669 genes, of which 4686 were up-regulated and 4983 were down-regulated, were identified as DEGs between MR and FR (**Figure 8**; **Supplementary Tables S5–2**, **S5–3**). In addition, 3423 DEGs were found simultaneously in both groups (DR vs. FR and MR vs. FR) (**Figure 8**; **Supplementary Table S5–4**), and these DEGs showed different expression patterns in these two groups (**Figure 8**). Most of the DEGs from different groups (DR vs. FR, MR vs. FR, DR vs. FR, and MR vs. FR) belonged to different gene families that are known to be involved in plant development, such as the MADS-box, AP2/ERF, DEAD, B3, bZIP, bHLH, NAC, Homeobox, Dof, WRKY, MYB, and AUX/IAA families (**Supplementary Tables S5–2**; **S5–3**; **S5–4**).

To explore the potential functions of these DEGs in sweetpotato tuberous root development, we performed functional enrichment using gene ontology (GO) analysis in two groups (DR vs. FR and MR vs. FR) to categorize the DEGs with respect to cellular component (CC), biological process (BP), and molecular function (MF). First, in the DR vs. FR group, for BP, the DEGs were principally enriched in biological processes, regulation of transcription, DNA-templated and transcription, DNA-templated; for CC, the majority of DEGs were associated with the nucleus, plasma membrane, and integral components of the membrane; and for MF, most of the DEGs were related to molecular function, protein binding, and ATP binding (**Figure 8**; **Supplementary Table S5–5**). Second, in the group MR vs. FR, for BP, the DEGs were principally enriched in biological processes, regulation of transcription, DNA-templated and transcription, DNA-templated; for CC, the majority of DEGs were associated with the nucleus, cytoplasm, and plasma membrane; and for MF, most of the DEGs were related to molecular function, protein binding, ATP binding, and DNA binding (**Figure 8**; **Supplementary Table S5–6**). In the DR vs. FR group, 131 pathways were screened using KEGG analysis, and we found that most of the DEGs were mainly enriched in multiple pathways associated with biosynthesis, metabolism, and plant hormone signal transduction (**Figure 8**; **Supplementary Table S5–7**). In the MR vs. FR group, 137 pathways were screened, and the majority of DEGs were enriched in multiple pathways in connection with biosynthesis and metabolism (**Figure 8**; **Supplementary Table S5–8**). These results indicate that the DEGs influence sweetpotato tuberous root development by regulating transcriptional levels of genes relating to signal transduction, cell component modification, biosynthesis, and regulation of transcription.

### 3.10 Transcriptome−wide identification of *IbEXP* genes during tuberous root development and their expression profiles in different tissues

**Figure 9:**
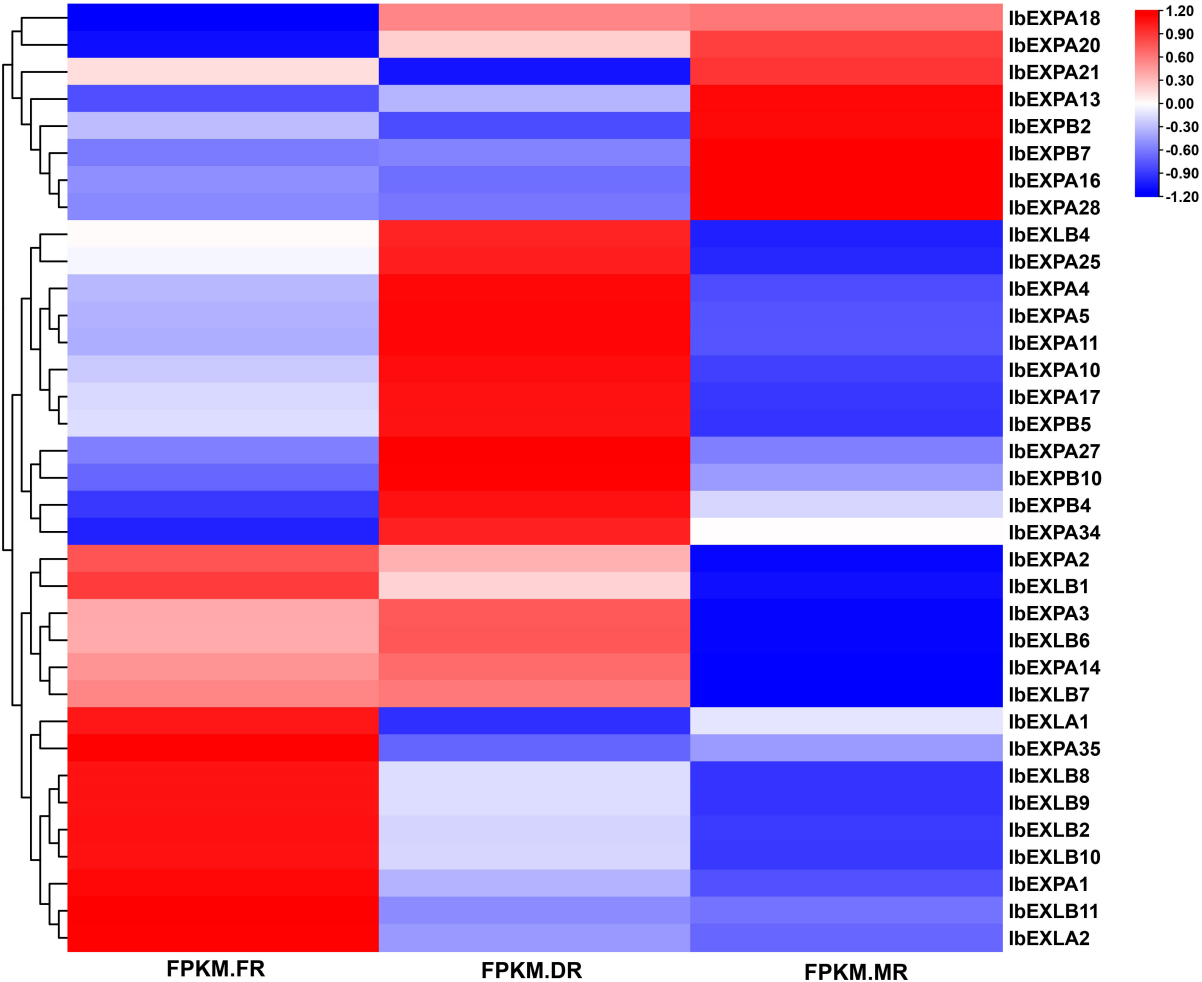
Heatmap of *IbEXPs* genes during sweetpotato tuberous root bulking. FPKM.FR, fibrous roots; FPKM.DR, developing tuberous roots; FPKM.MR, mature tuberous roots. FPKM, fragments per kilobase of transcript sequence per million base-pairs sequenced.

Accumulating evidence suggests that EXP genes have various critical roles in different developmental processes, such as root growth and architecture, fruit softening and ripening, seed production, and nodule formation and development. To investigate the potential biological roles of *IbEXP* genes in tuberous root formation and development, their transcript levels were explored at different developmental stages (FR, DR, and MR) based on our transcriptome data. We detected 35 *IbEXP* genes from all samples of these three periods and found that they displayed different expression patterns (**Figure 9**; **Supplementary Tables S5–9**); 13 and 10 *IbEXP* genes were significantly expressed in the DR vs. FR and MR vs. FR groups, respectively (**Supplementary Tables S5–9**).

**Figure 10:**
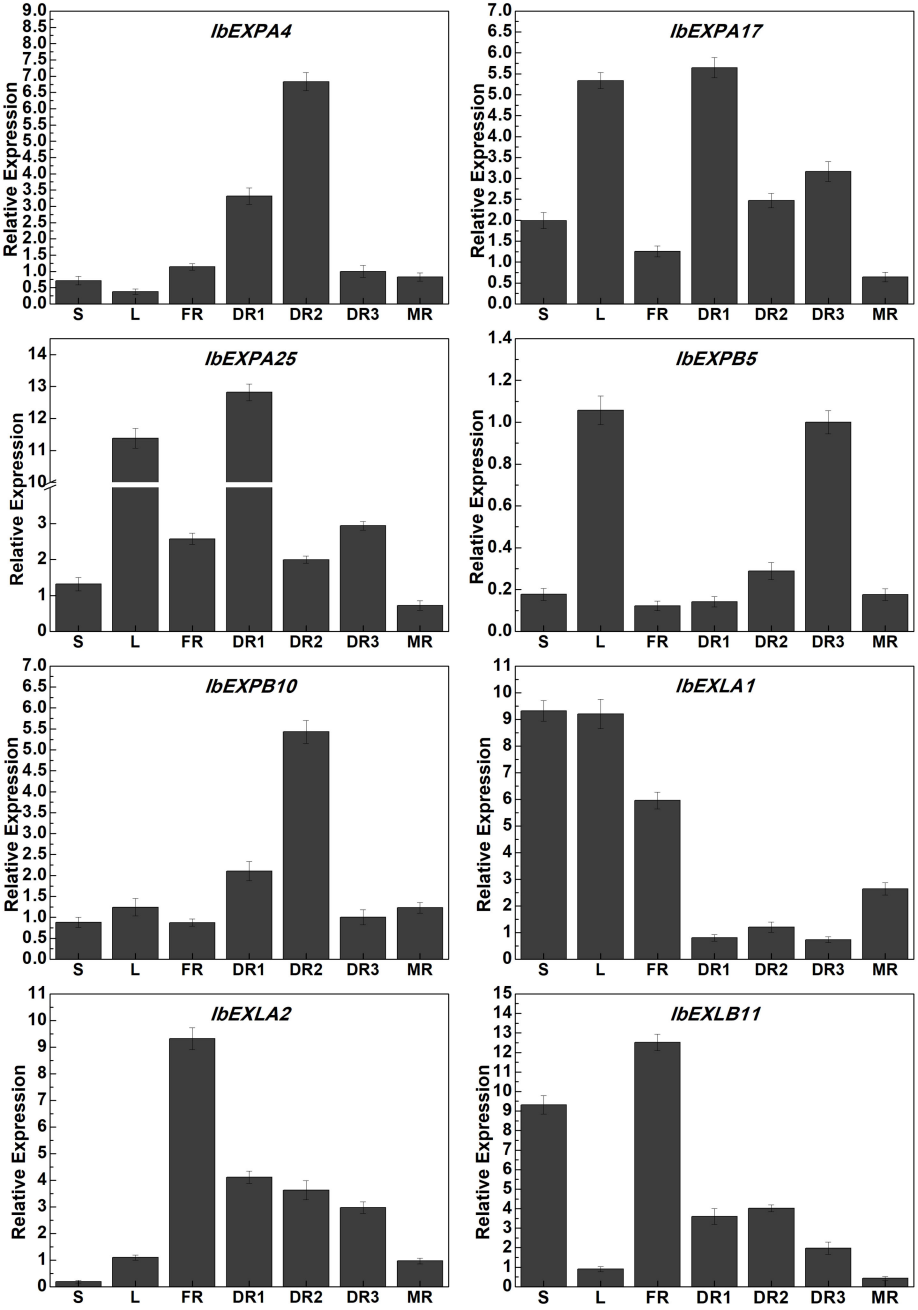
Expression profile analysis of *IbEXP* genes in different tissues by qRT-PCR. L, mature leaves at 60 dap; S, stems at 60 dap; DR1, tuberous roots at 30 dap; DR2, tuberous roots at 60 dap; DR3, tuberous roots at 100 dap; MR, mature tuberous roots at 120 dap.

To verify our transcriptome data, we performed qRT-PCR analysis to analyze the expression patterns of eight selected *IbEXP* genes from different subfamilies that showed distinct expression changes in our RNA-seq data in different periods in tuberous roots and other tissues. The transcript abundances of these *IbEXP* genes varied among different tissues (**Figure 10**). The transcripts of *IbEXPA4*, *IbEXPA17*, *IbEXPA25*, *IbEXPB5*, *IbEXPB10*, *IbEXLA2*, and *IbEXLB11* markedly accumulated in some developmental stages of tuberous roots (DR1, DR2, DR3, and MR), and their levels were significantly higher in one or two DR (DR1, DR2, and DR3) stages than in the MR stage. Moreover, *IbEXPA17*, *IbEXPA25*, *IbEXPB5*, and *IbEXLA1* also showed high expression levels in leaves. Transcripts of *IbEXLA1* were markedly accumulated in the FR stage and then dramatically reduced at DR1–3 before being induced by the development of tuberous roots. The expression of *IbEXLB11* was down-regulated with the development of tuberous roots and was significantly up-regulated in stems and FR. The expression of *IbEXPB5* was increased following tuberous root development; it showed the highest expression levels in DR3 and reduced levels in MR. The expression levels of *IbEXLA1* in stems and leaves were significantly higher than those in different developmental stages of tuberous roots. These results suggest that the selected EXP genes may have significant roles in different tissues and developmental stages of sweetpotato roots.

### 3.11 Expression patterns of *IbEXP* genes under multiple hormone treatments and abiotic stresses

**Figure 11:**
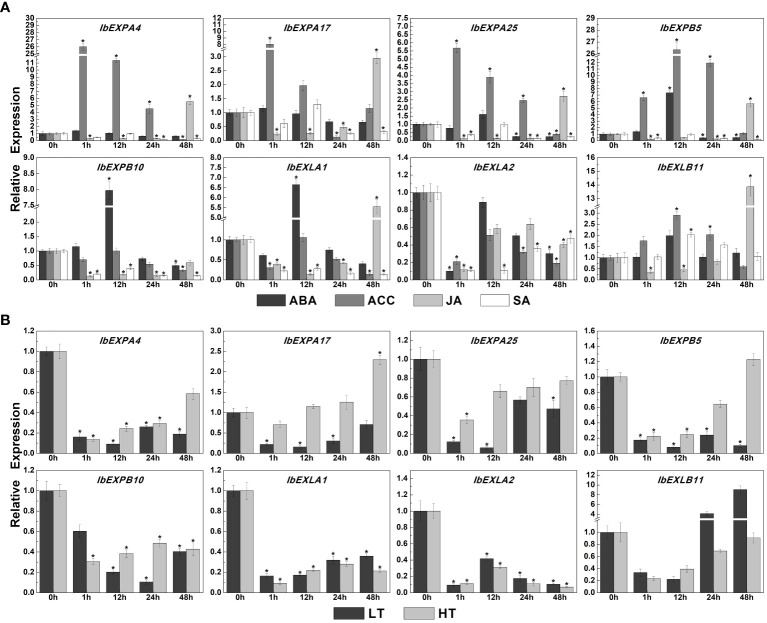
Relative expression levels of *IbEXP* genes detected by qRT-PCR under diverse hormone treatments and abiotic stresses. **(A)**. The expression levels of detected IbEXP genes under diverse hormone treatments. These hormone treatments include ABA (abscisic acid, 100 μM), ACC (1-aminocyclopropane-1-carboxylic acid, 100 μM), JA (jasmonic acid, 100 μM), and SA (salicylic acid, 2 mM). 0 h represents the WT seedlings that were not treated by each treatment. Bars indicate the mean of three biological replicates ± SE. The two-fold expression changes of IbEXP genes in each treated sample compared to the 0 h sample are considered to be significant expression changes. **(B)**. The expression levels of detected IbEXP genes under abiotic stresses. The abiotic stress treatments include low temperature (LT, 4˚C) and high temperature (HT, 42˚C). 0 h represents the WT seedlings that were not treated by each treatment. Bars indicate the mean of three biological replicates ± SE. The two-fold expression changes of IbEXP genes in each treated sample compared to the 0 h sample are considered to be significant expression changes. The asterisk indicate the significant differences of IbEXP gene expression changes in treated samples compared to the 0 h sample.

In addition to their crucial functions in plant development, EXPs have been confirmed to participate in responses to multiple abiotic stresses and exogenous plant hormones (Han et al., 2012; Zou et al., 2015; Yang et al., 2020). Thus, we investigated the transcript accumulation of eight *IbEXP* genes under various abiotic stresses and plant hormone treatments. The expression levels of these *IbEXP* genes were enhanced or decreased to varying degrees under different hormone treatments (ABA, ACC, JA, SA) (**Figure 11**). Specifically, the transcriptional levels of *IbEXPA4/17/25*, *IbEXPB5/10*, *IbEXLA1*, and *IbEXLB11* can be increased to various degrees by these four hormones, whereas *IbEXLA2* expression levels were reduced. Transcripts of *IbEXPB5/10* and *IbEXLA1* displayed high fold changes in expression (6.6-fold–7.9-fold increase compared with levels at 0 h after ABA treatment), whereas the other four *IbEXP* genes (*IbEXPA4/17/25*, *IbEXLB11*) exhibited changes with less than two folds. The expression levels of *IbEXPA4/17/25*, *IbEXPB5*, and *IbEXLB11* were up-regulated about 2.9-fold–26.3-fold compared with levels at 0 h under ACC treatment. After JA treatment, transcript levels of *IbEXPA17/25*, *IbEXPB5*, *IbEXLA1*, and *IbEXLB11* were increased by 2.9-fold–13.8-fold at certain time points. After SA treatment, only *IbEXLB11* displayed increased transcript abundance (two-fold change); transcript levels of the other seven EXP genes displayed decreases of varying degrees at all or some time points.

In consideration of the crucial functions of *IbEXP* genes in response to various abiotic stresses, as reported in previous studies, we also performed expression pattern analysis for the selected *IbEXP* genes under low temperature (LT, 4°C) and high temperature (HT, 42°C), according to our previous study (Meng et al., 2020). The results displayed that transcript levels of all eight selected *IbEXPs* were increased or reduced to various degrees (**Figure 11**). Those of *IbEXPA4/25*, *IbEXPB10*, *IbEXLA1*, and *IbEXLA2* were significantly reduced at all time points, whereas *IbEXPA17* showed marked reduction at all or some time points under low temperature stress, and its expression increased about 2.3-fold at 48 h. On the contrary, expression levels of *IbEXPB5* were dramatically reduced at all or some time points under high temperature stress but were observably increased about 4.1-fold–9-fold at 24 h and 48 h. These results suggest that many *IbEXP* genes function as crucial regulators in response to certain hormones (especially ABA, ACC, and JA), abiotic stresses, and/or signal transduction.

## 4 Discussion

Sweetpotato is a significant food crop with broad applications in industrial materials, animal feed, and human food. It has various natural advantages, including high stress resistance, high yield, and wide adaptability (Liu, 2017). Plant growth is determined by cell enlargement and proliferation and is restricted by the cell wall, which limits increases in the protoplasm of plant cells. The mechanism of cell wall extension has long been a focus of investigation owing to the crucial functions of cell wall enlargement during plant morphogenesis (Lin et al., 2011). EXPs are necessary for plant development and play critical roles in the relaxation of the plant cell wall. They are thus among the most frequently investigated structural proteins. EXPs promote the growth of the primary cell wall in plants. They can loosen the cell wall via the cleavage of hydrogen bonds between hemicellulose and cellulose microfibrils, fill microfibrils, and combine cellulose networks to form reticular systems (Cho and Cosgrove, 2000; Li et al., 2003), resulting in increases in cell wall strength and toughness and continuous extension of the cell wall (Ding et al., 2016). However, members of the EXP gene family in *I. batatas* had not been systematically and comprehensively characterized. The completed genome sequencing of sweetpotato and the availability of advanced bioinformatics tools provide an excellent foundation for the identification and characterization of specific gene families (Yang et al., 2017). In this study, the *IbEXP* genes in *I. batatas* were systematically characterized and analyzed, and their multiple molecular characteristics were further explored. Our study provides a foundation for further investigations of the regulatory modes and molecular functions of *IbEXP* genes in sweetpotato growth and development and stress tolerance.

### 4.1 Characterization of IbEXPs in sweetpotato

In this study, 59 *IbEXP* genes were identified from the *I. batatas* genome, whereas only 37 *ItfEXP* genes were identified in *I. trifida*, suggesting that number expansion of the EXP gene family has occurred in *Ipomoea batatas* compared with its diploid wild relative (Li et al., 2022). These 59 *IbEXP* genes were divided into four subfamilies: *IbEXPA*, *IbEXPB*, *IbEXLA*, and *IbEXLB*, which were similar to those in other plant species. The number of 59 *IbEXP* genes is more than 46, 38, 36, 46, 36, and 38 EXP genes in barley (Liu et al., 2021a), *Brachypodium distachyon* (Chen et al., 2020b), *Arabidopsis* (Sampedro and Cosgrove, 2005), gingkgo (Guo et al., 2023), potato (Chen et al., 2019), and tomato (Lu et al., 2016), and is similar to 52 and 58 EXP genes in tobacco (Ding et al., 2016) and rice (Sampedro and Cosgrove, 2005), while is obviously less than 75, 88, 92, 93, and 241 EXP genes in soybean (Zhu et al., 2014), maize (Zhang et al., 2014), sugarcane (Santiago et al., 2018), cotton (Lv et al., 2020), and common wheat (Han et al., 2019). These differing numbers suggest that the size of the EXP gene family has changed significantly in different plants during evolution. Likewise, the sizes of EXP subfamilies are unevenly distributed in different plant species, although the EXPA subfamily occupies the highest proportion compared with the other three subfamilies in all plants (**Table 2**). In addition, the 59 *IbEXP* genes in sweetpotato were disproportionately distributed on 14 chromosomes. Similar uneven distributions have been observed in wheat (Han et al., 2019), cotton (Lv et al., 2020), barley (Liu et al., 2021a), and potato (Chen et al., 2019) et al., indicating that the number of *IbEXP* genes on each chromosome is unrelated to the size of the chromosome. Therefore, *IbEXP* genes on chromosomes were clustered instead of being evenly distributed, possibly owing to uneven gene replications of chromosome fragments.

**Table 2:** Summary of each expansin subfamily in 15 plant species.

Species	EXPA	EXPB	EXLA	EXLB	Total	Reference
*Ipomoea batatas*	36 (61%)	10 (17%)	2 (3.4%)	11 (18.6%)	59	In this study
*Ipomoea trifida*	23 (62.2%)	4 (10.8%)	2 (5.4%)	8 (21.6%)	37	(Li et al., 2022)
*Saccharum officinarum*	51 (55.4%)	38 (41.3%)	3 (3.3%)	0 (0%)	92	(Santiago et al., 2018)
*Oryza sativa*	34 (58.6%)	19 (32.8%)	4 (6.9%)	1 (1.7%)	58	(Sampedro and Cosgrove, 2005)
*Triticum aestivum*	121 (50.2%)	104 (43.2%)	16 (6.6%)	0 (0%)	241	(Han et al., 2019)
*Zea mays*	36 (40.9%)	48 (54.5%)	4 (4.5%)	0 (0%)	88	(Zhang et al., 2014)
*Hordeum vulgare*	24 (52.2%)	16 (34.8%)	6 (13%)	0 (0%)	46	(Liu et al., 2021a)
*Brachypodium distachyon*	30 (79%)	4 (10.5%)	3 (7.9%)	1 (2.6%)	38	(Chen et al., 2020b)
*Arabidopsis*	26 (72.2%)	6 (16.7%)	3 (8.3%)	1 (2.8%)	36	(Sampedro and Cosgrove, 2005)
*Glycine max*	49 (65.3%)	9 (12%)	2 (2.7%)	15 (20%)	75	(Zhu et al., 2014)
*Ginkgo biloba*	32 (69.5%)	4 (8.7%)	5 (10.9%)	5 (10.9)	46	(Guo et al., 2023)
*Gossypium spp*	67 (72%)	8 (8.6%)	6 (6.5%)	12 (12.9%)	93	(Lv et al., 2020)
*Nicotiana tabacum*	36 (69.2%)	6 (11.5%)	3 (5.8%)	7 (13.5%)	52	(Ding et al., 2016)
*Solanum tuberosum*	24 (66.6%)	5 (13.9%)	1 (2.8%)	6 (16.7%)	36	(Chen et al., 2019)
*Solanum lycopersicum*	25 (65.8%)	8 (21.1%)	1 (2.6)	4 (10.5%)	38	(Lu et al., 2016)

Although IbEXP protein properties displayed significant differences, IbEXPs in the same clade harbored relatively conserved gene structures, motifs, and domains, which could provide an important reference for phylogenetic analysis and functional investigation. These results confirm that genes originating from a single ancestor can gradually expand and evolve (Lv et al., 2020). IbEXP members of the same subfamily tended to display similar compositions with respect to motifs, domains, and exon/intron structure, which were similar to those in other plant species such as tobacco, soybean, and cotton (Zhu et al., 2014; Ding et al., 2016; Lv et al., 2020). Gene structure analysis exhibited that about 83.1% of *IbEXP* genes harbored 1–5 exons, which displayed similarity with those of EXP genes in maize (Zhang et al., 2014), soybean (Zhu et al., 2014), tea (Bordoloi et al., 2021), *Populus* (Yin et al., 2023), and banana (Backiyarani et al., 2022). However, several *IbEXP* genes had more than eight exons, indicating the high divergence among them. The N-terminal conserved motifs (DPPB domains) of IbEXP proteins, which are rich in Cys residues with a characteristic catalytic domain, may be related to disulfide bond formation (Jin et al., 2020). The C-terminal motif (pollen_allerg_1 domain), which contains conserved aromatic amino acids and polar tryptophan residues on its surface, was considered to be the polysaccharide binding domain. The existence of these motifs in IbEXP proteins suggests that they are crucial for the functions of IbEXP proteins in cell wall enlargement and loosening. Although the conserved DPBB and pollen_allerg_1 motifs were similar among most IbEXPs in the same subfamily, there were significant differences in molecular characteristics, which may be generated by sequence differences in non-conserved regions. Some specific motifs were only found in certain subfamilies, suggesting that some *IbEXP* genes may play specific roles in plant development, given that previous studies have reported that EXPs perform functions in various biological processes (Zou et al., 2015; Che et al., 2016; Brasileiro et al., 2021; Zhang et al., 2021a; Chen et al., 2022).

### 4.2 Evolutionary relationships and collinearity analysis of IbEXPs in sweetpotato

Phylogenetic relationship analysis showed that the 59 IbEXPs could be grouped into four subfamilies based on sequence homologies and subfamily classifications of *Arabidopsis* AtEXPs and rice OsEXPs (Sampedro and Cosgrove, 2005; Choi et al., 2006; Zhang et al., 2014). At least one IbEXP protein was found in each subfamily of rice and *Arabidopsis* (Sampedro and Cosgrove, 2005; Choi et al., 2006), indicating that the discrepancies of EXP proteins may occur earlier than dicots and monocots. The IbEXP proteins in sweetpotato were unevenly distributed among the four subfamilies; most of them were members of the EXPA and EXPB subfamilies, whereas only a few belonged to the EXLA and EXLB subfamilies. Similar uneven distributions of members across these four subfamilies are also found in other plants, such as sugarcane (Santiago et al., 2018), rice (Sampedro and Cosgrove, 2005), *Arabidopsis* (Sampedro and Cosgrove, 2005), cotton (Lv et al., 2020), potato (Chen et al., 2019), and tomato (Lu et al., 2016). These results indicate that members of the EXPA and EXPB subfamilies may have more crucial roles in plant growth and development. For instance, the rice *OsEXPA10*, an Al‐inducible EXP gene in rice, plays significant roles in cell elongation of roots (Che et al., 2016). Overexpression of *GhEXPA8* in cotton improves the length of fibers and micronaire value (Bajwa et al., 2015). *TaEXPA2* has crucial functions in seed production and response to multiple abiotic stresses (salt, drought, oxidative, and Cd) (Chen et al., 2016, 2017; Ren et al., 2018; Chen et al., 2018b; Yang et al., 2020). The β-expansin gene *OsEXPB2* participates in the architecture of the root system in rice (Zou et al., 2015). Moreover, the proportion of members in each subfamily varies greatly among different plants. The classification of IbEXP proteins displayed both similarities and discrepancies compared with that of other plants, suggesting a diversity of functions and structures of EXP proteins exist in different plants.

Gene duplications are vital driving forces during the processes of expansion and evolution of many gene families in plants and can promote the generation of novel functional genes and species, enabling plants to better resist adverse environmental conditions (Lynch and Conery, 2000; Cannon et al., 2004; Li et al., 2021). Previous investigations in moso bamboo (Jin et al., 2020), maize (Zhang et al., 2014), barley (Liu et al., 2021a), potato (Chen et al., 2019), banana (Backiyarani et al., 2022), and *Brassica* species (Li et al., 2021) suggested that duplication events of tandem, segments, and genome may explain the expansion and evolution of *IbEXP* genes in sweetpotato. Analogously, collinearity analysis showed that multiple *IbEXP* genes had duplications of tandems and segments, suggesting that some *IbEXP* genes may be generated via gene duplications; this further supports a mechanism that brings about the expansion of *IbEXP* genes. The segmental and tandem duplications made similar contributions to the increase in *IbEXP* genes. In addition, the *IbEXP* genes exhibiting segmental duplications and tandem repeats were usually from the same subfamily, and the *IbEXP* gene pairs were mainly from the EXPA, EXPB, and EXLB subfamilies, indicating that expansions of *IbEXP* genes in specific subfamilies may be beneficial for sweetpotato growth and development and for adaptation to changing environmental conditions. These results are similar to those reported for EXP genes in moso bamboo (Jin et al., 2020), maize (Zhang et al., 2014), potato (Chen et al., 2019), and so on, indicating the critical evolutionary functions of segmental and tandem duplications in gene expansions.

Furthermore, synteny analysis was used to estimate the relationships between *IbEXP* genes and their counterparts in nine plants studied, comprising *Ipomoea triloba*, *Oryza sativa*, *Arabidopsis*, *Zea mays*, *Triticum aestivum*, *Capsicum annuum*, *Solanum lycopersicum*, *Brassica oleracea*, and *Brassica rapa*. The largest numbers of orthologous genes were identified between sweetpotato and *Ipomoea triloba*, which further proved their close evolutionary relationship; the next most closely related plants, based on orthologous gene numbers, were *Solanum lycopersicum*, *Arabidopsis*, *Capsicum annuum*, *Brassica oleracea*, and *Brassica rapa*. These orthologous gene pairs may be derived from a common ancestor of sweetpotato and other plant species. In addition, more complex relations such as multiple *Ipomoea trilobato*-single sweetpotato genes or multiple sweetpotato-single *Ipomoea trilobato* genes were found, indicating that the orthologous genes might have vital functions in the evolution of *IbEXP* genes in sweetpotato. However, there were no orthologous genes between sweetpotato and three Gramineae plants (*Oryza sativa*, *Zea mays*, and *Triticum aestivum*), and some EXP subfamilies such as EXLB were lost in many plants (Zhang et al., 2014; Santiago et al., 2018; Han et al., 2019; Liu et al., 2021a), probably owing to the abundant chromosomal fusions and rearrangements taking place in their genomes, and this selective gene loss has seriously hindered the identification of synteny relations (Paterson et al., 2012). These findings may be related to the phylogenetic relationships between sweetpotato and these nine plants. Large-scale duplications predate plant divergence and have vital functions in the expansion of the *IbEXP* gene family.

### 4.3 Expression patterns and functional prediction of *IbEXPs* in sweetpotato

The EXP gene family has received increasing attention owing to the widespread participation of its members in various biological processes of plant development and responses to diverse stresses. Numerous studies have shown that EXP genes are excellent candidates for regulation of growth and development and for improving the stress tolerance of crops via molecular breeding (Choi et al., 2006; Han et al., 2012; Li et al., 2015). For example, *OsEXPA8*, *OsEXPA10*, and *OsEXPB2* in rice (Wang et al., 2014; Zou et al., 2015; Che et al., 2016), *GmEXPB2*, *GmEXLB1*, and *GmINS1* in soybean (Guo et al., 2011; Li et al., 2015; Kong et al., 2019; Yang et al., 2021), and *TaEXPA2*, *TaEXPA8*, *TaEXPB23* in wheat (Han et al., 2012; Chen et al., 2017; Chen et al., 2018b; Peng et al., 2019; Yang et al., 2020) have been proven to have crucial functions in plant development and/or responses to various adverse environmental conditions. However, the functions of sweetpotato *IbEXP* genes in the regulation of stress resistance and plant development have remained poorly understood. The expression profiles of genes are closely related to their biological functions, and the identification of gene expression can be conducive to the characterization of gene functions. In this study, our RNA-seq and qRT-PCR results showed that more than half of the *IbEXP* genes were involved in sweetpotato tuberous root development. The *IbEXP* genes exhibited markedly differential expression in different tissues and after different treatments with hormones and abiotic stresses, suggesting diverse and critical functions of the *IbEXP* genes in plant development and stress responses. For instance, the expression levels of multiple *IbEXP* genes, particularly *IbEXPA4*, *IbEXPA25*, *IbEXPB10*, *IbEXLA2*, and *IbEXLB11*, showed differential expression in different tissues, and *IbEXPA4, IbEXPB5*, and *IbEXLB11* were dramatically induced or suppressed under different hormone and abiotic stresses, indicating that they may have significant functions in sweetpotato growth and development and stress/hormone response and/or help plants to reduce the damage caused by various stresses.

The potential functions of *IbEXP* genes in growth and development, stress, and hormone responses were further supported by cis-element and evolutionary relationship analysis. EXP genes in similar subfamilies may be derived from the same gene/fragment duplication events, and functional investigations of EXPs have verified conserved and/or similar roles in the same subfamily of plants (Choi et al., 2006, 2008; Wang et al., 2014; Li et al., 2015; Che et al., 2016; Yang et al., 2021). Previous studies have shown that *TaEXPA2* (Chen et al., 2017; Ren et al., 2018; Chen et al., 2018b; Yang et al., 2020), *OsEXPA8* (Wang et al., 2014), *OsEXPA10* (Che et al., 2016), *TaEXPA8* (Peng et al., 2019), and *AtEXPA1* (Gao et al., 2010; Zhang et al., 2011) in the EXPA subfamily; *AtEXPB1* (Kwon et al., 2008), *GmINS1* (Yang et al., 2021), *OsEXPB2* (Zou et al., 2015), *GmEXPB2* (Guo et al., 2011; Li et al., 2015), and *TaEXPB23* (Han et al., 2012) in the EXPB subfamily; *AtEXLA2* (Abuqamar et al., 2013; Boron et al., 2014) in the EXLA subfamily; *GmEXLB1* (Kong et al., 2019), *MdEXLB1* (Chen et al., 2022), and *BrEXLB1* (Muthusamy et al., 2020) in the EXLB subfamily are crucial participants in tissue growth and development and/or various stress responses. Here, phylogenetic analysis showed that the *IbEXPA1/-25/-26* genes were closely linked to *AtEXPA1/-10/-15*, the *IbEXPB10* gene was closely linked to *AtEXPB1/-3* and *OsEXPB16/-17*, and the *IbEXLA1/2* genes were closely linked to *AtEXLA1/-2/-3*. Expression levers of eight genes (*IbEXPA4/-17/25*, *IbEXPB5/-10*, *IbEXLA1/-2*, and *IbEXLB11*) displayed tissue specificity in different tissues, and their transcripts were observably induced or suppressed under various hormone and stress treatments, implying that they might have vital roles in regulation of the pathways of plant development and stress responses. Furthermore, IbEXP members in the same phylogenetic branch exhibited both similar and discrepant expression profiles, suggesting the diversity of their potential functions. Therefore, we speculated that development-related and/or stress-response-related IbEXPs that were grouped in the same subfamilies would participate in the control of plant development and/or stress/hormone responses.

Previous investigations have shown that plant hormones play significant roles in controlling plant development and adaptation to various adverse environmental conditions (Verma et al., 2016). Moreover, the cis-elements located in gene promoter regions function as crucial regulators of gene expression. In this study, numerous cis-elements were detected in promoters of *IbEXP* genes and shown to be related to hormones, growth and development, and stresses; these are ABRE, ARE, ERE, TGACG-motif, CCGTCC-box, TGA-element, P-box, as-1, CAT-box, LTR, MSA-like, and TATC-box. The results suggest that these elements could function as necessary participants in the regulation of growth and development and hormone and/or stress signaling. In addition, the expression levels of the examined *IbEXP* genes (*IbEXPA4/-17/-25*, *IbEXPB5/-10*, *IbEXLA1/-2*, and *IbEXLB11*) were markedly increased or reduced by one or some hormones and abiotic stresses. These results are similar to those of previous investigations in moso bamboo (Jin et al., 2020), *Ipomoea trifida* (Li et al., 2022), tobacco (Ding et al., 2016), *Brachypodium distachyon* (Chen et al., 2020b), and banana (Backiyarani et al., 2022). However, the exact biological functions of the sweetpotato *IbEXP* genes remain to be determined. An investigation of the involvement of these *IbEXP* genes in sweetpotato development and stress regulation could provide valuable information regarding their potential functions in growth, development, and stress tolerance.

## 5 Conclusion

Collectively, in the present study, 59 *IbEXP* genes from the sweetpotato genome were systematically identified and characterized and found to be unevenly distributed on 14 of 15 chromosomes. Their phylogenetic classification, conserved motifs and domains, collinearity relationships, gene structure, cis-elements, molecular characteristics, and chromosome localization were systematically investigated. Duplication events such as tandem and segmental duplications were found to have been conducive to the expansion of the sweetpotato IbEXP gene family, and synteny analysis of orthologous genes between sweetpotato and typical plant species provided significant insights into the phylogenetic characteristics of IbEXP genes in sweetpotato. Moreover, the results of RNA-seq and qRT-PCR analyses revealed various differential expression patterns of IbEXP genes in different developmental tissues and following different stress and hormone treatments. Multiple tissue-specific expression and hormone- or stress-induced IbEXP genes may have very close relationships with the transcriptional regulation of sweetpotato development and stress responses. In conclusion, the present results both contribute to our understanding of the complexity and importance of the EXP gene family and lay the foundation for future comprehensive analysis of the potential functions of this family in regulating plant growth, development, and stress tolerance.

## Data availability statement

The datasets presented in this study can be found in online repositories. The names of the repository/repositories and accession number(s) can be found below: http://www.ncbi.nlm.nih.gov/Traces/sra, GSM7838330 http://www.ncbi.nlm.nih.gov/Traces/sra, GSM7838331 http://www.ncbi.nlm.nih.gov/Traces/sra, GSM7838332.

## Author contributions

JZ: Conceptualization, Data curation, Funding acquisition, Software, Writing – original draft. TD: Supervision, Validation, Writing – review & editing. MZ: Data curation, Software, Validation, Writing – review & editing. DD: Data curation, Writing – review & editing, Funding acquisition. RL: Writing – review & editing, Data curation. QY: Writing – review & editing, Funding acquisition, Data curation. YS: Funding acquisition, Writing – review & editing. ZZ: Project administration, Conceptualization, Funding acquisition, Writing – review & editing.
