# Supplementary material for: Transcriptome- and genome-wide systematic identification of expansin gene family and their expression in tuberous root development and stress responses in sweetpotato (Ipomoea batatas)
Source: Front Plant Sci. 2024 Jun 20;15:1412540. doi: 10.3389/fpls.2024.1412540 (PMC11223104; doi:10.3389/fpls.2024.1412540)
Supplement: Supplementary file 1 [file DataSheet_1.zip › Data Sheet 1/Supplementary files/Supplementary Figures.docx]

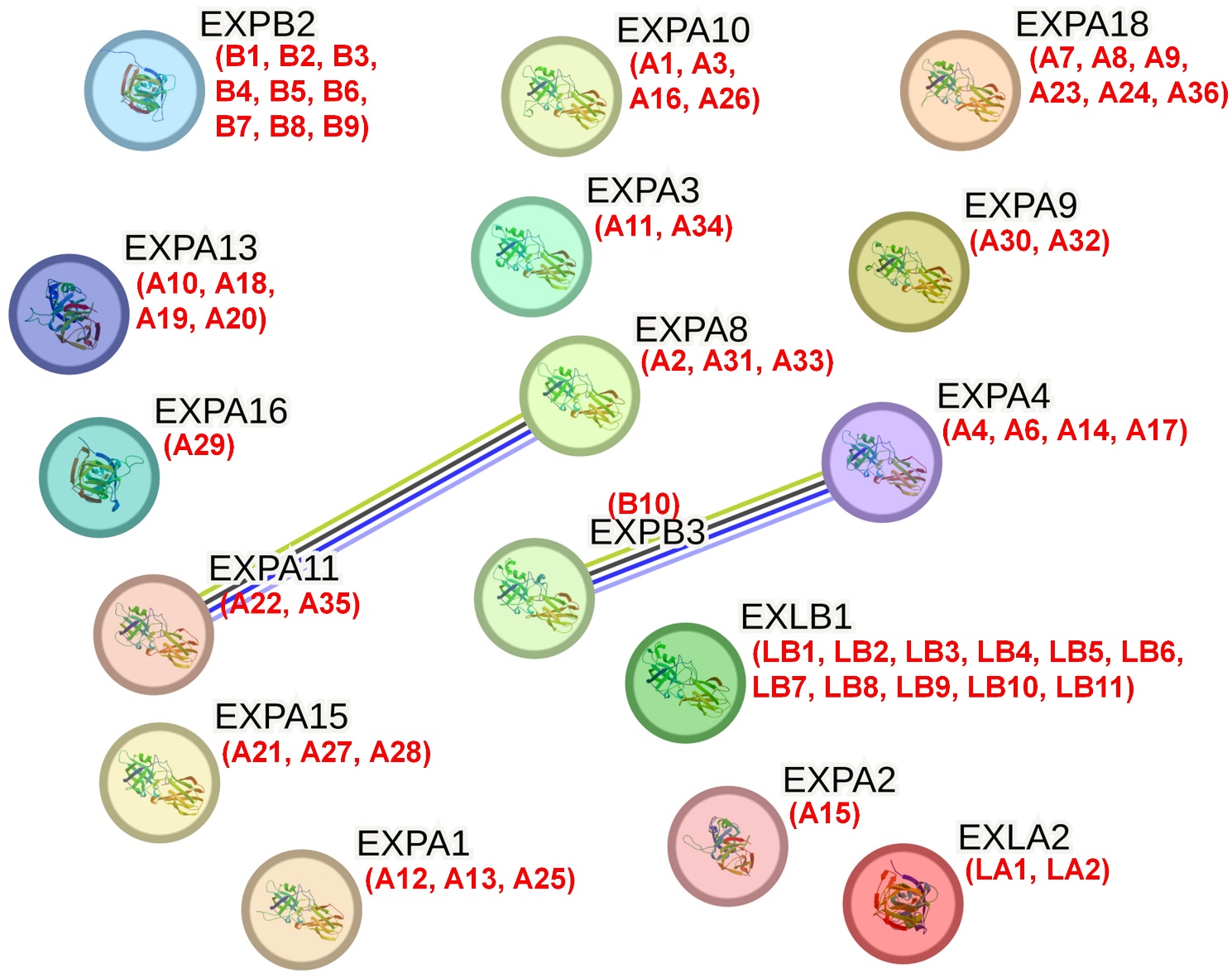


**Figure S1 Interaction networks of** **IbEXPs in sweetpotato according to the orthologues in *Arabidopsis*.**

The amino acid sequences of IbEXPs in sweetpotato were employed to search the STRING database according to the orthologues in *Arabidopsis*. Network node represents proteins, and edge represents protein-protein associations. The different colored lines between the nodes indicate the different kinds of interactions. The numbers (IbEXPs gene name) in brackets represent the corresponding orthologues in sweetpotato. The filled and empty nodes delineate the proteins with known or predicted 3D structures and unknown 3D structures, respectively.


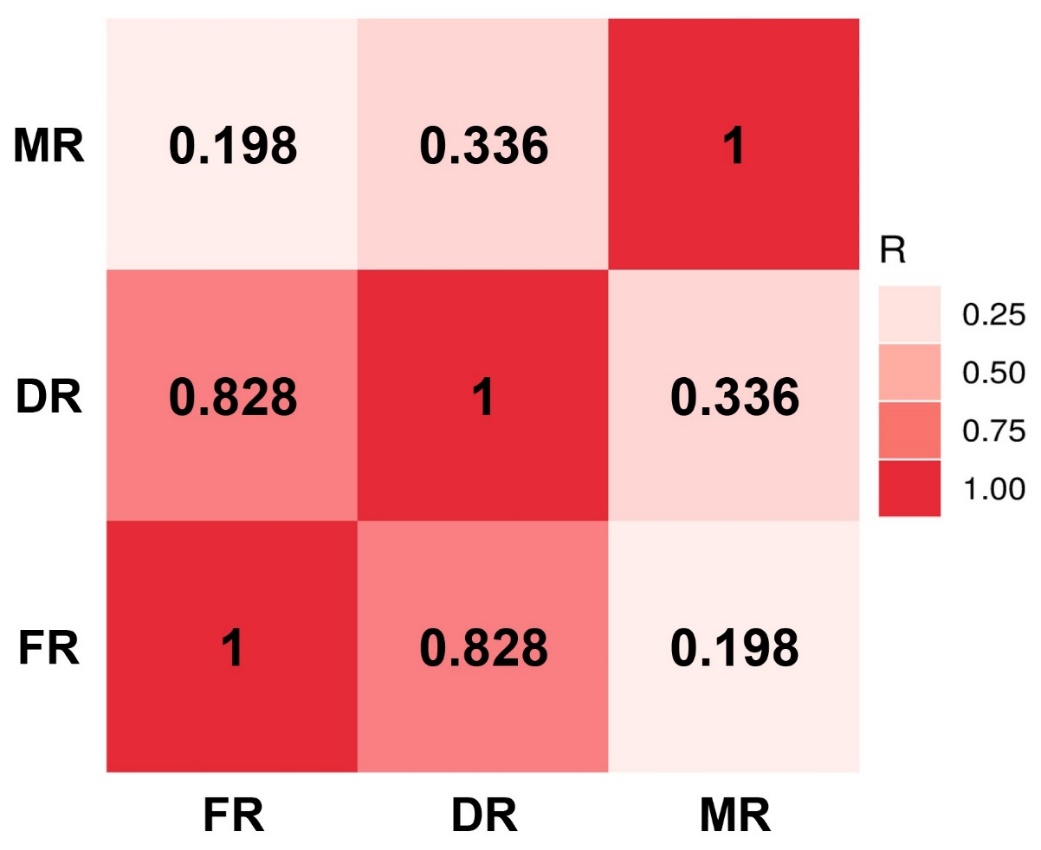


**Figure S2 Correlation analysis (through Pearson’s correlation coefficient) of RNA-seq data among FR, DR, MR.**

FR, fibrous roots; DR, developing tuberous roots; MR, mature tuberous roots.
