# Supplementary material for: Transcriptome- and genome-wide systematic identification of expansin gene family and their expression in tuberous root development and stress responses in sweetpotato (Ipomoea batatas)
Source: Front Plant Sci. 2024 Jun 20;15:1412540. doi: 10.3389/fpls.2024.1412540 (PMC11223104; doi:10.3389/fpls.2024.1412540)
Supplement: Supplementary file 1 [file DataSheet_1.zip › Data Sheet 1/Supplementary files/Supplementary file 1 The amino acid sequences of EXPs proteins in sweetpotato, Arabidopsis and rice.docx]

**Supplementary file 1. The amino acid sequences of EXPs proteins in sweetpotato, *Arabidopsis* and rice**

>IbEXPA1

MAVLELLLVGVLATLSPVHGYWGWSSARATFYGGGDASGTMGGACGYGNLYSSGYGTNTAALSTALFNNGLSCGSCFQIRCVNDRSCLRGVITVTATNFCPPGGWCEPPNPHFDLSQPVFLRIAQYRAGVVPVAYRRVPCRRSGGIRFTINGHAFFNLVLVTNVGGSGDVHAVYIKGSRTGWQMMSRNWGQNWQSNANLNGQSLSFRVVTGDGRSVVSYNAAPPGWSFGQTYSGAQFR

>IbEXPA2

MAVPTPFSIPFLLLFLPSFCVHLAFGDYGGWQTAHATFYGGSDASGTMGGACGYGNLYRTGYGTNIAALSTALFNNGLACGSCYELMCTGPYCLPGSVTITATDFCPPNPGLPNDNGGWCNPPRPHFDMAEPAYLQIGVYRAGIVPVNYRRVPCRKKGGIRFTINGHIFFNLVLVTNVGGFGDVHAVAIKGSRTGWQQMSRNWGQNWQSNSVLNGQSLSFQVTTGDGRTVTSFNAAPSDWQFGQTYEGAQF

>IbEXPA3

MAMAAALSVSFSLLFFSSFCFHLSFADYGGWQSAHATFYGGGDASGTMGGACGYGNLYSTGYGTNIAALSTALFNNGLACGSCYELMCNGPYCVPGSITVTATDFCPPNPGLSNDNGGWCNSPRQHFDMAQPAYLQIAIYRAGIVPVSYRKVPCRKKGGIRFTINGHTYFNLVLVTNVGGSGDVHSVAIKGSKTGWQQMSRNWGQNWQSNSVLDGQSLSFQVTTGDGRTVTSYNAAPANWRFGQTFEGAQF

>IbEXPA4

MGFLSVVCISFLVLVLSLSVEVEGRIPGVYSGGSWENAHATFYGGSDASGTMGGACGYGNLYSQGYGVNNGALSTALFNNGLSCGACFEIKCDKDKSCYPGSPSIFITATNFCPPNYALPNDNGGWCNPPRSHFDLAMPMFLKIAEYRAGIVPVVYRRVPCKKKGGIRFTINGHRYFNLILITNVGGAGDIVRASVKGSKTGWMDLSRNWGQNWQTNAELMGQSLSFRVRGSDRRSSTSWNIAPSNWQFGQTFVGKNFRV

>IbEXPA5

MEAAAMAFSYAKLTLLAIFLLRLQSTAVADYGGWQSAHATFYGGGDASGTMDIDLLTCWAKTNKRPRPSLVVRKLFGERERSPTPPPGYGITGSFKHLIIMLLLPGGACGYGNLYSQGYGTQTAALSTALFNNGLSCGECFELRCDGDSRSCLPGTITVTATNFCPPNYALPNNDGGWCNPPLQHFDMAQPAFLHIAKYRSGIVPVVYRRVPCVRKGGIRFTINGHAFFNLVLVTNVAGPGNVYQVSIKGSKTGWQTMSRNWGQNWQSNSNFNGQSLSFRVTAGDGRTLTSYNVAPTNWQFGQTFEGAQF

>IbEXPA6

MGVFSAILIISVVSSLTITGEARVPGVYSGGPWQSAHATFYGGSDASGTMGGACGYGNLYSQGYGVNTAALSTALFNDGLSCGACFEIKCANDKSCFSGSPSIFITATNFCPPNYALPNDNGGWCNPPRPHFDLSMPMFLTIAQYRAGIVPVVHRRVACKKRGGIRFTINGHRFFNLVLISNVAGAGDIEKVSVKGSNTGWIGMSRNWGQNWETSATLVGQSLSFKVKGSDRRTSTSWNMVPANWQFGQTYVGRNFHV

>IbEXPA7

MASFLRRLSFAFFCVLAVIGKATAAGYYSHPVFHTGAWKLAHATFYGDESASETMGAARLRLPPTGKRFASLKAHQSVYSGRAGSEPNLGPTWLNLVVNLTNSCRRGACGYGNLFNNGYGTATAALSTVLFNNGFACGQCFQIRCAQSPYCYKGFPTTTVTATNLCPPNWAEDSNNGGWCNPPRTHFDMSKPAFMKIAQWKAGIVPVMYRRVDNQSESSASFPYHGYHALGVVVSDSTYKGMGTGCWCYVMNEGGDIAAVWVKGSRTGWIRMSHNWGASYQAFGTLGGQSLSFKLTSYSSHETIVAYNVVPSNWNVGLTYQAKVNFH

>IbEXPA8

MAFRRLSLLSFSLLAVIGKAMAAGYYSHPVFHTGAWKLAHATFTDLICPETMGAISKCYLDCSTLLQKSPPAPCRPERGDCSSALWWSAGNGYGTATAALSTVLFNNGFACGQCFQIRCAQSPYCYKGFPTTTVTATNLCPPNWAEDSNNGGWCNPPRTHFDMSKPAFMKIAQWKAGIVPVMYRRKWVLAVGVRDERGGGDIAAVWVKGSRTGWIRMSHNWGASYQAFGTLGRPIPSFKLTSYSSHETIVAYNVVPSNWNVGLTYQAKVNFH

>IbEXPA9

MASFLRRLSFAFFSLLAVIGKAMAAGYYSHPVFHTGAWKLAHATFYGDESASETMGGACGYGNLFSNGYGTATAALSTVLFNNGFACGQCFQIRCAQSPYCYKGFPTTTVTATNLCPPNWAEDSNNGGWCNPPRTHFDMSKPAFMKIAQWKAGIVPVMYRRVPCIRSGGVRFNLQGNGYWLLVYVMNVGGGGDIAAVWVKGSRTGWIRMSHNWGASYQAFGTLGGQSLSFKLTSYSSHETIVAYNVVPSNWNVGLTYQAKVNFH

>IbEXPA10

MPATIPHVLAVILLCYITPLAHSHYHWPSSSAPAQSEWRPARATYYAPADPRDAVGGACGFGDLERSGYGKATAGLSTVLFERGQICGACFEVRCVEDLRWCIPGTSIIVTATNFCAPNYGFEPDGGGHCNPPNAHFVLPIEAFEKIAIWKASNMPIQYRRIKCRKEGGVRFTITGAGISLSVLISNVAGAGDIVAVKIKGTRTGWLPMGRNWGQIWHINADLKNQPLSFEIATSDKVTLTSYSVAPKNWNFGQTFEGKQFEL

>IbEXPA11

MEMAALTLFCIALFCFLTAVRARIPGVYTGGPWQGAHATFYGGADASGTMAGACGYGNLYTQGYGVNNAALSTALFNSGLSCGACFEIKCSNDRSCLAGNPSIVVAATNFCPPNFALPNDNGGWCNPPRLHFDLAMPMFLRIAAYRAGIVPVNFRRVPCKKQGGIRFTINGHQFFNLVIVTNVAGAGDVQNVWIKGTNTQWLAMSRNWGQNWQSSAMLSGQALSFRVRSSDGRKVTSKNISPANWQFGQTYEGKNFRV

>IbEXPA12

MARLSSVVHGSGNGEWSDAHATFYGGADASGTMGGACGYGDLYSEGYGENNAALSSALFKDGLSCGACFEIKCVNDGEECFPGSIIVTATNLCPQNKSLPNNAGGWCNSPLKHFDLSQPIFQHIAQYRAGIVPVKYRRVPCKKRGGIKFSITGHSYYNLVLISNVGGAGDVVSVSIKGSNSSWLPMSHNWGQNWQSKADLNGQALSFKVTTSDARTLVCNNVTPPGWSFGKTYTGGQFSGHESSTSNSSHTTSVNYLYFFVLVLPLLEADPLLQLYTLVKWLRGADGGEWSDAHATFYGGADASGTMGGACGYGDLYGQGYGESNAALSTALFNNGLSCGACFEIKCAGDTEQCFPGSIVVTATNFCPPGGCCDSPFKHFDLSQPIFLQIAQYRAGIVPVQYKRVPCMKIGGIKFTITGHSYYILVLITNVGGAGDVVSVSIKGSNTSWLPMSHNWGQNWQSNAYLNGQALSFVVTTSDGQSVVSNDVTPPSWSFGQTYTGGQFSRGQFPSGQVPAARRSSASKSLSTKHVIFSPFLNVWYPGTAANAHATPQLDELQNVVVSRFGLEMHWFAYFSHRFSFCFLVVTVTIA

>IbEXPA13

METLAIFTLLMLSSMARLRNACGSDGDEWSDAHATFYGGADASGTMGGACGYGDLYGQGYGESNAALSTALFNDGLSCGACFEIKCAGDNEQCFPGSIVVTATNFCPPSTLPSDAGGWCNSPLKHFDLSQPIFQHIAQYRAGIVPVQYRRVPCKKSGGIKFTITGHSFYNLVLITNVGGAGDVVSVSIKGSNTNWLPMSHNWGQNWQSNVYLNGQALSFKLTTSDGLSIVSNNVAPSTWSFGQSYTGGQFSGGQNTSALSPATQPSVGKPSSAHSPVGQPSPSTANSTVGAHFPGDQSPSAQFPGGQPQRHSPSSRSLTTKKLSYLNFLALLLLGIILSII

>IbEXPA14

MGFTRTITLALFLAASTLAAVEARIPGVYQGGAWQTAHATFYGGSDASGTMGGACGYGNLYSAGYGVNTAALSTALFNNGFSCGACFEIKCDDEAGSCHPGRPSILITATNFCPPNYALPNDHGGWCNPPRPHFDLSMPMFLKIAEYQAGIVPVTYRRVPCRKPGGIRFTINGHRFFNLVLVTNVAGAGDVVKVMIKGSRTNWIPLSRNWGQNWQTNAMLVGQPLSFRVKSSDHRTSTSWNIAPSNWQFGQTFVGKNFKI

>IbEXPA15

MEHFRSPFFILVFVLCFHNSCGFFHGGDPSDSGGGWKNGHATFYGGADATGTMGGACGYGNLYSQGYGTNTAALSTALFNTGSSCGACYELKCNEEDPRWCRPGTITVTATNFCPPNYALANDHGGWCNPPLQHFDLAEPAFLQIAQYRAGIVPIAYRRGMRFTINGHSYFNLLLITNVGGPGDVRSVFIKGSKTGWMPMSRNWGQNWETHSYLNGQSLSFRVTASDGRTVAGFDVAPPDWQFGQTFEGGQF

>IbEXPA16

MAISPFFILSLLFFLSSLSFFSHGVFGYGGWQDAHATFYGGGDASGTMGGACGYGNLYSSGYGTNTAALSTALFNNGLKAAVLAMSSGAAPENRVSPGASPSQPPISVRLTLASLITMGVGAILPSNTSISPSLHTCKSLSIVLELCRYLLEEIQTFDLRVPCKRKGGIRFTINGHAFFNLVLVTNVAGAGDVQSVSIKGSGTGWQSMSRNWGQNWQSNSNFNGQTLSFRVTSSDGRTVTSFNAAPANWQFGKTYEGGQF

>IbEXPA17

MGGACGYGNLYSQGYGVNTAALSTALFNSGLSCGACFEIKCDQDRSCYAGRPSIFITATNFCPPNYALPNDNGGWCNPPRPHFDLAMPMFLKIAQYRAGIVPVTYRRVPCRKPGGIRFTINGHRFFNLVLVTNVAGAGDIVKVWIKGSKTNWMPLSRNWGQNWESSAVLVGQALSLRVKASDHRTSTSWNIAPSNWQFGQTFVGKNFRV

>IbEXPA18

MIGPDGAQGFNVPHNRIVLYYYYYISLHSHSYLRLHWFSHDGWSLTSSAAASLQSGGGRRVDLLRACGSRGRGGRGVRVWGFGEERLREGHGGAEHDSVREGQICGACFEVKCVEDLRWCIPGTSIIVTATNFCALTMALSLTAAATVTLPMLISCSPLKPSRKLLSGKLPICPFNIAGSYSVQYTTLLLVGPPDILSLIVFLSYGEGHRHNISVAFMQIKCRKEGGVRFAVSGAGIFISVLIRNVAGTGDIIAVKIKGSRTGWLPMDRVTLTSYNVAPKNWNYGQTFEGKQFEVLAHKQWKSVKAQKHSMFGKEQFHYRFIFTNPKSPLSSSSPARNPLSLSLSVWSLLIVTVD

>IbEXPA19

MREALKNVVCLQCDGPALRRRARPANSQNGECTAQGRESNYLVAGGSAPVDPRNNNNNNPVVVSQPLFTLGGGVQEMEKSAMVDTMAIAMEELIELLQADEPLWIRTPRTDGRNHPRIPPLSPTLLNSSRCVLIQVSGWISSNDVAKARIIEVLDSGMLGGSLQLMFEKMHILSPMVAPRDFIFLRYCRQLDATAWIMVNVSYDCFKENEGFAPSYSWKLPSGCLIQDLHNGKSSQAYGANRWIVTLQRMCERYGFSMGVKGAPTARHELEEVMNEPQGRRSVMQLSQRMVKSFCEILNMGERVDFPQTSEMNNSGVRISLRKAAPETAQSADALIWDVLSNGYPVTEVARIPTGNHPGNCVSIFQPYVSKESSMLVLQESCVNSLEGHVVYAPIDLPVITASINGEDPIKIPMLRRGYKSSNNHHQSVCFSETTPLASASVKSKPVIASRYLASSMPDSTVLLLLYLFAFTLLSTTSLVFSHDGWSLTSSAAASLQSEWRPARATYYAPADPEDAVGGACGYGDLEKRLREGHGGAEHDSVREGSDLRRVLRGQPDGGGHCNPPNAHFVLPIEAFEKIAIWKASNMPIQYRRIKCRKEGGVRFTVSGFKNRMASNGRNWGQNWHINANLRSQPLSLEITSSDRVTLTSYNVAPKNWNYGQTFEGKQFEG

>IbEXPA20

MGIWRRAATGRPRRAEHDSVREGQICGACFEVKCVEDLRWCIPGTSIIVTATNFCAPNYGFEPDGGGHCNPPNAHFVLPIEAFEKIAIWKASNMPIQYRRIKCRKEGGVRFTVSGAGIFISVLIRNVAGTGDIIAVKIKGSRTGWLPMGRNWGQNWHINANLRSQPLSFEITSSDRVTLTSYNVAPKNWNYGQTFEGKQFEG

>IbEXPA21

METLAIFTLLMLSSMARLRNAGGSDGDEWSDAHATFYGGADASGTMGGACGYGDLYGQGYGESNAALSTALFNDGLSCGACFEIKCGGDNEQCFPGSIVVTATNFCPPSTLPSDAGGWCNSPLKHFDLSQPIFQHIAQYRAGIVPVQYRRVPCKKSGGIKFTITGHSFYNLVLITNVGGAGDVVSVSIKGSNTNWLPMSHNWGQNWQISNNVAPSTWSFGQSYTGGQFSGGQNTSALSPATQPSVGKPSSGHTPVGQPSPLHCQFHCQCPLPW

>IbEXPA22

MGVNGLQAVGAVVIMFGVLSLFFVDVKAFTASGWQSAHATFYGGSDASGTMDIINDQGKPSCFSSQKVESPTLKFLNPWSDKVSGKMLVESSISRSLKFRPDRASGRMTAALSTALFNNGASCGQCYKIICDYQADRQWCIKGASITITATNFCPPNYALPSNNGGWVPCKKHGGVRFTINGRDFFELVMINNVGGPGSIQSVQIKGSKTNWIAMSRNWGANWQSSAYLTGQSLSFKVTASDGVTKIFSDVIPAGTAMAELNAVDANSIENSDQGNANQTLRFCSSSALDTAGISRAENNEEEEEELRRLLVPRIEDLPSVPPSAVESNFVTYYAPDFMKPGNDQYVRRHANGLCVIGIASSHVAFKDESGITAVDFNVGKSDRSGIKVTGKRKKVHSCKDENAQHFESNTALCKVCTKDNSYIVRCCVKGSLLEVNERLIERPELLISSAEREGYIAIIMPKPADWLKIKASLLGKEDYCKLRTACIVDSLQ

>IbEXPA23

MPTSRRRLSFTFFMVLVLAAIVDKSVVVGTYSSYRTPVSRPSAWKLAHATFYGDETASATSGGACGYGNLFSNGYGGDTAALSTVLFNNGYACGQCFQIRCTESPYCYKGSPIATVTATNLCPPNWSEDGNNGGWCNPPRTHFDMSKPAFMKIADWKAGIVTVMFRSRYTVDHDSDERPLFRTTAVVLNGWRPIRSCGSFHYKRCRVPCARHGGVKFSIQGNGYWLLVYVMNVAGGGDIAGMWVKGSKTGWVSMSHNWGASYQAFAALGGQTLSFKLTSYTTHETIVAYNVAPSNWNAGMTYQAAVNFH

>IbEXPA24

MLTSGRRLSFTFFMVLVLAAIVDKSMVVGTYSSYRPPVSRPSAWKIAHATFYGDETASATSGGACGYGNLFSNGYGGDTAALSTVLFNNGYACGQCFQIRCAESPYCYKSSPIATVTATNLCPPNWSEDGNNGGWCNPPRTHFDMSKPAFMKIADWKAGIVPVMFRRVPCARHGGVKFSIQGNGYWLLVYVMNVAGGGDIAGMWVKGSRTGWISMSHNWGASYQAFSALGGQTLSFKLTSYTTHETIVAYNVAPSNWNAGMTYQATVNFH

>IbEXPA25

MALIAVSLVVLLAMASNVNGYGTGGWTSAHATFYGGGDASGTMGGACGYGNLYSQGYGTNTAALSTALFNNGLSCGSCYAIRCVGDKSCLPGTIQVTATNFCPPNNGLPNNAGGWCNPPLHHFDLSQPVFQHIAQYRAGIVPVAYKRVPCKRRGGIRFTINGHAFFNLVLVTNVGGAGDVHSVEVKGSRTAWQPMSRNWGQNWQSNANLNGQALSFKVTTSDGRTLVSNNVAPAGWSFGQTYSGAQFR

>IbEXPA26

MGFIIVIFLLAMASAGEGYGGGGWADAHATFYGGGDASGTMGGACGYGNLYSTGYGTNTAALSTALFNNGLSCGSCYEMRCVNDKSCLPGGILVTATNFCPPNSALPNNAGGWCNPPLHHFDLSQPVFQHIAHYSAGIVPVVYRRVACKRSGGIRFTINGHSYFNLVLVTNVAGAGEVHALAIKGSRTGWQSMSRNWGQNWESNLYLNGQALSFKVTTGDGRNIVSYNAAPPNWSFGQTFAGGQFR

>IbEXPA27

MGFLWFFILGLGFLSFSSSAHAKHNDNAGWINAHATFYGGGDASGTMGGACGYGNLYSQGYGINTAAVSTALFGNGLRCGACFEVKCVNDPKACLPASVVVTATNFCPPNSALPSNQGGWCNPPLHHFDLSQPVFQQIARYKSGIVPVSYRRVACQKKGGIRFTINGHSYFILVLITNVGGSGDVHAVSIKGTNTNWMPMSRNWGQNWESNSKLDGQALSFKVTTGDGRTVVCNNVVPFEWSFAQTYIGAQFT

>IbEXPA28

MGFLWFFILGLGFLSFSSSAHAKHNDNAGWINAHATFYGGGDASGTMGGACGYGNLYSQGYGINTAAVSTALFGNGLRCGACFEVKCVNDPKACLPASVVVTATNFCPPNSALPSNQGGWCNPPLHHFDLSQPVFQQIARYKSGIVPVSYRRVACQKKGGIRFTINGHSYFILVLITNVGGSGDVHAVSIKGTNTNWMPMSRNWGQNWESNSKLDGQALSFKVTTGDGRTVVCNNVVPSECYHHHHEHTLASTTTITLPSLSLASSPPLPPPPPRHIHRHYHHHDASITITTTTTTNTNTNTNTHYTTSTIIITTSHIHTTTTTIITRLPPPPPLSLSSHHHHTTTIHIIRDPTTMRCIFVKNVTGDTATGTEKAGGSHSPLGEGDLDLGCLRLRWRRVEEQGARYGPRTDLWERALRSKVPVMVLEPILGFSAHCGSRVPVMVLVSIRGSKVPVEVLEPICRSKVPTTGSRCPLWSSDQSFWVYSAHYGSKVPVTVLEPSRCHGPQTHLFGLVPITGARCPFICPVIGRWEQSAHSRSLRYANSIKCSSPPVRSCDRAKLKF

>IbEXPA29

MAAEMRLWLLIVLVVLPCAVFAHSWNEAHATYYGAPEGTIGGACGFEEYKQIYSPYTAALSPTLYNEAESCGACFEIVCVNTTGFCKKDGEKSVVVTATDLCPASDARCSPPHVHFDLSQPAFHLIAEHLGGDVPVKYRKVSCKRKGGAKFTITGNPNFNLVTVTNVAGGGDVEKVEVKAEGDKEWKNMKRNWGEKWETSEVLSGKSLNFRVTTTEGEVVTSKHVAPESWQFGQTFEGKNV

>IbEXPA30

MELRAPLCSPLLIVLVVLPCAFAHTSKWKEAHATYYGSPDGTIGGACGFEEYKKTYGSYTAAVSKPLFDNAAGCGACYEVRCVKNKMYCKKGHKSVVVTVTDLCPPGGWCSHTHFDLSQPAFLKIADQVAGHVPVMYRSVPCKRKGGEKFKISGNPFFNLVTVTNVGGEGDVKKLEVKPEGSKKWKSLKRNWGENWECDEKLTGKALTFKVTTVDGKESVSRNVAPKSWKFGQTFEGKNF

>IbEXPA31

MAPELQASLLIVLCALPCVFGGDDGWRDAHATFYGPPEGTIALSAALFKDAASCGACYELKCENSTGHCKTTEKAIIVTATDLCPTGDGKWCGPPLEHFDLSMPAYLQIGIYEAGFIPVRYRREEKWTQLKRNWGEKWETNEALSGKSLNFRVTTNDGKTVTSRKVAPKGWQYGQTFEGKNSR

>IbEXPA32

MAEPEGRRLVIVSRLLELIGDGDGRHFSGRRPFWSHLVRLRDIVAFSIPRWRRLAISGEDGDKMGRRRRPLPIAFFALRRWRLVAISNRLEKETRQSPSPSTEMATSSRHFKSPEPSPADFVLRGRFLLLLTQIFEWAELQASLLLIVLCALPCVFGGDPQGGWLEAHATFYGHPAGTIGVEKLLLRGACGLEEFKQTYSPYTTALSPALFNNAASCGACYEIKCVNTTGQCKAPQQSITVTATDLCPTGDGKWCAPPLAHFDLSMPAYLRIGEYKAGVIPVYYRRVPCVRKGGERFTISGHQYFNLVTVSNVGGSGDVKMVEVNAEGDEKWTQLKRNWGEKWETNEKLSGKSLTFRVTTNDGKTVTSQNVAPKSWQYGQTFEGQTDN

>IbEXPA33

MGLVEISNAESPPKEVHSNACDHRTRAGDGRDDLLRVILEQVFREFNRWKNEQNPSSSEVIPPLLATHFTDEIMNHHYPIDLRVPGNKGYNGKTNPEEHVNSYYVFLSLREGLFGGRLAERDFTYLEVAQQKERETLTDFLAKWKNNAVGEIKPIDDHTAINVLHSSLRVGALYQDFILKPPLTYEEAFRRVADHANAGQVRQMWQRCDKPPTFTPRTRPVVEVLDYTQSCNLFRLPEPTRDGEDKSKYCAYHRNRGHDTEDCNILRRVIDRSIIVWFGQLYCEHFLLIVLCALPCVFGGDPQGGWLEAHATFYGHPAGTIGGACGLEEFKQTYSPYTTALSPALFNNAASCGACYEIKCVNTTGQCKAPQQSITVTATDLCPTGDGKWCAPPLAHFDLSMPAYLRIGEYKAGVIPVYYRRVPCVRKGGERFTISGHQYFNLVTVSNVGGSGDVKMVEVNAEGDEKWTQLKRNWGEKWETNEKLSRKSLTFRVTTNDGKTVTSQNVAPKSWQYGQTFEGQN

>IbEXPA34

MEALRLLCIALLLCSLTAVHARIPGVYSGGAWQSAHATFYGGADASGTMGGACGYGNLYSQGYGVNNAALSTALFNNGLSCGACFEIKCTNDRSCSPGNPSIFITATNFCPPNFALPNDNGGWCNPPRPHFDLAMPMFLKIAAYRAGIVPVNFRRIPCRKQGGIRFTVNGFRYFNLVLVTNVAGAGDIQKVLVKGSRTPWITLSRNWGQNWQTSSFLVGQSLSFRVRASDRRQVTSWNVAPANWQFGQTFQGKNFRA

>IbEXPA35

MPMLSQLLGGKGLMQPFYGGSDASGTMGGACGYGNLYTTRYGTRTAALSTALFNDGASCGQCYKIICDFKAEPRWCRKGVSVTITATNFCPPNYALPSDNGGWCNPPGPHFDMAQPAWEKIGIYSGGIVPVIYQRVPCKKRGGVRFTINGHDYFELVLVTNVGGAGSIRSVQIKGSKTNWMTMSRNWGVNWQSNAYLTGQSISFRVTSSDGVTMTFLNVAPSNWRFGQAFASPLQFS

>IbEXPA36

MASFLRRLSFAFFCLLAVIGKATAAGYYSHPVFHTGAWKLAHATFYGDESASETMGGACGYGNLFNNGYGTATAALSTVLFNNGFACGQCFQIRCAQSPYCYKGFPTTTVTATNLCPPNWAEDSNNGGWCNPPRTHFDMSKPAFMKIAQWKAGIVPVMYRRVPCIRSGGVRFNLQGNGYWLLVYVMNVGRGGDIAAVWVKGSRTGWIRMSHNWGASYQAFGTLGGQSLSFKLTSYSSHETIVAYNVVPSNWNVGLTYQAKVNFH

>IbEXPB1

MPHRGACGYGSTVEQPPFSSFVSAGGPSLFKSGKGCGACYEVKCTANKACSGKPIRVVITDECPGCVKESTHFDLSGTAFGAMAVSGKSNQLRAAGEIKIQYKRVECNHRGKTLTFRVDPGSNPFYFAAVIQYAQGDGNIVAVQLKQGNSGVWTDMKQSWGAMWKLNSGSRLNAPFSLKVTGDSGKSVVAGNVIPAGWRPGGTYRSG

>IbEXPB2

MPHFITYFLLFSTLAISCSCFNTKNLNISKSKAISDWSPAGATWYGNPNGAGSDVSVQHNCSISYDHGSQCIVDHGGACGYRNSVEQPPFSSLVTAGGPSLFKSGKGCGACYEVKCTENAACSGKPVTVVITDECPGCVSESTHFDMSGTAFGAMASSGKADQLRNAGLLNVHYRRVECKYPGVPLSFHLDSGSNPYYFALVIEYVGGEGVLSAVELKQAFTDSWLPMQRSWGAVWMLQDPSD

>IbEXPB3

MNSLLPSNTVAQSLPLPSFFFVDPHRRTPSQRSSSLTRSASPPAPKFFHNGLMIELQHEFMHIGFISMPVCYHKEVFDTIDISFNKTVKEFRKIVNLNISQLDPDWSPAGATHYGDRNGAGSTGGACGYTNSVAQPPFSSFVSAGGSSLFKSGKGCGACYEVKCTENAACSGNPVTVPWQFQAMPISSEPPEYSTSYIEVSFGVLSGSSPYYFETLIEYVSGSGALSAVELKQASASPDSWLSMQQSWGAVWKLENHSGLTAPPFSLKLIDESGNTRRRQRTPGECFVWLEYMLFRILNGNNCVARCLVNSCTGSNMVFWKKEGLPRSLGRILTLEGIPHFVFAPGVDTYNMRLILSDWGGFQLQELGFNAEGSHNEGWQLNGSIYWMFKVVRERGRLIA

>IbEXPB4

METPMVPEVMVRGACGYTTSVEQPPFSSLVTAGGPSLFKSGKGCGACYEVKCTENAACSGNPVTVVITDECPGCVSESTHFDMSGTSFGAMAVSGKADQLRNAGVLNIQYRRVQCKYPGISLTFHVDSGSNPNYFATLIEYEGGEGVVSSVELKQALDSDSWLPMQESWGAVWKLDASGLKAPFSLKLTDDSGKTLVANNVIPAGWQPGNSYRSPRWCNHPSPLFSFTPRREFHYFVHPDTNSKTPVLATQALPECLAQSYTQPRARDKAKLPACTASAPASHYARPSVSHKATLSSRVSATYTPFYT

>IbEXPB5

MPHFITYFLLFSTLAISCSCFKHKHLKPKNFKVSKSQSDSGWSPAGATWYGNPNGAGSDGGACGYTNSVDQPPFSSLVSAGGPSLFNSGKGCGACYEVKCTENAACSGNPVTVVITDECPGCVSESTHFDMSGTAFGAMAVSGKADQLRDAGVLNIQYRRVKCKYPGVSLTFHIDSGSNPNYFATLIEYEGGEGVVSSVELKQALDSDSWIPMQESWGAVWKLDDGSALKPPFSLKLTDDSGKTLVANNVIPAGWQPGKNLSICR

>IbEXPB6

MPHFITYFLLFSTLAISCSCINQNNFSQSDPDWSPAVATNYNGPNGGGSTDGACGYKNSVAQPPFSSFVSAGGSSLFKSGKGCGACYEVKCTENAACSGNPVTVVITDSCLDSNSQSQPVHFDMSDTAFVAMAIPGNADPLRTAGVLNVQYRSCVERDLQFRGTNAVSSIRNCSYVEWILQLCLKDTAVMLNGHCSYVERMNDHCSVQLQFPFNITAISFAHNYSICSTQLQVQCKYPGVPLSFGVRSGSSPYYFETLIEYVSGSGALSAVELKQASASPDSWLSMQQSWGAVWKLNDTSGLTAPFSLKLIEDSGKTLVADNVIPADWQPGKTYQSAVTSFV

>IbEXPB7

MPHFITYFLLFSTLAISCSCFNTKNLNISKSKAISDWSPAGATWYGNPNGAGSDGGACGYRNSVKQPPFSSFVTAGGPSLFKSGKGCGACYEHVRGKPVTVVITDECPGCVSESTHFDMSGTAFGAMAASGKADQLRNAGLLNVHYRRVECKYPGVPLSFHLDSGSNPYYFALVIEYVGGEGVLSAVELKQAFTDSWLPMQRSWGAVWMLQAASGLKPPFSLKLTDDSGKTLVAHNVIPAGWQPGNTYQSVVTSFV

>IbEXPB8

MPHFITYFLLFSTLAISCSCFNTKNLNISQLDPDWSPAGATHYGDRNGAGSTGGACGYTNSVAQPPFSSFVSAGGSSLFKSGKGCGACYEVKCTENAACSGNPVTVVITDSCPGSDCGSQPVHFDMSDTAFVALAIPGNADQLRTAGVLNVLYRRVQCNYPGVPVSFGVLSGSSPYYFETLIEYVSGSGALSAVELKQASASPDSWLSMQQSWGAVWKLENHSGLTAPPFSLKLIDESGNTLVADNVIPANWQPGTTYQSAVTPFA

>IbEXPB9

MPHFITYFLLFSTLAISCSCFNTKNLNISKSKAISDWSPAGATWYGNPNGAGSDGTRYTVDHGPQCIVDHGSAVVLNDRNRVEPSAVVLKGNYSCAEQRPCVERMNGLAAQLQFPFNTTAVSFQHNCSGACGYRNSVEQPPFSSLVTAGGPSLFKSGKGCGACYEVKCTENAACSGKAVTVVITDECPGCVSESTHFDMSGTAFGAMAASGKADQLRNAGLLNVHYRRVECKYPGVPLSFHLDSGSNPYYFALVIEYVGGEGVLSAVELKQAFTDSWLPMQRSWGAVWMLQDPFGLKPPFSLKLTDDSGKTLVAHNVIPAGWQPGNTYQSVVTSFV

>IbEXPB10

MGGRHFRFAATCFFAAVLVAAAGPLRRVEDPHWYPAAATWYGSPEGDGSDGGACGYGTMVDVKPFRARVGAVSPVLFKGGEGCGACYKVKCLDRSICSRRAVTVIITDECPGCANDRVHFDLSGAAFGRMAVTGYGGSLRNRGELPVIYRRTPCKYPGKDIAFRVNEGSTDFWLSLLVEFEDGDGDVGSMHIRQAMSNEWLEMNHIWGANWVINGQGGGALQGPFSVKLTTLSTGRLLSARDVIPRNWSPKATYTSRLNFHI

>IbEXLA1

MAPIIIIISICLFLLLTFSSTTACDRCVHQSKLSSVLQSAGACGYGSMAVSFNGGHIAAAGVKIYKDGARCGACYQIRCKNPNFCTNHGTIVMVTDLVTSTNETDFVVSTGTLRAMANMPNDQTILQLVNSNLDVEYKRVACEHGTNNLAIRVEESSQRPSHLAVTFLNQGGQTEIIAVDVAPVGFPNWSFMSRSTRGGAIWETSAAPSGPLQFRLVVTAGFDGKWYWASNALPADWETGMIYDSGIQITDIAQETNCSPCDDGTW

>IbEXLA2

MAPPDHHLWRLCFLVSSILISSASACDRCVHQTKVSFFSKASVLQSGACGYGSMAIAFNGGHLAAAVPTLYKDGDGCGACFQMRCKDPNLCTKQGTTVIVTDLNTNNQTDFVISSRAFRAMATQGKDQDLLKLGIVDVDYKRVPCNYKTKNLAVFVEETSQKPNYLAIKFLYQGGQTEIVAVDVAQVGSSNWNFMSRNHGAIWDTSRVPSGALQFRFVVTAGYDGKWYWAKNVMPADWKNGVIYDAALQITDIAQEGCSPCDHDDWTS

>IbEXLB1

MGLGIMNMCIFLFVTVIFPVMCYSEQIENSLYSRATYYGSPDCYGTPTGACGFGDYGRNVNGGKVSGVSKLYKNGTGCGACYQVRCKIPTHCNEEGTRIVVTDYGEGDRTDFILSTRAYSEMANTGLANELIALGVVDVEYRRIPCRYNGYNLMIQVHDKSDFPNYIAIMPLYQSGLSDITAAQIWQADCKEWRDMRRVFGGVYDYQNPPKGSISFRLQTNLNGNTKWVEMLDILPQQWKVGVAYDTNIQLD

>IbEXLB2

MGLGIRNMCIFLFVTVILPVMCYSEQIENSVYTRATYYGSPDCYGTPTGACGFGDYGRNVNSGKVSGVSKLYKNGTGCGACYQVRCKIPTHCNEEGTRIVVTDYGEGDRTDFILSTRAYSEMANTGLANELIALGVVDVEYRRIPCRYNGYNLMIQVHDKSDFPNYIAIMPLYQSGLSDITAAQIWQADCKEWRDMRRVFGRVYDYQNPPRGSLSFRLQTNLNGNTKWVEMLDILPQQWKLSRQDILEVIRKYRDSHAKPKRVERLAPQYHIELNPCACELMYLRGLPCKTISGLQIVQGSLYFDPIDRAVETGRNSSSLMCRVVVKAAVLLLHAPLYLLVVRVGCCMPNYPVTSPPTPWQNSAVEVGPQPNVVEAKPRPNVAKATPWPNAADASSEALPIVGLIPIFFFGALCASLLRLAVGSSKGQLFRRVVSAVKPRTTP

>IbEXLB3

MVFTLKISCFTLLCIFLIYPPALCHSKLVGSKATFYKQPDGMGTPDGACGYKDYGRTVNDGAVCTVSNKLFNNGAGCGSCYNNSGFYHVNGAFGSHMRIGMGIKYLVICTNKALCSSAGTKVVATDNGGGPAGSDFICSYLAFTKLAKPGKESQLVKKGVIDVVYEKVACNYPKNLIIKITDQSSNPGYLSFALLNQGDILSAEVYDMGSQGWTSMRRVYGAVFDLANPPEGALKVRFTIARLFELELDLELEFELEFELEFMLELKFEEPEVPPSRRRRGTSGSAGRSANQESPGEGDNCRLRSAPTVRLGRRHLPANRWFPFAEGDELAFLGSPSPSDGSPSPAPVIVR

>IbEXLB4

MAFPLKQRCTLFCMILLLSAANYCYSQTYLSKATYFTTDDGMGNPTGACGYGQYGKTANGGQVFAASGRLYQNGAGCGACYLVKCKEEGLCRDGGVKVMATDSGEGPGTDFILSYRAYGKMAVPRMASLLYSLGVVDVEYRRISCHEAANLVIKIVEHSNYPWYLAIVPLKQGGANDILSIEKELRAKDLVVQSTVASNPSLYTGGGGFEPQWSQYCIKASDTWTAMRRVYGAVFDFQNPPLGELKIRLSVNGSDGQKWVESDKAAIPKYWKAGITIKTDIQLT

>IbEXLB5

MSWKGLNLCAPALAGTNVVRKIGMPRSSLGAASRLQCLGGDGACGYGEYGRTTNDGQVCAVSTRLFKNGAGCGACYQVRCKSSGLCSEVGEKVMATDYVEGQETDFILSYRAYTGLAKQPRMAEFLREKGAVDVEFRRVSCNSAAKLRVKIHDNSQYPHYLSILLTNQGGATDIFAVEIYEEETYEWISMRRAFGAVWDLANPPSGELKARFLVSSSAAAKWVDSEKAIIPAEWKAGLTIETNIKLN

>IbEXLB6

MGLGIKNLCLFLFVTVLILPVLSYGEQLENSLYTRATYYGSPDCYGTQSGACGFGDYGHNGGEWCGVSRLYRNGTGCGACYQVRCKIPTHCNEEGTRIVVTDYGEGDRTDFILSARAYSEMATPGLANQLFAFGVVDVEYRRIPCRYNGYNLMIQVHDKSNFPNYIAILPIYQSGLSDITAAQIWQADCKEWRDMRRVFGGVFDYQNPPKGSLSFRLQTSLNGNTKWLEVSNVLPDEWKAGVAYDTYIQLD

>IbEXLB7

MGLGIKNLCMFLFVTVLLLPVLSYGEQLENSLYTRATFMEAQIAMELKGDPSLNGFESNTNYGLDSLVWTITKGGACGFGEYGRNVNGGKVSGVSRLYRNGTGCGACYQVRCKIPTHCNEEGTRIVVTDYGEGDRTDFILSARAYSEMATPGLANQLFAFGVVDVEYRRIPCRYNGYNLMIQVHDKSNFPNYIAILPIYQSGLSDITAAQIWQADCKEWRDMRRVFGGVFDYQNPPKGSLSFRLQTSLNGNTKWLEVSNVLPDEWKAGVAYDTYIQLD

>IbEXLB8

MAIFFKYFCTLLSITLVIPALANPQGGGGGGGSTQPGGDGGSASSQPAGPKATYYTTSDGLGTPSGSCGYGELGRTENNGEVCTATSRLYNGGAGCGACYQVRCKNKDLCSEEGTKVVVTNSGEGPATDFILSYTAYAKLAKYPAVAAQLFAQGVVDIEYRRVSCKFGANLMIRIQEHSKFPSFLSIVVMNQGGATDILAVEIYEESSQKWIAMRRAYGAVWDLSSPPCGRLKVRFLVSSSDGTTAWVQSDKAAIPADWKAGVTVETDITLS

>IbEXLB9

MGFSLKQGCTLLCILMVLPAAFCYTQTYPSKATYYTTPDGKGTPTGACGYGQYGRSINDGLVSAASSRLYRYGAGCGACYLVRCKDEALCNKEGVKVVITDNGEGPATDFILSHNAYAGLAKPYAAKDLFARASSTSTTKEFLVGTASLRSKLLSRATIMDILLFSPSITVVPMIFSPLRSMRFKASYKWIPMRRSYGTVFDLQNPPKGELKLRFLVSGQKWIESEKAIIPDYWKAGTTIDTHIQLP

>IbEXLB10

MGFSLKQGCTLLCILMVLPAAFCYTQTYPSKATYYTTPDGKGTPTGACGYGQYGRSINDGLVSAASSRLYRYGAGCGACYLVRCKDEALCNKEGVKVVITDNGEGPATDFILSHNAYAGLAKPYAAKDLFARGVIDVDYERVSCGHGKLKIKIVEQSNYHGYLAILPFNQGGANDILSIEVYEKASYKWIPMRRSYGTVFDLQNPPKGELKLRFLVSGQKWMESEKAIIPDYWKAGTTIDTDIQLP

>IbEXLB11

MAVLQCLAVLIGTFLVIQSPGDAATCSDCFIQSQATYYPNSDDNGTDSRVLDLTLQITGQQFLSHLPGGAGLALNQPPPTIFNNMLMTDFMLNAIYAGGHCGFGTFGATMNGGDVSAASNLYRDGLGCGACYQVRCTNSLYCSDKGVTAVITDSGASGGSDFILSSGAFGRMAQTKDAARSLLSQGVVDIEYRRVPCSYPGKNITFRIHDKSGNPHYLAFVIMFEQGQKDITAVLLCETQNLQCKLMDRTYGSVWTSNSPPSGALEVRMLFSGEEDGDETWVVSPNNIPGTWKPGDTYDSGVQVNAAA

>AtEXPA1

MALVTFLFIATLGAMTSHVNGYAGGGWVNAHATFYGGGDASGTMGGACGYGNLYSQGYGTNTAALSTALFNNGLSCGACFEIRCQNDGKWCLPGSIVVTATNFCPPNNALPNNAGGWCNPPQQHFDLSQPVFQRIAQYRAGIVPVAYRRVPCVRRGGIRFTINGHSYFNLVLITNVGGAGDVHSAMVKGSRTGWQAMSRNWGQNWQSNSYLNGQSLSFKVTTSDGQTIVSNNVANAGWSFGQTFTVEAVRERGMIVIWSFLSIEVNLKRSGASSA

>AtEXPA2

MNLTEYSHILFLSLCTLNFCLYSINSDDNGGWERGHATFYGGADASGTMGGACGYGNLHSQGYGLQTAALSTALFNSGQKCGACFELQCEDDPEWCIPGSIIVSATNFCPPNFALANDNGGWCNPPLKHFDLAEPAFLQIAQYRAGIVPVAFRRVPCEKGGGIRFTINGNPYFDLVLITNVGGAGDIRAVSLKGSKTDQWQSMSRNWGQNWQSNTYLRGQSLSFQVTDSDGRTVVSYDVVPHDWQFGQTFEGGQF

>AtEXPA3

MTATAFRVGLWLAVTASFLLTATNAKIPGVYSGGPWQNAHATFYGGSDASGTMGGACGYGNLYSQGYGVNTAALSTALFNNGFSCGACFEIKCTDDPRWCVPGNPSILVTATNFCPPNFAQPSDDGGWCNPPREHFDLAMPMFLKIGLYRAGIVPVSYRRVPCRKIGGIRFTVNGFRYFNLVLVTNVAGAGDINGVSVKGSKTDWVRMSRNWGQNWQSNAVLIGQSLSFRVTASDRRSSTSWNVAPATWQFGQTFSGKNFRV

>AtEXPA4

MAIKLAILFTTFVLFSLADARIPGIYSGGAWQNAHATFYGGSDASGTMGGACGYGNLYSQGYGTNTAALSTALFNNGMSCGACFELKCANDPQWCHSGSPSILITATNFCPPNLAQPSDNGGWCNPPREHFDLAMPVFLKIAQYRAGIVPVSYRRVPCRKRGGIRFTINGHRYFNLVLITNVAGAGDIVRASVKGSRTGWMSLSRNWGQNWQSNAVLVGQALSFRVTGSDRRTSTSWNMVPSNWQFGQTFVGKNFRV

>AtEXPA5

MGVLVISLLVVHLLAFSVCVQGGYRRGGHHPGGHMGPWINAHATFYGGGDASGTMGGACGYGNLYSQGYGLETAALSTALFDQGLSCGACFELMCVNDPQWCIKGRSIVVTATNFCPPGGACDPPNHHFDLSQPIYEKIALYKSGIIPVMYRRVRCKRSGGIRFTINGHSYFNLVLVTNVGGAGDVHSVSMKGSRTKWQLMSRNWGQNWQSNSYLNGQSLSFVVTTSDRRSVVSFNVAPPTWSFGQTYTGGQFRY

>AtEXPA6

MAMLGLVLSVLTTILALSEARIPGVYNGGGWETAHATFYGGSDASGTMGGACGYGNLYSQGYGVNTAALSTALFNNGFSCGACFELKCASDPKWCHSGSPSIFITATNFCPPNFAQPSDNGGWCNPPRPHFDLAMPMFLKIAEYRAGIVPVSFRRVPCRKRGGIRFTINGFRYFNLVLVTNVAGAGNIVRLGVKGTHTSWMTMSRNWGQNWQSNSVLVGQSLSFRVTSSDRRSSTSWNIAPANWKFGQTFMGKNFRV

>AtEXPA7

MGPISSSWSFNKFFSIVFVVFAISGEFVAGYYRPGPWRYAHATFYGDETGGETMGGACGYGNLFNSGYGLSTAALSTTLFNDGYGCGQCFQITCSKSPHCYSGKSTVVTATNLCPPNWYQDSNAGGWCNPPRTHFDMAKPAFMKLAYWRAGIIPVAYRRVPCQRSGGMRFQFQGNSYWLLIFVMNVGGAGDIKSMAVKGSRTNWISMSHNWGASYQAFSSLYGQSLSFRVTSYTTGETIYAWNVAPANWSGGKTYKSTANFR

>AtEXPA8

MYTPSYLKYSIISIISVLFLQGTHGDDGGWQGGHATFYGGEDASGTMGGACGYGNLYGQGYGTNTAALSTALFNNGLTCGACYEMKCNDDPRWCLGSTITVTATNFCPPNPGLSNDNGGWCNPPLQHFDLAEPAFLQIAQYRAGIVPVSFRRVPCMKKGGIRFTINGHSYFNLVLISNVGGAGDVHAVSIKGSKTQSWQAMSRNWGQNWQSNSYMNDQSLSFQVTTSDGRTLVSNDVAPSNWQFGQTYQGGQF

>AtEXPA9

MAAKVITFMAVMVVTAFTANAKIPGVYTGGPWINAHATFYGEADASGTMGGACGYGNLYSQGYGVNTAALSTALFNNGLSCGSCFELKCINDPGWCLPGNPSILITATNFCPPNFNQASDNGGWCNPPREHFDLAMPMFLSIAKYKAGIVPVSYRRIPCRKKGGIRFTINGFKYFNLVLVTNVAGAGDVIKVSVKGSNTQWLDLSRNWGQNWQSNALLVGQSLSFRVKTSDGRSSTSNNIAPSNWQFGQTYSGKNFRV

>AtEXPA10

MCRLLTQDVNMGHLGFLVMIMVGVMASSVSGYGGGWINAHATFYGGGDASGTMGGACGYGNLYSQGYGTSTAALSTALFNNGLSCGSCFEIRCENDGKWCLPGSIVVTATNFCPPNNALANNNGGWCNPPLEHFDLAQPVFQRIAQYRAGIVPVSYRRVPCRRRGGIRFTINGHSYFNLVLITNVGGAGDVHSAAIKGSRTVWQAMSRNWGQNWQSNSYLNGQALSFKVTTSDGRTVVSFNAAPAGWSYGQTFAGGQFR

>AtEXPA11

MSKSLAGLAVLAALFIAVDAFRPSGLTNGHATFYGGSDASGTMGGACGYGDLYSAGYGTMTAALSTALFNDGASCGECYRITCDHAADSRWCLKGASVVITATNFCPPNFALPNNNGGWCNPPLKHFDMAQPAWEKIGIYRGGIVPVVFQRVSCYKKGGVRFRINGRDYFELVNIQNVGGAGSIKSVSIKGSKTGWLAMSRNWGANWQSNAYLDGQALSFSITTTDGATRVFLNVVPSSWSFGQIYSSNVQF

>AtEXPA12

MDMKGTYLVTVILLVSTLSVGMCSNGWIRAHATYYGVNDSPASLGKKAIKLLFNIRFCYFKVCMVNLFFRYNFIGGACGYDNPYHAGFGAHTAALSGELFRSGESCGGCYQVRCDFPADPKWCLRGAAVTVTATNFCPTNNNNGWCNLPRHHFDMSSPAFFRIARRGNEGIVPVFYRRVGCKRRGGVRFTMRGQGNFNMVMISNVGGGGSVRSVAVRGSKGKTWLQMTRNWGANWQSSGDLRGQRLSFKVTLTDSKTQTFLNVVPSSWWFGQTFSSRGRQFV

>AtEXPA13

MQRFLLPLLFLALSPPAICHYSSSTSSPSSSSVSSDASEWRPARATYYAATNPRDAVGGACGYGDLVKSGYGMATVGLSETLFERGQICGACFELRCVDDLRWCIPGTSIILTATNFCAPNYGFDPDGGGHCNPPNKHFVLPIEAFEKIAIWKAGNMPVQYRRINCRKEGSMRFTVDGGGIFISVLITNVAGSGDIAAVKIKGSRTGWLPMGRNWGQNWHINADLRNQALSFEVTSSDRSTVTSYNVSPKNWNYGQTFEGKQFETP

>AtEXPA14

MEFFGKMIISLSLMMMIMWKSVDGYSSGWVNARATFYGGADASGTMGGACGYGNLYSQGYGTNTAALSTALFNGGQSCGACFQIKCVDDPKWCIGGTITVTGTNFCPPNFAQANNAGGWCNPPQHHFDLAQPIFLRIAQYKAGVVPVQYRRVACRRKGGIRFTINGHSYFNLVLITNVAGAGDVISVSIKGTNTRWQSMSRNWGQNWQSNAKLDGQALSFKVTTSDGRTVISNNATPRNWSFGQTYTGKQFRAQSYDPLENSSGAPSPISSNFIFSSSFAIKLLASTFPSLLIMCFNQAFLC

>AtEXPA15

MFMGKMGLLGIALFCFAAMVCSVHGYDAGWVNAHATFYGGSDASGTMGGACGYGNLYSQGYGTNTAALSTALFNNGLSCGACFEIKCQSDGAWCLPGAIIVTATNFCPPNNALPNNAGGWCNPPLHHFDLSQPVFQRIAQYKAGVVPVSYRRVPCMRRGGIRFTINGHSYFNLVLVTNVGGAGDVHSVAVKGSRTRWQQMSRNWGQNWQSNNLLNGQALSFKVTASDGRTVVSNNIAPASWSFGQTFTGRQFR

>AtEXPA16

MAINPLILLTIFPLFLLLSFTDAGIPRVFSGGSWQTAHATFYGGNDASGTMGGACGYGNLYSQGYGTNTAALSTSLFNSGQSCGACFEIKCVNDPKWCHPGNPSVFVTATNFCPPNLAQPSDNGGWCNPPRSHFDLAMPVFLKIAEYRAGIVPISYRRVACRKSGGIRFTINGHRYFNLVLITNVAGAGDIARTSVKGSKTGWMSLTRNWGQNWQSNAVLVGQSLSFRVTSSDRRTSTSWNIAPSNWQFGQTFVGKNFRV

>AtEXPA17

MTKIFSLLVAMIFSTMFFMKISSVSAGWLQAHATFYGGSDASGTMGGACGYGNLYTDGYKTNTAALSTALFNDGKSCGGCYQILCDATKVPQWCLKGKSITITATNFCPPNFAQASDNGGWCNPPRPHFDMAQPAFLTIAKYKAGIVPILYKKVGCRRSGGMRFTINGRNYFELVLISNVAGGGEISKVWIKGSKSNKWE

TMSRNWGANYQSNTYLNGQSLSFKVQLSDGSIKAALNVVPSNWRFGQSFKSNVNF

>AtEXPA18

MDQNLYRKCLVILSMMAMIGTSMATYAGTPWRTASATFYGDDTGSATMGGACGYGNMYDSGYGVATTALSTALFNEGYACGQCFQLKCVSSPNCYYGSPATVVTATNICPPNYGQASNNGGWCNPPRVHFDLTKPAFMKIANWKAGIIPVSYRRVACKKIGGIRFKFEGNGYWLLVYVMNVGGPGDIKTMAVKGSRTGWINMSHNWGASYQAFSSLYGQSLSFRLTSYTTRQTIYAYNAAPASWSAGKTYQSKANFN

>AtEXPA19

MNRSITFVVLLAVLVSVANLGLAHVGLTNIDPSWYDAHATFYGDMSGGETMQGACGYGDLFKQGYGLETAALSTALFNNGQTCGACFELMCVSSKWCKPNAGSIKITATNFCPPNYQEPVQYHWCNPPNKHFDLSMKMFTTVAEYRAGIVPVKFRRVACHKRGGVRFEIKGNPYYIMVLVYNVGGAGDVSNVEIRGQKSNWIVMKRNWGQIWDTGLDLVGQSLSFIVRTSDGRSMTFFNVAPPNWGFGQTYEAKSNF

>AtEXPA20

MDSGLQQLALCLFFILCRLFQATAEDDWKIATATLSRDRDGSSSVATGGACGYGDLRQSSFAGYSAGLSGKLFNRGSSCGACLEVRCVNHIRWCLQGSPSVVVTATDFCPPNSGLSSDYGGWCNFPKEHLELSHAAFTGIAETRAEMIPIQYRRVKCGRRGGLRFSLSGSSHFFQVLISNVGLDGEVVGVKVKGHTTAWIPMARNWGQNWHSSLDLIGQSLSFEVTLKGGKTIASYDVAPPYWRFGMTYQGKQFHS

>AtEXPA21

MKLLEKMTYVECFMIIMATWFMFISYSHGANVAAAPGTNGLDTAWYDARAAYYGDIHGGGTELEGACGYGDLNKHGYGLATAALSTALFNSGASCGACYEIMCSPNPQGCLSGSIKITATDLCPPGSAWCYLPNKHFDLSLPMFIKIAQVKAKMVPVRYRRVPCAKTGGVKFEVKGNPNILTILPYNVGGAGDIIAVSAKGSKTAWVVMSRYWGQNWTTNVNLTGQSVSLRVTTSDGITKDFTDVMPASWGFGQTFDGKTNF

>AtEXPA22

MKLLEKMIYVEFLMIIMVIWVVPMSYGHGAMIGNAVEAPDVAEAPGINDPSKALDTNWYDARATFYGDIHGGDTQQGACGYGNLFRQGYGLATAALSTALFNDGYTCGACYEIMCTRDPQWCLPGSVKITATNFCPANYSKTTDLWCNPPQKHFDLSLAMFLKIAKYKAGVVPVRYRRIPCSKTGGVKFETKGNPYFLMVLIYNVGGAGDIKYVQVKGNKTGWITMKKNWGQNWTTITVLTGQGLSFRVTTSDGITKDFWNVMPKNWGFGQTFDGRINF

>AtEXPA23

MNLLGKMIYVEGFMMIMATLLVSMSYGHRAMINDVAEAPVFDDVVSPNGLDSSWYDARATFYGDIHGGETQQGACGYGDLFKQGYGLETAALSTALFNEGYTCGACYQIMCVNDPQWCLPGSVKITATNFCPPDYSKTEGVWCNPPQKHFDLSLPMFLKIAQYKAGVVPVKYRRISCARTGGVKFETKGNPYFLMILPYNVGGAGDIKLMQVKGDKTGWITMQKNWGQNWTTGVNLTGQGISFRVTTSDGVTKDFNNVMPNNWGFGQTFDGKINF

>AtEXPA24

MELLKRKLYAKILMMVMVIWIAPMTNGHDHASHVPGGRPGAHPSHGAHPAHGAHPSHGAHPSHGAHPSHGAHPSHGALPSHGGQVPHSGWGHGRATFYGDINGGETQQGACGYGDLHKQGYGLETAALSTALFNNGSRCGACYEIMCEHAPQWCLPGSIKITATNFCPPDFTKPNDNWCNPPQKHFDLSQPMFLKIAKYKAGVVPVKFRRVPCAKIGGVKFEIKGNPHFLMILPYNVGGAGAVRAMQIKGTRTQWIAMKKNWGQIWSTGVVLTGQCLSFRLTTSDGVMKEFIDVTPPDWKCNGQSFDGKVNF

>AtEXPA25

MKLLEQMVYVECFMIIMATLLVSMSYGHRAMINDVAEAPVIDNVGSPTNGLDSSWYDARATFYGDIHGGETQQGACGYGDLFKQGYGLETAALSTALFNEGYTCGACYQIMCVHDPQWCLPGTIKITATNFCPPDYSKTEGVWCNPPQKHFDLSLPMFLKIAQYKAGVVPVKYRRISCARTGGVKFETKGNPYFLMILPYNVGGAGDIKLMQVKGDKTGWITMQKNWGQNWTTGVNLTGQGISFRVTTSDGVTKDFNNVMPNNWGFGQTFDGKINF

>AtEXPA26

MKLLEKMIYVEFLMIIMAMWVVPMSYGHGAMIGNAVEAPDVAEAPGINDPSKALDPNWYDARATFYGDIHGGDTQQGACGYGNLFRQGYGLATAALSTALFNDGYTCGACYEIMCTRDPQWCLPGSVKITATNFCPANYSKTTDLWCNPPQKHFDLSLAMFLKIAKYKAGVVPVRYRRIPCSKTGGVKFETKGNPYFLMVLIYNVGGAGDIKYVQVKENKTGWITMKKNWGQNWTTSTVLTGQGLSFRVTTTDGITKDFWNVMPKNWGFGQTFDGKINF

>AtEXPB1

MQLFPVILPTLCVFLHLLISGSGSTPPLTHSNQQVAATRWLPATATWYGSAEGDGSSGGACGYGSLVDVKPFKARVGAVSPILFKGGEGCGACYKVRCLDKTICSKRAVTIIATDQSPSGPSAKAKHTHFDLSGAAFGHMAIPGHNGVIRNRGLLNILYRRTACKYRGKNIAFHVNAGSTDYWLSLLIEYEDGEGDIGSMHIRQAGSKEWISMKHIWGANWCIVEGPLKGPFSVKLTTLSNNKTLSATDVIPSNWVPKATYTSRLNFSPVL

>AtEXPB2

MTILVVDRYYMLMNLLFALTCLLLNLTHCFSPKKFNISAATTSDSDWSIAGSTWYGNPTGYGSDGGACGYGNAVAQPPFSKMVSAGGPSLFKSGKGCGACYQVKCTSKSACSKNPVTVVITDECPGCVKESVHFDLSGTAFGAMAISGQDSQLRNVGELQILYKKVECNYIGKTVTFQVDKGSNANSFAVLVAYVNGDGEIGRIELKQALDSDKWLSMSQSWGAVWKLDVSSPLRAPLSLRVTSLESGKTVVASNVIPANWQPGAIYKSNVNF

>AtEXPB3

MQLFPVMLATLCIVLQLLIGSSALATTNRHVSNSHWLPAVATWYGSPNGDGSDGGACGYGTLVDVKPLHARVGAVNPILFKNGEGCGACYKVRCLDKSICSRRAVTVIITDECPGCSKTSTHFDLSGAVFGRLAIAGESGPLRNRGLIPVIYRRTACKYRGKNIAFHVNEGSTDFWLSLLVEFEDGEGDIGSMHIRQAGAREWLEMKHVWGANWCIIGGPLKGPFSIKLTTLSAGKTLSATDVVPRNWAPKATYSSRLNFSPVL

>AtEXPB4

MASSQRYFALLALFAVSLKFCYCQNETIDVAGSGTAGVTWYGEPFGAGSTGGACGYGSAVANPPLYAMVSAGGPSLFNNGKGCGTCYQVVCIGHPACSGSPITVTITDECPGGPCASEPVHIDLSGKAMGALAKPGQADQLRSAGVIRVNYKRAACLYRGTNIVFRMDAGANPYYISFVVEYENGDGDLSNVEIQPAGGSFISMQEMRSAVWKVNSGSALRGPFNIRLTSGESHKVIVAYNVIPANWKPDESYRSIVNF

>AtEXPB5

MASSSLKCFSFIVVLTTFFAISLKPCYCHNKTHWNTAGITWYGDREGPGTTGGACGYGDAVAKHPYRCMVSAGGPSLFKDGKGCGACYRLKCDHPLCTKKPIKVMISDECPGCTKESVHFDLSGKAFGALAKRGKGDQLRNLGELKVSYKRACCKHPKTMIAIHVDAGANPYYMSFAVKFANGDGNFACIEVQPAGGQYMKMEEMRSAVWRLSPGVPLKGPFNIRLTSAVSGKKIIAKGVIPEKWSPGAIYHSKVNFPVQRKQK

>AtEXPB6

MIGELDFPFLGAGSTGGACGFAVANPPLYGMVSAGGPSVFNNGIGCGTCFQILCNGHPACSRRPITVTITDECPGGPCASEPAHFDLSGKAMGALARPGQGDRLRSAGVLRVYYRRVECLYRRTNIAFRMDPGANPYYISFVVEYENGDGDLAYIEIQPADGEFIPMQEMRSAVWKISSGSPLTGPFNIRLTSAESHKVVLAYNVIPANWKPNETYRSVVNFK

>AtEXLA1

MGSFLFLIVVIFLFSSSVNACDRCLHRSKAAYFSSASALSSGACAYGSMATSFFAGHIAAAIPSIYKDGAGCGACFQVRCKNPKLCSTKGTIVMITDLNKSNQTDLVLSSRAFRAMAKPIVGADKDLLKQGIVDIEYQRVPCDYGNKNMNVRVEEASKKPNYLEIKLLYQGGQTEVVSIDIAQVGSSPNWGYMTRSHGAVWVTDKVPTGAIQFRFVVTGGYDGKMIWSQSVLPSNWEAGKIYDAGVQITDIAQEGCDPCDAHIWN

>AtEXLA2

MLQGFLFLLSVVLLFSSSAAACDRCLHSSKAAYFSSASALSSGACAYGSMATGFFAGHIAAALPSIYKDGSGCGACFQVRCKNPTLCSSKGTTVIVTDLNKTNQTDLVLSSRAFRAMAKPVVGADRDLLKQGIVDIEYRRVPCDYGNKKMNVRVEESSKNPNYLAIKLLYQGGQTEVVAIYIAQVGSSHWSYMTRSHGAVWVTDKVPNGALQFRFVVTAGYDGKMVWSQRVLPANWEAGKSYDAGVQITDIAQEGCDPCDDHIWN

>AtEXLA3

MRSFLYLIVVIFLFSSSVNACDRCLHRSKASYFSSASALSSGACAYGPMATSFFAGHIAAAIPSIYKDGAGCGACFQVRCKNPKLCNSKGTIVMVTDLNTSNQTDLVLSSRAFRAMAKPVVGVDKYLLKQGIVDVEYQRVPCNYGKRNLNVRVEEASKKPNYLAIKLLYQGGQTEVVGIDIAPVGSSQWSYMSRSHGAVWATDKVPTGALQFKFTVTGGYDGKTVWSKRVLPANWNSGRIYDAGVQITDIAQEGCDTCGHIWN

>AtEXLB1

MKHSHVLLLLFVQVIVLLPLLCLSDDFVNSRATYYGSPDCKANPRGHCGYGEFGRDINNGEVSGVSWRLWNNGTGCGACYQVRCKIPPHCSEEGVYVVATDSGEGDGTDFILSPKAYGRMARPGTENQLYSFGVVNVEYQRIPCRYAGYNLVYKIHEKSYNPHYLAILVLYVGGVNDILAVEVWQEDCKEWRRMRRVFGAVHDLQNPPRGTLTLRFLVYGSAGINWIQSPNAIPADWTAGATYDSNILLT

>OsEXPA1

MAGSSAATSCARFLALLATCLLWNEAASFTASGWNKAFATFYGGSDASGTMGGACGYGDLYSTGYGTNTAALSTVLFNDGASCGQCYRIMCDYQADRRFCISGTSVTITATNLCPPNYALPNDAGGWCNPPRQHFDMAEPAWLKIGVYVGGIVPVMYQRVPCAKQGGVRFTINGRDYFELVLVSNVGGVGSIQSVSIKGSRTGWMAMSRNWGVNWQSNAYLDGQSLSFKVTSSDGQTLTFLDVAPAGWTFGQTFSTSQQFS

>OsEXPA2

MASRSSALLLLFSAFCFLARRAAADYGSWQSAHATFYGGGDASGTMGGACGYGNLYSTGYGTNTAALSTVLFNDGAACGSCYELRCDNDGQWCLPGSVTVTATNLCPPNYALPNDDGGWCNPPRPHFDMAEPAFLQIGVYRAGIVPVSYRRVPCVKKGGIRFTINGHSYFNLVLVTNVAGPGDVQSVSIKGSSTGWQPMSRNWGQNWQSNSYLDGQSLSFQVAVSDGRTVTSNNVVPAGWQFGQTFEGGQF

>OsEXPA3

MLSGMEKQPAMLLVLVTLCAFACKRSVAQSAFATFYGGKDGSGTMGGACGYGNLYNAGYGLYNAALSSALFNDGAMCGACYTITCDTSQTKWCKPGGNSITITATNLCPPNWALPSNSGGWCNPPRQHFDMSQPAWENIAVYQAGIVPVNYKRVPCQRSGGIRFAISGHDYFELVTVTNVGGSGVVAQMSIKGSNTGWMAMSRNWGANWQSNAYLAGQSLSFIVQLDDGRKVTAWNVAPSNWFFGATYSTSWVQF

>OsEXPA4

MAIAGVLFLLFLARQASAAGYGGWQSAHATFYGGGDASGTMGGACGYGNLYSQGYGTNTAALSTALFNDGAACGSCYELRCDNAGSSCLPGSITVTATNFCPPNYGLPSDDGGWCNPPRPHFDMAEPAFLHIAQYRAGIVPVSFRRVPCVKKGGVRFTVNGHSYFNLVLVTNVAGAGDVRSVSIKGSRTGWQPMSRNWGQNWQSNAFLDGQSLSFQVTASDGRTVTSNNVAHPGWQFGQTFEGGQF

>OsEXPA5

MSSRRDVLAVVLVAALLPPALSRGLWLGHHGLGHGHGRWRAPHVGGHGQGQGPQQHAPLGGGGWSSAHATFYGGGDASGTMGGACGYGNLYSQGYGTNTAALSTALFNNGLSCGACFEVRCDAGGGGSHSCLPGSVVVTATNFCPPNNALPSDDGGWCNPPRAHFDMSQPVFQRIALFKAGIVPVSYRRVACQKKGGIRFTINGHSYFNLVLVTNVGGAGDVHAVAVKSERSAAWQALSRNWGQNWQSAALLDGQALSFRVTTGDGRSVVSNNAVPRGWSFGQTFSGAQFN

>OsEXPA6

MAPPLLLLLASLLLVAARRALGLGLGQWQPGHATFYGGGDASGTMGGACGYGNLYSQGYGTSTAALSTALFNRGLSCGSCYELRCAGDHRRSCLPGGATVTVTATNFCPPNYALPSDGGGWCNPPRRHFDLAEPAFLRIARHAAGIVPVSFRRVACARKGGVRFTVNGHAYFNLVLVTNVGGAGDVRSLAVKGSGSGSRVGGRWQPMSRNWGQNWQSNAYLDGKALSFRVTAGDGRSLTCADVAPAGWQFGQTFEGRQF

>OsEXPA7

MSPAPRVLVLVVATVVALQVSPAAGRIPGAYGGGEWQSAHATFYGGSDASGTMGGACGYGNLYSQGYGVNNAALSTALFNSGQSCGACFEIKCVNQPGWEWCHPGSPSILITATNFCPPNYALPSDNGGWCNPPRPHFDLAMPMFLHIAEYRAGIVPVSYRRVPCRKKGGVRFTINGFRYFNLVLITNVAGAGDIVRASVKGTSTGWMPMSRNWGQNWQSNSVLVGQALSFRVTGSDRRTSTSWNAAPAGWHFGQTFEGKNFRV

>OsEXPA8

MAAARMLVLLASLCALLLTASAAKWTPAFATFYGGSDASGTMGGACGYGDLYGAGYGTRTAALSTALFNGGASCGACFTIACDTRKTQWCKPGTSITVTATNFCPPNYALSGDAGGWCNPPRRHFDMSQPAWETIAVYRAGIVPVNYRRVPCQRSGGIRFAVNGHSYFELVLVTNVGGSGAVAQMWIKGSGTGWMAMSRNWGANWQSNARLDGQALSFRVQADDGRVVTAADVAPAGWSFGATYTSSAQFY

>OsEXPA9

MEKKLLVVLFLSLCCASRLRGEAAQQWTSATATFYGGSDASGTMGGSCGYGNMYSAGYGTNTTALSSALYGDGASCGACYLVTCDASATRWCKNGTSVTVTATNYCPPNYSESGDAGGWCNPPRRHFDMSQPAWEAIAVYSSGIVPVRYARTPCRRVGGIRFGIAGHDYYELVLVTNVAGSGAVAAAWVKGSGTEWL

SMSRNWGENWQSNAYLTGQALSFRVQADDGGVVTAYDVAPANWQFGSTYQSDVNFSY

>OsEXPA10

MAPCLLLVLFLLPALATGHQHPSTLGSSALSEWRSAKASYYAADPEDAIGGACGFGDLGKHGYGMATVGLSTALFERGAACGGCYEVKCVDDLKYCLPGTSIVVTATNFCAPNFGLPADAGGVCNPPNHHFLLPIQSFEKIALWKAGVMPIQYRRVNCLRDGGVRFAVAGRSFFLTVLISNVGGAGDVRSVKIKGTESGWLSMGRNWGQIWHINSDFRGQPLSFELTSSDGKTLTNYNVVPKEWDFGKTYTGKQFLL

>OsEXPA11

MAAEVAAGGDSGWSSGSATFYGGSDASGTMGGACGYGNLYSAGYGTSTAALSTALFNNGQSCGACFEVRCGGGGSCLAGTVAVTATNLCPPNYALAGDAGGWCNPPRPHFDMAEPAFTRIAQARAGVVPVQYRRVACAKQGGIRFTITGHSYFNLVLVTNVGGAGDVTAVSVKGSRSGWQAMSHNWGANWQNGANLDGQPLSFRVTASDGRTVTSDNVAPSGWSFGQTFSGGQF

>OsEXPA12

MARSAFFFHCVAAVAACIAATAAALSGTATFYGGSDASGTMGGACGYGNLYSTGYGTNTAALSSALFNDGAACGECYQITCDQSNSKWCKAGTSVTITATNLCPPDYSKPSNDGGWCNPPRQHFDMAQPAWEQIGVYRGGIVPVNFQRVSCTRKGGVRFTINGNSYFELVLITNVGGPGSIKSVQIKGTKTGWVTMSRNWGANWQANNYLNNQAISFSVTSTAGKTLVFEDVAPSNWQFGQTFTSGVQFY

>OsEXPA13

MAGVARMLAAVVCAIMPAAAMAAGGVGALEPSGWVRAHATFYGGADASGTMGGACGYGNLYAQGYGTRTAALSTALFNDGLACGQCYKLVCDRKTDRTWCKPGVSVTITATNFCPPNWDLPSDSGGWCNPPRPHFDMAQPAWEKIGIYRGGIIPVIYQRVPCMKKGGVRFTINGHDYFQLVLLTNVGAAGSIKAMDVKGSKSPDWMAMAHNWGAQWHSLAYLTGQGLSFRVTITDGQTLVFPNVVRPGWRFGQTFASNIQFK

>OsEXPA14

MASSPRAFALVFFAIAAVGCTQLTTADDAAPPVWQKAHATFYGGADASGTMGGGCGYGDLYSQGYGTRNAALSTALFNDGASCGQCYKIACDRKRAPQWCKPGVTVTITATNFCPPNWDLPSDNGGWCNPPRPHFDMAQPAWEKIGIYSAGIIPVIYQRVPCIKKGGVRFTINGHDYFNLVLVTNVATTGSIKSMDIMGSNSTDWMPMVRNWGANWHSLSYLTGQTLSFRVTNMDGQTLVFKNIVPSGWKFGQTFTSKLQFK

>OsEXPA15

MWKKKKTPSILPLVVVIAAASLIAPTTAGWSSGTATFYGGSDASGTMGGACGYGNLYWSGYGTNTAALSSALFNDGASCGQCYQIACDHQAEPRWCLQGRTVTITGTNLCPPNYALSSNDGGWCNPPRTHFDMAEPAWLQIGIYKAGIVPVLYQRVPCVKQGGVRFTMGGFNYFELVLISNVAGSGSIQSVWVKGPNTDRMPLSRNWGANWQSHAGLVGQTLTFGVTSTGGQTLVFQNIVPAWWKFGQSFSSNLQFSY

>OsEXPA16

MSSVLLFLLLLLLSGVSLSGCIRLGNGGYEEWRMGSATYIKESLGHPLNDGGGACGYGDLDIFRYGRYTAGVSGALFGRGSACGGCYEVRCVNHVLWCLRGSPTVVVTATDFCAPNLGLSDDYGGWCNFPKEHFEMSEAAFLRVAKAKADIVPVQFRRVSCDRAGGMRFTITGGASFLQVLITNVAADGEVAAVKVKGSRTGWIPMGRNWGQNWQCDADLRGQPLSFEVTGGRGRTVVAYSVAPPDWMFAQTFEGKQFVE

>OsEXPA17

MASSWNNPAIFLAAALAVATAAQVVTAGFTTDLYWQQQPAPGAVTPYKTSDWHDGSATFYGDPSGMGDDFGGACGYVSNDIVSLYSTKTAALSTPLFADGNGCGQCYELRCVKSPWCNPGSPSVVITGTNLCPPNWYLPNDDGGWCNPPRHHFDMAPPSFLKLAQRVAGIVPVQYRRVPCQRTGGVRFCLQGNHYWLLLYVMNVGGAGDVSSLSVKTSGGGGAWIQAAHNWGITYQVFAALDNSDGLTVKLTTYSTPQQTIIVSDAISPWWITGLCYQGSNNFY

>OsEXPA18

MGNIVLQLLAILALCIAPARSGWLQGTATFYGGADGSGTMGGACGYGNLYDQGYGINNAALSTPLFNNGASCGQCYLIICNYDKAPSGCRMGTAITVTGTNFCPPNYDLPYGGWCNTTRPHFDMSQPAWENIGIYSAGIVPILYQQVKCWRSGGVRFTITGLNYFELVLVTNMAGSGSIASMSVKGSSTGWIQMSRNWGANWQCLAGLAGQALSFTVTSTGGQTIVFDSVVPAGWSFGQTFSTYQQFDY

>OsEXPA19

MGNIFLQLLAVVALCIAPARSDWLPGTATFYGGADGSGTMGGACGYGNLYDQGYGINNAALSTPLFNDGASCGQCYLIICDYSKAPDWCKLGKAITVTGTNYCPPNYDLPYGGWCNATRPHFDMSQPAWENIGIYNAGIIPILYQQVKCWRYGGVRFTINGFNYFELVLVTNMAGSGSIASMSVKGSCTGWIQMTRNWGANWQCLAGLAGQALSFNVTSTGGQTIVFDDAVPAGWSFGQTFSTYHQFDY

>OsEXPA20

MGNILLQLLAVVALCIAPARSDWLPGTATFYGGADGSGTMGGACGYGNLYDQRYGINNAALSTPLFNDGASCGQCYLIICDYGKAPDWCKLGKAITVTGTNYGGWCNATRPYFDMSQPAWENIGIYSAGIVPILYQQVKCWRYGGVRFIINGFNYFELVLVTNMAGSGSIVSMSVKGSCTGWIQMTRNWGANWQCLAGLAGQALSFNVTSTGGQTIVFDDAVPAGWSFGQTFSTYHQFDY

>OsEXPA21

MAPPSLPILLVLLSLSSSLSSSSAAAAGRWTDAHATFYGGADASGTMGGACGYGNTYGQGYGTDTAALSAVMFGDGLSCGACFELRCGGGGGGDRRGCLPPAAGKSIVVTATDLCPANHALPGDRGGWCNPPLHHFDLSQPAFLRIARFQSGIVPVSYRRVACRRKGGMRFTINGHSYFNLVLVSNVGGAGDVHAVAVKAGGGRKARWQAMARNWGQNWQSGALLDGQALSFTVTTGDRRSVVSYNVAPAGWAFGQTFTGRQFT

>OsEXPA22

MAPARPFALLFLAVTVGFVLLTAADDSANATATTTTAMAPSSSTDDAAPPVWLKAHATFYGGADASGTMGGACGYGDLYSQGYGTRNAALSTALFNDGASCGQCYKIACDRKRAPQWCRPGVTVTITATNFCPPNWDLPSDNGGWCNPPRPHFDMAQPAWEKIGIYRAGIIPVIYQRVPCVKKGGVRFTINGHDYFNLVLVTNVATTGLIKSMDVMGSNSTDWLPMVRNWGANWHSLSYLTGQMLSFRVTNMDGQTLVFRNIVPSGWKFGQTFASKLQFK

>OsEXPA23a

MAPARAFVLVLLAVASASTAAANTATTTPTNPVAAPTQWQKAHATFYGGADASGTMGGACGYGNLYSQGYGTRNAALSTALFNDGASCGQCYKIACDRKRAPQWCKPGVTVTITATNFCPPNWNLPSDNGGWCNPPRPHFDMAQPAWEKIGVYSAGIIPVIYQRVPCVKKGGLRFTINGHDYFQLVLVTNVAAAGSIKSMEVMGSNTADWMPMARNWGAQWHSLAYLTGQGLSFRVTNTDDQTLVFTNVVPPGWKFGQTFASKLQFK

>OsEXPA23b

MAPARAFVLVLLAVASASTAAANTATTTPTNPVAAPTQWQKAHATFYGGADASGTMGGACGYGNLYSQGYGTRNAALSTALFNDGASCGQCYKIACDRKRAPQWCKPGVTVTITATNFCPPNWNLPSDNGGWCNPPRPHFDMAQPAWEKIGVYSAGIIPVIYQRYQYKYSNYFGHYGENSAYPDRCFHMKISYSCDDFYRVPCVKKGGLRFTINGHDYFQLVLVTNVAAAGSIKSMEVMGSNTADWMPMARNWGAQWHSLAYLTGQGLSFRVTNTDDQTLVFTNVVPPGWKFGQTFASKLQFK

>OsEXPA24

MADMAPARALALVLLAVAVGSALMAAAQDAPSPPTPMAPSPSTDETPPVWLKAHATFYGGADASGTMGGACGYVDLYSQGYGTRNAALSTALFNDGASCGQCYKIACDRKRAPQWCKPGVTVTVTATNFCPPNWNLPSDNGGWCNPPRPHFDMAQPAWEKIGIYRAGIIPVMYQRVPCVKKGGVRFTINGHDYFNLVLVTNVATTGSIKSMDIMGSNSTDWMPMVRNWGANWHSLSYLTGQMLSFRVTNMDGQTLVFRNIVPSGWKFGQTFASKLQFK

>OsEXPA25

MEYAILFATSLVITVLAASGFAPAHGWNKGTATFYGGADASGTMGGACGYGNLYTAGYGTNTAALSSVLFNDGWSCGQCYLIMCDAAATPQWCRAGAAVTITATNLCPPNWALPSNSGGWCNPPRPHFDMAEPAWLQIGIYKAGIIPVLYQQVKCWRQGGIRFTMGGFNFFELVLVSNVAGSGSVRSVSVKGGSTGWITLNRNWGANWQCNSGLVGQALSFAVTSTGGQTLYIYNVVPSWWSFGMTFTSNQQFSY

>OsEXPA26

MDTTTTMAPLPLLTTTSLLLFFFLASSFAADVVVAGGGGGGGGYDGGGDGEGGGGGDGEGGGGGGGAKMPHVNHGRYKCGPWVDGHATFYGGRDASGTTEGGACGYKDADGYGAMTAAVSPALFDNGAGCGACYELKGDSGKTVVVTATNQAPPPVNGMKGEHFDLTMPAFLSIAEEKLGVVPVSYRKVACVRQGGIKYTITGNPSYNMVMVKNVGGAGDVVKLTVKGTKRVKWTPLQRSWGQLWKTEANLTGESLTFRVMTGDHRKATSWRVAPRDWTYDNTYQAKKNF

>OsEXPA27

MGAMAENLLVLCTILAARMALAAADDWIPATATFYGGNDGSGTMGGACGYGNLYDQGYGLENAALSTALFNDGAACGQCYLIVCDTDKAGRWCKPRGAVTVTATNLCPPNWALPSDGGGWCNPPRRHFDMSQPAWERIGVYRAGIVPVLYRRVRCWRRGGVRFTVGGFDHFELVLVANVAGSGSVAAVSVRGAGTGWLQMSRNWGANWQSLAGLAGQPLSFGVTTTGGQYILFQDVAPAGWKFGQTFSTSKQFDY

>OsEXPA28

MMVIRFFAVLAAALCITSASAAAAGGWVSGTATFYGGKDASGTMGGACGYGNLYTQGYGVYNAALSTALFNGGASCGQCYLIMCDASKTPEWCKAGTAVTITATNLCPPNWALANDDGGWCNPPRPHFDMSQPAWETIGIYRAGIVPVLYQQVKCWRQGGVRFTVSGFNYFELVLITNVAGSGSVQAMSVKGSKTGWIPLARNWGANWQCNSALVGQALSFRVTSTGGQTLQINSVVPEWWEFGTTFTSNQQFDY

>OsEXPA29

MARRGHVFAVVAFVSYALLAAASTTVEAFAASGWSKGTATFYGGSDASGTMGGACGYGNLYTQGYGTRTAALSTALFDDGASCGQCYALTCDARADPRWCRAGASVTVTATNFCPPNYALPSDDGGWCNPPRPHFDMAQPAWERIGVYRGGIVPVAFRRVPCRRRGGVRFTVAGRDYFELVLVTNVAAAGSVRSMEVRGSRRGAGWMAMSRNWGANWQSLAYLDGQGLSFRVTATDGQTIVFAGVVPPSWRFGQTFASTQQFM

>OsEXPA30

MAAASSTTATTAILAAVIISLAGAATTVDAKFRAMQWTPAHATFYGDETASETMGGACGYGNLYASGYGTDTAALSTTLFKDGYGCGTCYQMRCVGTASCYRGSPAITVTATNLCPPNWAEDPDRGGGGWCNPPRAHFDLSKPAFMRMADWRAGIVPVMYRRVPCARAGGLRFALQGNPYWLLAYVMNVAGAGDVGDMWVKAGGGGGWVRMSHNWGASYQAFAQLGGQALSFKVTSYTTGQTILAAGVTPASWCFGLTYQARVNFS

>OsEXPA31

MDMAMSSRLALCLAVVAACAAGGAVADWSPATATFYGGSDGSGTMGGACGYGNLYDQGYGVDNAALSQALFNDGASCGQCYLIVCDTSRAPQWCKAGTAVTVTATNLCPPNWALPSDGGGWCNPPRPHFDMSQPAWEQIGVYQAGIVPVLYQRVRCWRQGGVRFTVAGLNYFELVLITNVAGSGSVASAWIKGTNTGWIQMSRNWGANWQSLAGLAGQALSFAVTTTGGQYLQFQDVAPAWWQFGQTFSTYQQFDY

>OsEXPA32

MWCTWALGRVVLAVVFLVALAAGDAAPPKVHRNHGKFTAGPWKQAHATFYGGRDGSGTLDGACGYKDTSKEGYGVQTVAVSTPLFGAGAGCGACYEVKCVDSPDGCKVGAAPLVVTATNLCPPNPGQSNDNGGWCNPPREHFDLSMPAFLQIAQEKAGIVPISYRRVPCVKVGGIRYTITGNPYFNLVMVSNVGGAGDVAGLSVKGNKRVKWTPLKRNWGQEWQTSEVLTGESLTFRVMTGDHRKATSWHVLPPDWQFGVTYQATKNFN

>OsEXPA33

MAMPVVQVLLLCALAYQAVDAQWTPATATFYGGSDGAGTMGGACGYGNLYNAGYGLNNAALSSALFNDGAMCGACYTIACDTSQSTWCKPGTSITITATNLCPPNYAKKSDAGGWCNPPRKHFDMSQPAWTSIAIYQAGIVPVNFKRVPCQKSGGIRFTISGRDYFELVTVFNVGGSGVVAQVSIKGSKTDWMAMSRNWGQNWQSNAYLNTQSLSFKVKLDDAREVTVWNIAPSNWNFGTTYTSNINF

>OsEXPB1a

MASSSLLLACVVVAAMVSAVSCGPPKVPPGPNITTSYGDKWLEAKATWYGAPKGAGPKDNGGACGYKDVDKAPFLGMNSCGNDPIFKDGKGCGSCFEIKCSKPEACSDKPALIHVTDMNDEPIAAYHFDLSGLAFGAMAKDGKDEELRKAGIIDTQFRRVKCKYPADTKITFHIEKASNPNYLALLVKYVAGDGDVVEVEIKEKGSEEWKALKESWGAIWRIDTPKPLKGPFSVRVTTEGGEKIIAEDAIPDGWKADSVYKSNVQAK

>OsEXPB1b

MASSSLLLACVVVAAMVSAVSCGPPKVPPGPNITTSYGDKWLEAKATWYGAPKGAGPKDNGGACGYKDVDKAPFLGMNSCGNDPIFKDGKGCGSCFEIKCSKPEACSDKPALIHVTDMNDEPIAAYHFDLSGLAFGAMAKDGKDEELRKAGIIDTQFRRVKCKYPADTKITFHIEKASNPNYLALLVKYVAGDGDVVEVEIKEKGSEEWKALKESWGAIWRIDTPKPLKGPFSVRVTTEGGEKIIAEDAIPDGWKADSVYKSNVQAK

>OsEXPB2

MAGASAKVVAMLLSVLATYGFAAGVVYTNDWLPAKATWYGQPNGAGPDDNGGACGFKNTNQYPFMSMTSCGNEPLFQDGKGCGACYQIRCTNNPSCSGQPRTVIITDMNYYPVARYHFDLSGTAFGAMARPGLNDQLRHAGIIDIQFRRVPCYHRGLYVNFHVEAGSNPVYLAVLVEFANKDGTVVQLDVMESLPSGKPTRVWTPMRRSWGSIWRLDANHRLQGPFSLRMVSESGQTVIAHQVIPANWRANTNYGSKVQFR

>OsEXPB3

MAFSISKKAAVAALFSFLVVTCVAGARPGNFSASDFTADPNWEVARATWYGAPTGAGPDDDGGACGFKNTNQYPFSSMTSCGNEPIFKDGKGCGSCYQIRCVNHPACSGNPETVIITDMNYYPVSKYHFDLSGTAFGAMAKPGQNDQLRHAGIIDIQFKRVPCNFPGLKVTFHVEEGSNPVYFAVLVEYEDGDGDVVQVDLMEANSQSWTPMRESWGSIWRLDSNHRLTAPFSLRITNESGKQLVASQVIPANWAPMAVYRSFVQYSS

>OsEXPB4

MGSLSSLAAAAVFLSLLAVGHCAAADFNATDADADFAGNGVDFNSSDAAVYWGPWTKARATWYGQPNGAGPDDNGGACGFKHTNQYPFMSMTSCGNQPLFKDGKGCGSCYKIRCTKDQSCSGRSETVIITDMNYYPVAPFHFDLSGTAFGRLAKPGLNDKLRHSGIIDIEFTRVPCEFPGLKIGFHVEEYSNPVYFAVLVEYEDGDGDVVQVDLMESKTAHGPPTGRWTPMRESWGSIWRLDTNHRLQAPFSIRIRNESGKTLVANNVIPANWRPNTFYRSFVQYS

>OsEXPB5

MVSRGTFVFAVLVALPILSLPVSGYEQNYTAGRRSTMSLGRGYGWSSGGATWYGGPQGDGSEGGACGYQSAVGQRPFSSMIAAGGPSLFKNGKGCGSCYQIKCTGNRACSGRPVTVVITDSCPGGVCLNEAAHFDMSGTAFGAMANRGMGDRLRSAGVLKIQYKRVPCRFAMNVAFKVDAGSNPYYLAILVQYANGDGDLAAVHIMEARGGGGWKAMQQSWGATWRLNSNTGKPLSPPFSIRLTSGSGKVLVANNVIPSGWQAGLTYRSTVNYAA

>OsEXPB6

MAARMGSKVAAILAILSVLVVHGSCKGHPVNYNVSDASAYGSGWLPARATWYGAPTGAGPDDNGGACGFKNVNQYPFSSMTSCGNEPIFKDGKGCGSCYQIRCNKDPSCSGNIETVIITDMNYYPVARYHFDLSGTAFGAMAKPGLNDKLRHSGIIDIQFRRVPCNYPGLKINFHVEEGSNPVYFAVLVEYEDLDGDVVQVDLMESKSAYGGATGVWTPMRESWGSIWRLDSNHRLQAPFSLRIRSDSGKTLVANNVIPANWSPNSNYRSIVQFS

>OsEXPB7

MAGRSRRRSFWSVGVAAALLCLLAAHGCSAKHHKPKPTPGGISGNASSSSSNSSTPSIPPPVAPTPTAPTPPIPSPGTGSSNGSSGGGGGGWLNARATWYGAPNGAGPDDNGGACGFKNVNLPPFSAMTSCGNEPLFKDGKGCGSCYQIRCVGHPACSGLPETVIITDMNYYPVSLYHFDLSGTAFGAMAKDNRNDELRHAGIIDIQFRRVPCQYPGLTVTFHVEQGSNPVYMAILVEYENGDGDVVQVDLMESRYSTGGVDGTPTGVWTPMRESWGSIWRLDTNHPLQGPFSLRITNESGKTLIADQVIPADWQPNTVYSSIVQFD

>OsEXPB8

MVSGDVGVVVYYLLLVLVVVQGCKGSSAVQGEGRWYNESEAIGGAAAWGNAKATWYGQPNGAGAADNGGACGFKKVNQYPFMGMTSCGNQPLYKGGKGCGSCYRVRCNRNPACSGNAQTVAITDMNYFPLSQYHFDLSGIAFGRLAKPGRADDLRRAGIIDVQFARVPCEFPGLKVGFHVEEGSSPVYLAVLVEYENGDGDVAQVDLKEAGAGGGRWTPMRESWGSVWRLDSNHRLRAPFSIRIRSDSGKTLVAPDVIPLNWTPNTFYRSFVQYSS

>OsEXPB9

MGSLTTNIVLAVAVVAALVGGGSCGPPKVPPGPNITTNYNAPWLPARATWYGQPYGSGSTDNGGACGIKNVNLPPYNGMISCGNVPIFKDGRGCGSCYEVKCEQPAACSKQPVTVFITDMNYEPISAYHFDFSGKAFGAMACPGKETELRKAGIIDMQFRRVRCKYPGGQKVTFHVEKGSNPNYLAVLVKFVADDGDVIQMDLQEAGLPAWRPMKLSWGAIWRMDTATPLKAPFSIRVTTESGKSLIAKDVIPVNWMPDAIYVSNVQFY

>OsEXPB10

MASSCLLLACVVAAAMVSAVSCGPPKVPPGPNITAAYGKQWLEARGTWYGKPKGAGPDDNGGACGYKDIDKAPFLGMNSCGNDPIFKDGKGCGSCFEVKCSKPEACSDKPVIIHITDMNTEPIAAYHFDLSGHAFGAMAKEGKDEELRKAGIIDMQFRRVRCKYPGETKVTFHVEKGSNPNYFAVLVKYVGGDGDVVKVELKEKGSEEWKPLNESWGAIWRIDTPKPLKGPFSLRVTTESDQKLVANDVIPDNWKANALYKSEIQVD

>OsEXPB11

MAKSCTLVLLLVALVGLSLLVSPIACSRKLSKPKPKPKPSMKKPVVRAHNNYTGSPSVTVTTGWAAAGATYYGAPNGDGSDGGACGYQTAVGQRPFSSMIAAGSPSLYKGGKGCGACYEVKCTTNAACSGQPATVVITDECPGGICLAGAAHFDMSGTSMGAMAKPGMADKLRAAGILQVQYRRVPCKYSGVNIAFRVDQGANPFYFEVLIEFEDGDGDLNAVDLMEAGCGWTPMVQNWGALWRYNSNTGKALKAPFSLRLTSDSGKVLVANNVIPASWKPGVTYRSLVNYS

>OsEXPB12

MPAAAPRVASAASGSASATRGVRVARDKSGGGKLEERTAVAVAVKVVVVDPQRLRMAAFEPHRVQHMASRLQLLYFIAITVLASVFQPCTSIELHRELSGWSNGIATWYGDPNGAGSEGGACGYQYAVDQPPFSSRIAAGSPYIYDSGKGCGSCYRVVCAGNEACSGIPVTVVITDQGPGGPCLEELVDGQCMNEAAHFDMSGTAFGAMARPGQADQLRGAGLLQIQYTRVECEWTGVGLTFVVDSGSNPNYLALLVEYDDNDSDLAAVDIMPIGAGASGSWIPMQQSWGAVWRLNSGSALQGPFSVRLTFSSGQMFVASNAIPAGWNPGMAYQPGGVAMRVRGRNGGRRGYEAVGMLGGLCHLLLLLLLMLFEL

>OsEXPB13

MASSSLLLASVVVAAMVSAVSCGPPKVPPGPNITASYGDKWLEARATWYGAAKGAGRKDNSGACGYKDVDKAPFLGMNSCGNDPIFKDGKGCGSCFEIKCSKPKACSDKPVLIHVTDMNDEPIAAYHFDLFGLAFGAMAKDGKDEELLKYVAGDGDVVEVEIKEKGSEEWKALKESWGAIWRIDTPKPLKGPFSVRVTTEGGEKIIAEDAIPDGWKADSVYKSNVQAK

>OsEXPB14

MALAAKLLPSIVAFVALACCVLRSSVASVDHHRKLSGWSIGGATWYGPANGSGTDGGACGYQGDVGQPPFNSMIAAGSPSIYESGKGCGSCYQVKCSGNPSCSGKPVTVVLTDLCPGGACLEEPVHFDLSGTAFGAMAKPGQDDQLRNAGKLPVQYARVPCKWQGVDIAFRVDAGSNQYYLAVLVEDEDGDGDLSAVDLMQSGGSGGGGSWAAMQQSWGAVWKYNSGPAPLQAPMSIRLTSGSGRTLVASNVIPAGWQPGGTYRSIVNFRRED

>OsEXPB15

MASRFQLILSTFVVIAAVTMLPRPCASIEFHRKLSSWSNGGATWYGAANGAGSDGGACGYQGAVFQAPFSSMIAAGSPSIYKSGLGCGSCYQVKCTGNSACSGNPVTVVLTDECPGGPCLSEPVHFDLSGTAFGAMANPGQADQLRAAGVLQIQYNRVPCNWGGVKLTFVVDVGSNPNYFAVLVKYENGDGDLSGVELMQTGAGAAWTQMQQSWGAVWKLNAGSALQAPFSIRLTSSSGKTLVASNVIPSGWKPGMSYISTVNF

>OsEXPB16

MAAFSSSSSAPMLIRSVLFVSLLSAAFVFDSGEAGAAHRVVDPEWHPATATWYGSADGDGSDGGACGYGTLVDVVPMKTRVGAVSPVLFKGGEGCGACYKVRCLDASICSRRAVTVIVTDECPGGVCAFGRTHFDLSGAAFARLAVAGHGGQLQNRGEISVVYRRTACKYGGKNIAFHVNEGSTTFWLSLLVEFEDGDGDIGSMQLKQANSAQWQDMKHIWGATWSLTPGPLVGPFSVRLTTLTTRQTLSAQDVIPKNWTPKATYTSRLNFA

>OsEXPB17

MAAASSRSFSLCVLLLLLLLAPPISASFLFDGGKSKSAAAAAAVDMEWRPATATWYGDAEGDGSTGGACGYGSLVDVVPMKARVGSVSPVLFKDGEGCGACYKVKCLDHGICSRRAVTVIVTDECPGGLCAFGRTHFDLSGAAFSRMAVAGAGGHLRDRGQLSVVYRSVQIYQDCVQVRREEHSLPCKRGLDELLAVTAGRVRGRPGRHWIHADKAGELSGVAGHEACVGGHVVPRAGPACRALLGEVDDAVGPEGAHGPGRHPQELEAHGHLHLAPQLRGRPLIGWGSAGPGPTFYRPGKLPGGPAKAGPTWQSVRQESQASSIVMQLIILVGLIIHDSIHG

>OsEXPB18

MNSKFQLILSTFVVIAAFTLLPRPCASIEFHRKLSSWSNGGATWYGAANGAGSDGGACGYQAAVDQAPFSSMIAAGSPSIYKSGLGCGSCYQVKCSGNSACSGNPVTVVLTDECPGGPCLSEPVHFDLSGTAFGAMANPGQADQLRAAGVLQIQYNRVPCNWGGVMLTFAVDAGSNPSYFAVLVKYENGDGDLSGMDLMQTGAGAAWTPMQQSWGAVWKLSAGAALQAPLSIRLTSSSGKTLVASNVIPSGWKPGASYTSTVNY

>OsEXLA1

MAVSVRCCFGSSSLSHHARLLLVIVALLAPRLASGCDRCVRRSRAAYYTSSLTLTAGSCGYGTAAATFNGGGFLAAAGPALYRGGVGCGACYQVRCKDKKLCSNAGARVVVTDRARTNRTGLVLSSPAFAAMARPGMAASLTELAAVDVEYKRVPCEYRHRSLSVRVDERSRGPNELTISFLYQGGQTDIVAVDVAQVGSSSWKFMTREHGPSWSMANAPPGPLQMRLVVTGGYDGKWVWADREVLPRRWRAGEVYDTGVQITDIAQEGCFPCDTHEWK

>OsEXLA2

MAVRCCSSMASASVVLFFVVVGMSASMVSGCDRCVRRSKAGFRDSSIALNAGSCGYGSLAASFNGGHLAAASPALFRGGVGCGACFQVRCKDGKLCSTAGAKVVVTDEARSTNRTDLVLSAAAYAAMARPGMAAQLRTRRAVDVEYKRVPCEYAAGRNLSIRVEEKSRPPRELSIRFLYQGGQTDIVAVDVATVGSSNWKFMTRDYGPAWSTAQAPAGPLQFRVVVTGGYDGKWVWADGEVLPRRWTAGRVYDAGVQIADVAQEGCYPCDTQEWK

>OsEXLA3

MAVLLSILSSSFLLLLAASSSSTPRASACERCVRNGKAAYSPSLSPLPPGGGGGCGYGAMAMEMELNGGFLAAGGPRQHRGGLGCGRCFQMRCRNAEVCSNAGVRVVLTDFHRSNSTDFLLGGPAFAGLAKPGMAHKLKKLDALSVEYRRIPCDYKDKNLSILVEEQSKRPNNLVIKFLYQGGQTDILAVDVAQVGSSDWRFMTRVYGPVWSIDRAPNGPLQFRAVVTGGYDGKWVWADREVLPANWQPGQVYDTGARIADVARESCLDCATLDWK

>OsEXLA4

MDDNGDVHFCHRATAVVALLLLHLVVVANAAAHSCDWCTPRHSTVSILPTPTHAAHLTGGACGFGAAPMELNVAAVTADLFRHGHACGACYQLRCRDRRLCGEDGVKVVVADMAKQPEQEGEMNRTAGGSLQFRITEDAFAAMAKQGVSAHELTRQRTLEVDFRRIPCEYRESRRLAVRVEEASRNPTHLAIRFLYQGGQTDIAAVEIAQANATPPSSSYYSSWRYMTRRDGAPGVWTTSRAPVGPLRLRVVVTAGSGGKWLRSDGEVLPADWRPGEVYDTGLRVTDVAVRSCSLSCAIQDMDSDDGEEEELR

>OsEXLB1

MAQLLRRHLPVILSLILFLSKATADANFTVSRAAYYPNSDIKGTENGACEYGAFGATLNNGDVSASASLYRDGVGCGACYQVRCTNPYYCSPNGVTIVITDSGASDGTDFILSQHAFTRMAQSTDAGTALLTLGVVGIEYRRVSCTYPNKNIVFKITESSNFPNYLEFEIWYQQGNQDIIAVQLCETVNLTCQLLSRTHGAVWAAVSPPSGPLSIRMLFSSGAPRGGDTWLVPTNIVPQNWTAGATYDSGVQVQLQ
